# Association between various insulin resistance indices and cardiovascular disease in middle-aged and elderly individuals: evidence from two prospectives nationwide cohort surveys

**DOI:** 10.3389/fendo.2024.1483468

**Published:** 2024-11-22

**Authors:** Yan Li, Huijuan Li, Xiaoyu Chen, Xueyan Liang

**Affiliations:** ^1^ Department of Clinical Pharmacy, Guangxi Academy of Medical Sciences and the People’s Hospital of Guangxi Zhuang Autonomous Region, Nanning, Guangxi, China; ^2^ Phase 1 Clinical Trial Laboratory, Guangxi Academy of Medical Sciences and the People’s Hospital of Guangxi Zhuang Autonomous Region, Nanning, Guangxi, China

**Keywords:** insulin resistance, cardiovascular disease, estimated glucose disposal rate, triglyceride glucose, triglyceride to high-density lipoprotein cholesterol ratio, metabolic score for insulin resistance

## Abstract

**Background:**

The estimated glucose disposal rate (eGDR), triglyceride glucose (TyG), triglyceride to high-density lipoprotein cholesterol (TG/HDL-C) ratio, and metabolic score for insulin resistance (METS-IR) are dependent indicators of insulin resistance (IR). We aimed to evaluate the association between these indicators and the current or feature incidence of cardiovascular disease (CVD) in middle-aged and elderly individuals. This study tests the hypothesis that IR indices positively or negatively correlate with CVD, and that the potential predictive performance of the IR indices was not the same.

**Methods:**

Middle-aged and elderly individuals from the National Health and Nutrition Examination Survey (NHANES) and the China Health and Retirement Longitudinal Study (CHARLS) with complete data on eGDR, TyG, TG/HDL-C, and METS-IR at baseline were obtained. The association between the four indices and CVD was evaluated using multivariate logistic regression analysis. In addition, an adjusted restricted cubic spline (RCS) was applied. Finally, the potential predictive performance of the IR indices was assessed using receiver operating characteristic (ROC) curves.

**Results:**

We included 7,220 participants (mean age: 61.9 ± 10.7 years; 54.0% male) from the NHANES cohort and 6,426 participants (mean age: 57.9 ± 8.4 years; 45.2% male) from the CHARLS cohort in the study. Multivariate logistic regression analysis indicated that a decreasing eGDR significantly increased the incidence of CVD both presently and in the future. Similarly, a higher TyG level and METS-IR were significantly associated with a higher incidence of CVD at both timeframes. However, the TG/HDL-C ratio was not significantly associated with CVD, heart disease, or stroke. No significant interactions were observed between the continuous or quartile variables of eGDR, TyG, TG/HDL-C, or METS-IR, and the incidence of various endpoints across most subgroups. The ROC curve indicated the superior predictive performance of the IR indices. Furthermore, the eGDR was superior to other IR indices for the prediction of CVD both at present and in the future in middle-aged and elderly individuals.

**Conclusion:**

As continuous variables, eGDR, TyG, and METS-IR were significantly associated with the incidence of CVD, both currently and in the future, among middle-aged and elderly individuals. Notably, incorporating eGDR, TyG, or METS-IR and the basic model significantly increased the predictive value for CVD. Among these indices, the eGDR index stands out as the most promising parameter for predicting CVD, both at present and in the future.

## Introduction

Cardiovascular disease (CVD) is, a significant global health issue that continues to be a leading cause of mortality and economic burden, especially in developing countries (1–4). Despite significant progress in the prevention, diagnosis, and treatment of CVD in recent years, its global incidence continues to rise globally (5). Therefore, it is essential to enhance the current indices for the precise recognition of patients with a high risk of CVD and risk reduction of CVD.

Insulin resistance (IR), a pathophysiological condition marked by a reduced tissue response to insulin (6–8), results in impaired utilization of blood glucose (9). Previous studies have shown that IR serves as both a causative factor and an indicator of poor prognosis in patients with CVD, irrespective of their diabetic status (7, 10, 11). Although the exact biological mechanisms connecting IR to CVD are not fully understood, several potential mechanisms have been suggested, including metabolic disturbances, oxidative stress, endothelial dysfunction, heightened inflammation, and inappropriate activation of the renin-angiotensin-aldosterone system (12, 13). Considering these detrimental effects, various methods have been proposed to evaluate IR. Although the hyperinsulinemic-euglycemic (HIEG) clamp is widely regarded as the gold standard for assessing IR, its complex testing process limits its clinical utility (14). For example, clinical practicability and feasibility are constrained by their time-intensive and laborious nature (15). Similarly, the homeostasis model assessment for insulin resistance (HOMA-IR) is not well suited for large cohort evaluations because of its economic burden and tedious operability (16).

Recently, several simpler IR indices have been proposed, including the estimated glucose disposal rate (eGDR), triglyceride glucose (TyG) level, triglyceride to high-density lipoprotein cholesterol (TG/HDL-C) ratio, and metabolic score for insulin resistance (METS-IR). In addition, these indicators are practical for clinical use (17). Numerous studies have identified the eGDR, TyG, TG/HDL-C ratio, and METS-IR as key indicators for predicting CVD onset (18–22). However, each IR index captures various aspects of IR. The eGDR calculation, which incorporates waist circumference (WC), glycosylated hemoglobin A1c (HbA1c), and the presence of hypertension, highlights the association between obesity, hypertension, and glucose metabolism (18). TG are a storage form of lipids used for energy production. HDL-C is a beneficial type of cholesterol that carries excess cholesterol from the body back to the liver for excretion or reuse (23). Thus, the TG/HDL-C ratio serves as an indicator of IR in lipid metabolism. TyG, derived from fasting triglyceride and glucose levels, illustrates the interaction between lipid and glucose metabolism (24). The TyG index was determined by incorporating fasting blood glucose (FBG), TG, body mass index (BMI), and HDL-C levels (25). TyG reflects complex interactions between obesity, lipid metabolism, and glucose metabolism. Therefore, we propose that the predictive performances of these IR indices vary. Although these IR indices have been studied across different cohorts, a comparison of their predictive performance in CVD is still lacking. Moreover, these studies predominantly focused on the future incidence of CVD. However, there is still a lack of research focusing on both the current and future incidences of CVD in middle-aged and elderly individuals. To bridge these knowledge gaps, we included participants from the National Health and Nutrition Examination Survey (NHANES) and the China Health and Retirement Longitudinal Study (CHARLS) and aimed to compare the effectiveness of these parameters in predicting CVD in the present and future to ultimately provide clinicians with a more accurate predictive tool.

## Methods

### Study design and population

**Figure 1:**
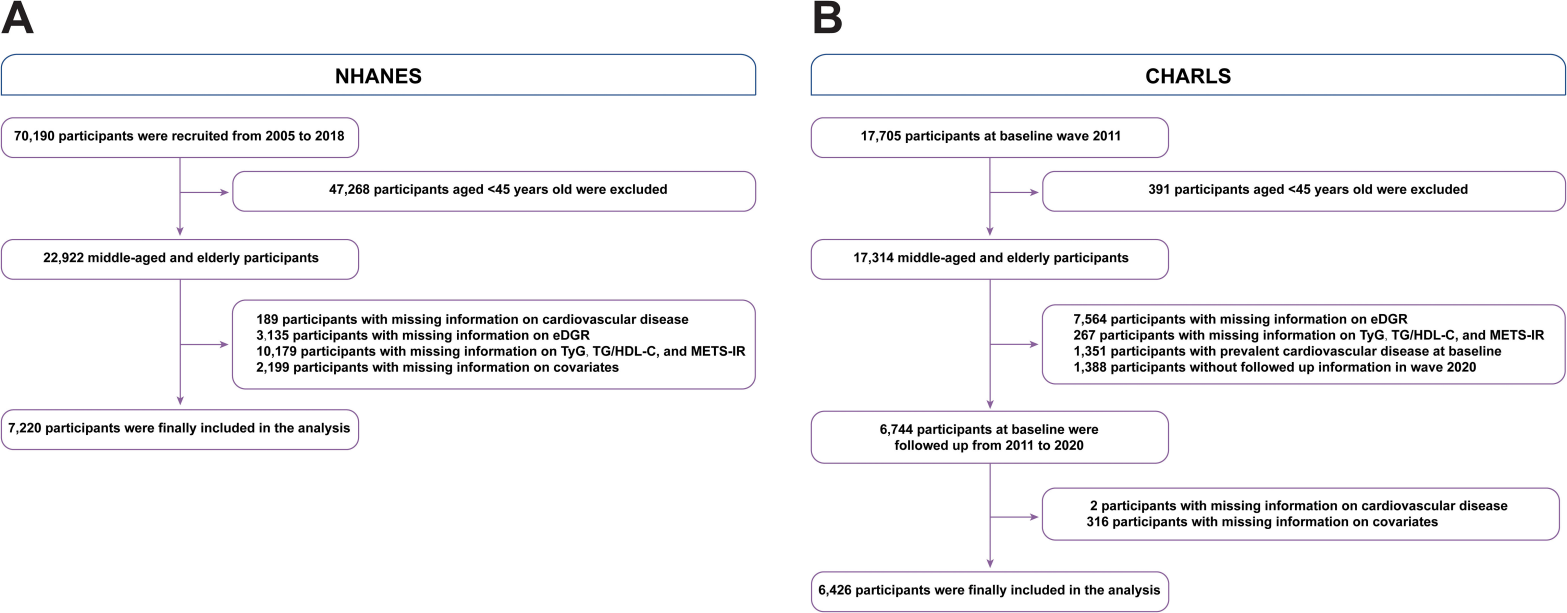
Flow diagram of patient selection. **(A)** NHANES, **(B)** CHARLS. eGDR, estimated glucose disposal rate; TyG, triglyceride glucose; TG, triglyceride; HDL-C, high-density lipoprotein cholesterol; METS-IR, metabolic score for insulin resistance; NHANES, National Health and Nutrition Examination Survey; OR, odd ratio.

We gathered participants from the NHANES and CHARLS cohort studies, focusing on US and Chinese residents aged 45 years and above. The survey design and extensive data collection methods of the NHANES have been previously described (26). This study focused on individuals who reported having CVD and had complete baseline data on eGDR, TyG, TG/HDL-C, and METS-IR. These data were used to examine the association between IR indices and the current incidence of CVD. Accordingly, 22,922 individuals aged 45 years and above were obtained from the NHANES surveys between 2005 and 2018. Participants with missing information on CVD (n=189), eGDR (n=3,135), TyG, TG/HDL-C ratio, METS-IR (n=10,179), or other covariates (n=2,199) were excluded from the analysis (**Figure 1A**). A total of 44,462 participants from NHANES were included. We included participants with or without prevalent CVD to evaluate the relationship between CVD and IR indices in the NHANES.

Regarding the CHARLS, a detailed survey design and enrollment criteria were documented (27). A baseline survey was conducted from June 2011 to March 2012, selecting a nationally representative sample of 17,705 participants from 10,229 households. Participants were regularly followed up every two years through face-to-face interviews performed by the interviewer using a computer to administer and record responses to survey questions. The follow-up survey waves were performed in 2013, 2015, 2018, and 2020. We included participants who were not diagnosed with CVD at baseline and had complete follow-up results for IR indices. The incidence of CVD was assessed during follow-up investigations using, the latest data collected in Wave 4 of 2020. The CHARLS data were used to evaluate the association between these IR indices and the future incidence of CVD. Consequently, 17,314 individuals aged 45 years and above from the CHARLS were included. Participants with missing information on eGDR (n=7,564), TyG, TG/HDL-C, and METS-IR (n=267); those without follow-up information in the 2020 wave (n=1,388); those diagnosed with CVD at baseline (n=2), and those missing other covariates (n=316) were excluded from the analysis (**Figure 1B**). For the analysis of future CVD incidence among the CHARLS participants, we included only those who were free of prevalent CVD at baseline.

### Data collection and definition

During the NHANES examination, certified examiners measured blood pressure (BP) multiple times using standardized protocols. Four readings were averaged to determine the BP. Additionally, body mass, height, and WC data were obtained. The BP of the participants were expressed as the average of several measurements in the NHANES and CHARLS. Blood samples were obtained from participants in both NHANES and CHARLS, and further measurements were conducted according to standard procedures. The biochemical parameters measured included blood urea nitrogen (BUN), uric acid (UA), hemoglobin, HbA1c, total cholesterol (TC), HDL-C, TG, and low-density lipoprotein cholesterol (LDL-C). Other covariates included age, sex, education, marital status, alcohol consumption, and smoking status (current smoker, former smoker [NHANES only], never smoker [NHANES only], or non-smoker [CHARLS only]).

Hypertension was diagnosed in participants who either self-reported a diagnosis of hypertension, were taking prescribed antihypertensive medications, or had a BP reading of ≥140/90 mmHg (28). The BMI was calculated using the following formula: BMI (kg/m^2^) = body mass/height^2^. Obesity was defined as a BMI of 28 kg/m² or higher.

### Insulin resistance index calculation

Four IR indices were evaluated to predict the CVD incidence: eGDR, TyG, TG/HDL-C ratio, and METS-IR. The formula for calculating eGDR was as follows: 21.158 − (0.09 × WC) − (3.407 × hypertension) − (0.551 × HbA1c) [WC (cm), hypertension (yes = 1/no = 0), and HbA1c (%)] (18, 29). TyG was using from the formula: ln[TG (mg/dL) × FBG (mg/dL)/2] (23). The TG/HDL-C ratio was calculated by dividing TG (mg/dL) by HDL-C (mg/dL) (23). METS-IR was calculated as follows: ln[(2 × FBG (mg/dL) + TG (mg/dL))× BMI/ln[HDL-C (mg/dL)] (30).

### Outcome ascertainment

In this study, the primary outcome was CVD, encompassing both current and future heart disease and stroke. In line with standard precedents (31–33), incident CVD was identified through self-reports in which participants confirmed receiving a definitive diagnosis of CVD from their physicians. In NHANES, incident CVD data were collected during each survey cycle. Participants in the CHARLS were followed from the baseline in 2011 until the occurrence of CVD or until the most recent survey (2020), whichever occurred first.

### Statistical analysis

Continuous variables are presented as mean ± standard deviation (SD) or median (interquartile range). Multivariate logistic regression analysis is a statistical tool that can be used to select and combine input variables linked to a certain outcome. Multivariate logistic regression models were used to evaluate the odds ratios (ORs) and 95% confidence intervals (CIs) for the relationships between the eGDR, TyG, TG/HDL-C, and METS-IR indices and CVD. Three multivariate models were constructed: Model 1 was an unadjusted model; Model 2 was adjusted for age, sex, marital status, education, smoking, and alcohol consumption status; and Model 3 was further adjusted region, TC, HDL-C, TG, LDL-C, BUN, UA, hemoglobin, and obesity. Restricted cubic splines (RCS) can be advocated as a potential alternative to these modelling strategies (categorizing a continuous variable or imposing the assumption of a linear association on a continuous variable) to explore non-linear continuous associations. The RCS has an additional property: the curve is linear before the first knot and after the last knot. RCS logistic regression analysis was used to explore the linearity and dose-response relationship between eGDR, TyG, TG/HDL-C, METS-IR and CVD. P for non-linear was cleated, and P for non-linear (<0.05) was considered a non-linear relationship. Covariates of interest and potential confounders were selected *a priori* based on the biological rationale and preexisting knowledge of risk factors for CVD, which can be obtained from NHANES and CHARLS. In this study, covariates were adjusted across the three models, with eGDR, TyG, TG/HDL-C, or METS-IR values at an OR of one serving as the reference point. Receiver operating characteristic (ROC) curves were generated to evaluate the predictive performance of the eGDR, TyG, TG/HDL-C, or METS-IR for CVD. The C-statistic was used to quantify the predictive value (34, 35). To further assess the enhanced predictive performance relative to the basic models, net reclassification improvement (NRI) and integrated discrimination improvement (IDI) indices were calculated (36). Decision curve analysis was used to compare the clinical benefits. Subgroup analyses were performed to evaluate the effect of eGDR, TyG, TG/HDL-C, and METS-IR (continuous and categorical variables) on CVD across various subgroups, which included age (< 60/≥ 60 years), sex (male/female), smoking status (current smoker/former smoker/never smoker/non-smoker), alcohol consumption (drinker/non-drinker), and obesity (yes/no). Sensitivity analysis was performed to assess the robustness of the results. The relationship between eGDR, TyG, TG/HDL-C, METS-IRand CVD was examined among participants without diabetes mellitus in model 3. Diabetes mellitus was defined in participants who either self-reported a diagnosis of diabetes mellitus, were taking prescribed antidiabetic medications, or had elevated FBG and HbA1c levels. All analyses were performed using R, version 4.2.1. Two-sided P values <0.05 were considered statistically significant.

## Results

### Participant characteristics

This study included 7,220 participants from the NHANES cohort (mean age: 61.9 ± 10.7 years, 54.0% male) and 6,426 participants from the CHARLS cohort (mean age: 57.9 ± 8.44 years, 45.2% male). In the final NHANES cohort sample, the mean values were as follows: eGDR was 6.71 ± 2.60, TyG was 8.70 ± 0.61, TG/HDL-C was 2.61 ± 2.01, and METS-IR was 7.81 ± 0.42. For the CHARLS cohort, the mean values were: eGDR was 9.48 ± 2.26, TyG was 8.67 ± 0.65, TG/HDL-C was 3.13 ± 4.02, and METS-IR was 7.59 ± 0.41. **Table 1** offers further details information on the characteristics of the included participants. Based on the results of NHANES, 1,265 cases of CVD were documented, including 1,006 cases of heart disease and 413 cases of stroke. As for CHARLS, there were 789 recorded cases of incident CVD, comprising 555 cases of heart disease and 276 cases of stroke.

**Table 1:** Baseline characteristics of participants.

Characteristics	NHANES (n = 7220)	CHARLS (n = 6426)
Age; years	61.9 ± 10.7	57.9 ± 8.4
45-54 years; n (%)	2169 (30.0)	2413 (37.6)
60-69 years; n (%)	2199 (30.5)	2664 (41.5)
70-75 years; n (%)	1699 (23.5)	1087 (16.9)
≥ 75 years; n (%)	1153 (16.0)	262 (4.1)
Sex; n (%)		
Male	3902 (54.0)	2906 (45.2)
Female	3318 (46.0)	3520 (54.8)
SBP; mmHg	129 ± 19.2	129 ± 20.7
DBP; mmHg	69.7 ± 13.6	75.4 ± 12.0
BMI; kg/m2	29.3 ± 6.39	23.5 ± 3.75
WC; cm	102 ± 15.3	83.8 ± 12.6
Education; n (%)		
Junior high school and below	780 (10.8)	5798 (90.2)
Senior high school	2729 (37.8)	558 (8.7)
Tertiary	3711 (51.4)	70 (1.1)
Marital status, n (%)		
Married	4284 (59.3%)	5822 (90.6)
Other	2936 (40.7%)	604 (9.4)
Alcohol consumption; n (%)		
Drinker	5121 (70.9)	2174 (33.8)
Non-drinker	2099 (29.1)	4252 (66.2)
Smoking; n (%)		
Current smoker	1454 (20.1)	2407 (37.5)
Former smoker	2587 (35.8)	NA
Never smoker	3179 (44.0)	NA
Non-smoker	NA	4019 (62.5)
Hemoglobin; g/dL	14.2 ± 1.51	14.4 ± 2.21
FBG; mg/dL	115 ± 37.1	109 ± 32.5
HbA1c; %	5.97 ± 1.12	5.24 ± 0.75
TC; mg/dL	196 ± 43.2	194 ± 376
TG; mg/dl	123 ± 65.2	130 ± 95.7
HDL-C; mg/dL	55.2 ± 16.8	51.6 ± 15.1
LDL-C; mg/dL	116 ± 37.1	117 ± 34.5
BUN; mg/dL	14.9 ± 6.27	15.7 ± 4.37
UA; mg/dL	5.68 ± 1.43	4.39 ± 1.21
Serum creatinine; mg/dL	0.944 ± 0.45	0.769 ± 0.18
Obesity; n (%)	3750 (51.9)	699 (10.9)
Hypertension; n (%)	4219 (49.3)	2342 (36.4)
eGFR; mL/min/1.73 m^2^	92.3 ± 23.1	103.3 ± 16.1
Prediabetes, n (%)	544 (9.7)	NA
Diabetes, n (%)	1992 (27.6)	365 (4.2)
Kidney disease, n (%)	296 (4.1)	368 (5.7)
eGDR	6.71 ± 2.60	9.48 ± 2.26
TyG	8.70 ± 0.61	8.67 ± 0.65
TG/HDL-C	2.61 ± 2.01	3.13 ± 4.02
METS-IR	7.81 ± 0.42	7.59 ± 0.41

Continuous variables are expressed as mean ± SD (standard deviation), and categorical variables as N (%).

BMI, body mass index; BUN, blood urea nitrogen; DBP, diastolic blood pressure; eGDR, estimated glucose disposal rate; FBG, fasting blood glucose; HbA1c, glycosylated hemoglobin A1c; HDL-C, high density lipoprotein cholesterol; LDL-C, low density lipoprotein cholesterol; METS-IR, metabolic score for insulin resistance; SBP, systolic blood pressure; TC, total cholesterol; TG, triglycerides; TyG, triglyceride glucose; UA, uric acid; WC, waist circumference.

### Associations between the eGDR index and CVD

**Table 2** showed a significant relationship between the eGDR and the current or feature incidence of CVD when eGDR was analyzed as a continuous variable. In the NHANES study, higher eGDR were related to a lower risk of current CVD across all models, with OR and 95%CI of 0.78 (0.76, 0.80), 0.79 (0.77, 0.82), and 0.83 (0.81, 0.86), respectively. The results demonstrated a significant relationship between eGDR and both current heart disease or stroke, as shown in **Supplementary Tables S1** and **S2**. Compared to the lowest quartile (Q1) of eGDR, the ORs and 95% CIs for Q2−Q4 were 0.75 (0.63, 0.90), 0.51 (0.41, 0.62), and 0.30 (0.23, 0.39) for current CVD risk; 0.80 (0.66, 0.98), 0.53 (0.42, 0.66), and 0.33 (0.24, 0.44) for current heart disease risk; and 0.77 (0.58, 1.02), 0.59 (0.43, 0.80), and 0.27 (0.17, 0.42) for current stroke risk. These RCS curves for model 3 revealed a nonlinear association between eGDR and the risk of current CVD and heart disease (P-values for nonlinearity < 0.05, **Supplementary Figure S1**). However, a linear relationship was observed between eGDR and current stroke in both models (P-values for nonlinearity > 0.05).

**Table 2:** Association of eGDR, TyG, TG/HDL-C, and METS-IR with cardiovascular disease.

Index	NHANES						CHARLS					
	Model 1		Model 2		Model 3		Model 1		Model 2		Model 3	
	OR (95% CI)	P value	OR (95% CI)	P value	OR (95% CI)	P value	OR (95% CI)	P value	OR (95% CI)	P value	OR (95% CI)	P value
eGDR												
Continues	0.78 (0.76, 0.80)	< 0.001	0.79 (0.77, 0.82)	< 0.001	0.83 (0.81, 0.86)	< 0.001	0.88 (0.85, 0.91)	< 0.001	0.89 (0.86, 0.92)	< 0.001	0.91 (0.88, 0.94)	< 0.001
Quartiles												
Q1	Ref		Ref		Ref		Ref		Ref		Ref	
Q2	0.68 (0.59, 0.79)	< 0.001	0.63 (0.53, 0.74)	< 0.001	0.75 (0.63, 0.90)	0.002	0.69 (0.57, 0.84)	< 0.001	0.71 (0.58, 0.86)	< 0.001	0.75 (0.61, 0.91)	0.005
Q3	0.39 (0.33, 0.46)	< 0.001	0.40 (0.33, 0.48)	< 0.001	0.51 (0.41, 0.62)	< 0.001	0.54 (0.44, 0.67)	< 0.001	0.59 (0.48, 0.73)	< 0.001	0.64 (0.51, 0.80)	< 0.001
Q4	0.16 (0.13, 0.20)	< 0.001	0.21 (0.17, 0.26)	< 0.001	0.30 (0.23, 0.39)	< 0.001	0.49 (0.39, 0.60)	< 0.001	0.52 (0.42, 0.65)	< 0.001	0.59 (0.47, 0.74)	< 0.001
P for trend		< 0.001		< 0.001		< 0.001		< 0.001		< 0.001		< 0.001
TyG												
Continues	1.44 (1.30, 1.59)	< 0.001	1.42 (1.28, 1.58)	< 0.001	1.46 (1.16, 1.84)	0.001	1.27 (1.14, 1.42)	< 0.001	1.27 (1.14, 1.42)	< 0.001	1.33 (1.04, 1.70)	0.022
Quartiles												
Q1	Ref		Ref		Ref		Ref		Ref		Ref	
Q2	0.98 (0.82, 1.18)	0.840	0.92 (0.76, 1.12)	0.410	0.93 (0.76, 1.15)	0.520	1.26 (1.00, 1.58)	0.049	1.24 (0.99, 1.56)	0.063	1.16 (0.92, 1.47)	0.210
Q3	1.21 (1.01, 1.44)	0.040	1.14 (0.94, 1.37)	0.180	1.11 (0.88, 1.41)	0.360	1.55 (1.25, 1.93)	< 0.001	1.50 (1.20, 1.88)	< 0.001	1.34 (1.05, 1.71)	0.021
Q4	1.67 (1.41, 1.98)	< 0.001	1.58 (1.32, 1.89)	< 0.001	1.40 (1.02, 1.92)	0.040	1.62 (1.30, 2.02)	< 0.001	1.61 (1.30, 2.01)	< 0.001	1.37 (1.01, 1.88)	0.047
P for trend		< 0.001		< 0.001		0.040		< 0.001		< 0.001		0.024
TG/HDL-C												
Continues	1.10 (1.07, 1.13)	< 0.001	1.10 (1.07, 1.13)	< 0.001	1.03 (0.95, 1.11)	0.500	1.01 (1.00, 1.03)	0.093	1.02 (1.00, 1.03)	0.049	0.97 (0.92, 1.02)	0.254
Quartiles												
Q1	Ref		Ref		Ref		Ref		Ref		Ref	
Q2	1.31 (1.09, 1.57)	0.004	1.24 (1.03, 1.50)	0.030	1.20 (0.95, 1.51)	0.120	1.00 (0.79, 1.25)	0.969	1.00 (0.80, 1.25)	0.999	0.92 (0.72, 1.19)	0.526
Q3	1.36 (1.14, 1.63)	< 0.001	1.26 (1.04, 1.53)	0.020	1.11 (0.84, 1.48)	0.470	1.40 (1.13, 1.73)	0.002	1.41 (1.14, 1.74)	0.002	1.20 (0.91, 1.59)	0.204
Q4	1.84 (1.55, 2.20)	< 0.001	1.82 (1.51, 2.20)	< 0.001	1.55 (1.03, 2.33)	0.040	1.36 (1.10, 1.68)	0.005	1.39 (1.12, 1.72)	0.003	1.11 (0.76, 1.62)	0.5901
P for trend		< 0.001		< 0.001		0.160		< 0.001		< 0.001		0.247
METS-IR												
Continues	2.14 (1.85, 2.47)	< 0.001	2.47 (2.11, 2.89)	< 0.001	2.03 (1.51, 2.71)	< 0.001	1.57 (1.32, 1.87)	< 0.001	1.64 (1.37, 1.95)	< 0.001	2.08 (1.45, 2.99)	0.0001
Quartiles												
Q1	Ref		Ref		Ref		Ref		Ref		Ref	
Q2	1.08 (0.90, 1.31)	0.410	1.08 (0.89, 1.32)	0.420	0.97 (0.77, 1.21)	0.780	1.23 (0.97, 1.55)	0.084	1.27 (1.00, 1.60)	0.047	1.25 (0.98, 1.59)	0.076
Q3	1.43 (1.19, 1.71)	< 0.001	1.43 (1.18, 1.73)	< 0.001	1.09 (0.83, 1.42)	0.540	1.68 (1.35, 2.09)	< 0.001	1.75 (1.40, 2.19)	< 0.001	1.70 (1.31, 2.20)	< 0.001
Q4	2.10 (1.77, 2.50)	< 0.001	2.37 (1.97, 2.85)	< 0.001	1.49 (1.08, 2.05)	0.020	1.74 (1.40, 2.16)	< 0.001	1.82 (1.46, 2.27)	< 0.001	1.73 (1.25, 2.40)	< 0.001
P for trend		< 0.001		< 0.001		0.007		< 0.001		< 0.001		< 0.001

Model 1: unadjusted.

Model 2: adjusted for age, sex, marital status, education, smoking, and alcohol consumption status.

Model 3: model 2 + further adjusted for region, total cholesterol, high density lipoprotein cholesterol, triglyceride, low density lipoprotein cholesterol, blood urea nitrogen, uric acid, hemoglobin, and obesity.

CHARLS, China Health and Retirement Longitudinal Study; CI, confidence interval; eGDR, estimated glucose disposal rate; TyG, triglyceride glucose; TG, triglyceride; HDL-C, high-density lipoprotein cholesterol; METS-IR, metabolic score for insulin resistance; NHANES, National Health and Nutrition Examination Survey; OR, odd ratio.

Similar findings were observed in the CHARLS study, where higher eGDR levels were associated with a lower risk of future CVD in both models. The OR and 95% CI were 0.88 (0.85, 0.91), 0.89 (0.86, 0.92), and 0.91 (0.88, 0.94), respectively. The association between eGDR and the future risk of heart disease or stroke was also evident in both models. Compared to the lowest quartile (Q1) of eGDR, the ORs and 95% CIs for Q2−Q4 were 0.75 (0.63, 0.90), 0.51 (0.41, 0.62), and 0.30 (0.23, 0.39) for future CVD incidence; 0.80 (0.66, 0.98), 0.53 (0.42, 0.66), and 0.33 (0.24, 0.44) for future heart disease incidence; and 0.77 (0.58, 1.02), 0.59 (0.43, 0.80), and 0.27 (0.17, 0.42) for future stroke incidence. The RCS regression models indicated that eGDR and the future incidence of CVD, heart disease, and stroke are linear (P for nonlinearity > 0.05, **Supplementary Figure S2**).

### Associations between the TyG and CVD

The results of NHANES revealed that higher TyG was related to a higher risk of current CVD. When the TyG was treated as a continuous variable, the unadjusted model revealed an OR of 1.44 (95% CI: 1.30, 1.59), and the fully adjusted model showed an OR of 1.46 (95% CI: 1.16, 1.84) (**Table 2**). A similar relationship between TyG levels and the current risk of heart disease was observed in both models (**Supplementary Tables S1**, **S2**). However, no significant relationship was observed between TyG and the current stroke. Participants in higher quartiles of TyG were found to have a higher risk of CVD in the current. Compared to the first quartile (Q1) of eGDR, the ORs with 95% CIs for the Q2−Q4 were as follows: 0.93 (0.76, 1.15), 1.11 (0.88, 1.41), and 1.40 (1.02, 1.92) for current CVD risk; and 0.95 (0.75, 1.20), 1.13 (0.88, 1.46), and 1.44 (1.02, 2.02) for current heart disease risk. Similar patterns were observed for TyG and the risk of stroke. The RCS regression models revealed that TyG and CVD or heart disease are nonlinear (P for non-linearity < 0.05, **Supplementary Figure S2**).

For CHARLS, the higher level of TyG was related to a higher future incidence of CVD in both models, with the ORs (95% CI) being 1.27 (1.14, 1.42), 1.27 (1.14, 1.42), and 1.33 (1.04, 1.70), respectively. The relationship between TyG and the future incidence of heart disease was observed in both models. However, no significant differences were found in the comparisons of TyG and the future risk of stroke. Compared to the fQ1 of TyG, the ORs with 95% CIs for future CVD incidence were 1.16 (0.92, 1.47) for Q2, 1.34 (1.05, 1.71) for Q3, and 1.37 (1.01, 1.88) for Q4. For future incidence of heart disease, the ORs were 1.07 (0.81, 1.40) for Q2, 1.26 (0.94, 1.68) for Q3, and 1.47 (1.02, 2.14) for Q4. For future stroke risk, only the Q4 of TyG showed a significant difference compared to the Q1, with an OR of 1.47 (1.02, 2.14). The RCS regression models revealed that TyG and the future incidence of CVD, heart disease, or stroke are linear (P for non-linearity > 0.05, **Supplementary Figure S2**).

### Associations between the TG/HDL-C and CVD

The results revealed that, according to the results of the NHANES and CHARLS, the TG/HDL-C ratio was not related to CVD, heart disease, or stroke in either the current or future context (**Table 2**; **Supplementary Tables S1**, **S2**). Similarly, when the TG/HDL-C ratio was treated as a nominal variable, most comparisons yielded consistent results. The RCS curves indicated a linear relationship between TG/HDL-C and the incidence of CVD, heart disease, or stroke, both currently and in the future, regardless of whether covariates were adjusted for (P for non-linearity > 0.05, **Supplementary Figure S3**).

### Associations between the METS-IR and CVD

NHANES results showed that higher METS-IR were related to a higher risk of current CVD. In the unadjusted model, the OR was 2.14 (95% CI: 1.85, 2.47), while in the fully adjusted model, the OR was 2.03 (95% CI: 1.51, 2.71). The relationship between METS-IR and current heart disease was also observed in both models (**Supplementary Tables S1**, **S2**). However, the fully adjusted model revealed no significant association between METS-IR and current stroke. When METS-IR was treated as a nominal variable, only the Q4 showed a significant difference compared to the Q1, with ORs of 1.49 (95% CI: 1.08, 2.05) for CVD and 1.63 (95% CI: 1.14, 2.32) for heart disease. Similar results revealed no significant relationship between METS-IR and current stroke. The RCS curves revealed a nonlinear relationship between METS-IR and current CVD or heart disease in the adjusted model (P for non-linearity < 0.05, **Supplementary Figure S4**).

In the CHARLS study, higher METS-IR were related to an increased incidence of future CVD in both models. Compared with Q1, the ORs with 95% CIs were 1.57 (1.32, 1.87) for Q2, 1.64 (1.37, 1.95) for Q3, and 2.08 (1.45, 2.99) for Q4. The association between METS-IR and the incidence of future heart disease and stroke was also revealed in both models. Compared to the Q1 of METS-IR, the fully adjusted ORs with 95% CIs for the Q2−Q4 were as follows: for future CVD: 1.25 (0.98, 1.59) for Q2, 1.70 (1.31, 2.20) for Q3, and 1.73 (1.25, 2.40) for Q4; for future heart disease: 1.06 (0.80, 1.40) for Q2, 1.50 (1.11, 2.01) for Q3, and 1.63 (1.11, 2.38) for Q4; for future stroke: 1.80 (1.18, 2.79) for Q2, 2.09 (1.34, 3.32) for Q3, and 1.91 (1.11, 3.31) for Q4. The RCS regression models revealed that METS-IR and the incidence of future CVD heart disease, and stroke are linear. (P for non-linearity > 0.05, **Supplementary Figure S4**).

### Subgroup and sensitivity analyses

**Figure 2:**
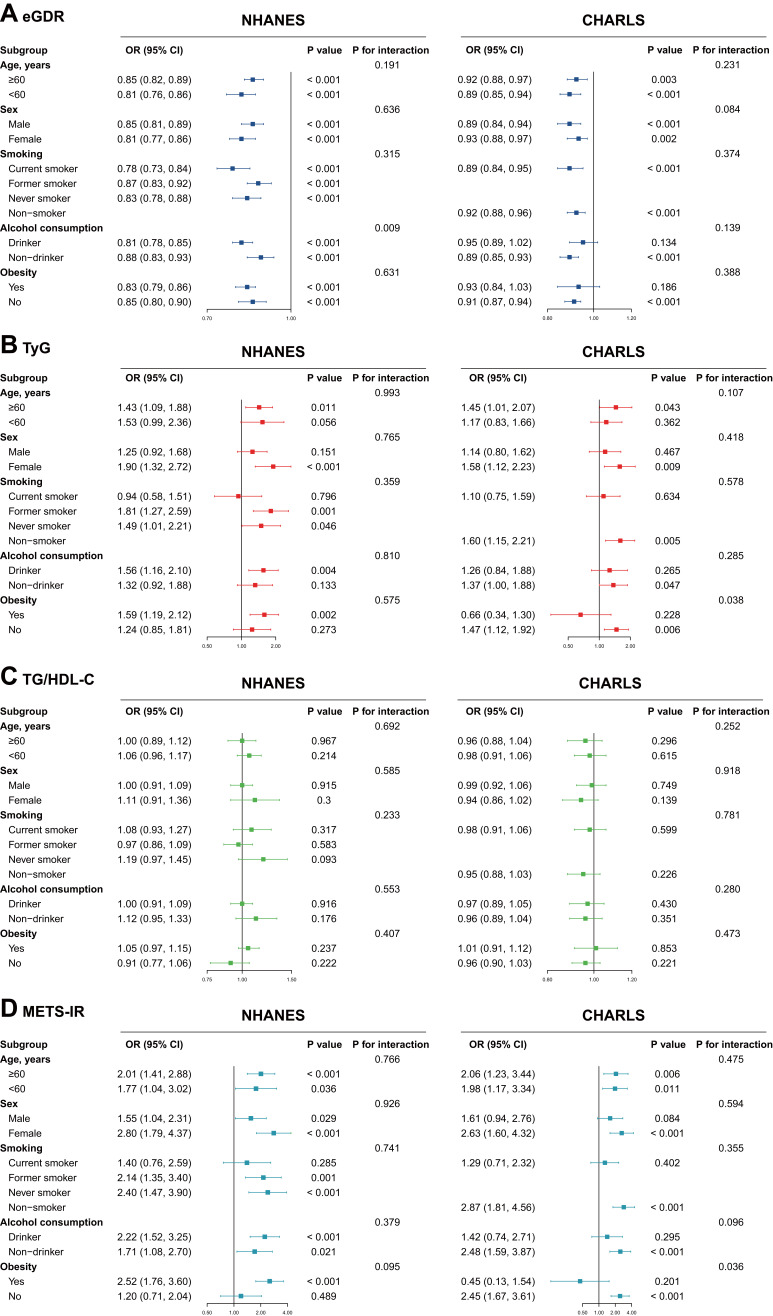
Subgroup analysis of the association between **(A)** eGDR, **(B)** TyG, **(C)** TG-HDL ratio, **(D)** METS-IR and cardiovascular disease. CHARLS, China Health and Retirement Longitudinal Study; CI, confidence interval; eGDR, estimated glucose disposal rate; TyG, triglyceride glucose; TG, triglyceride; HDL-C, high-density lipoprotein cholesterol; METS-IR, metabolic score for insulin resistance; NHANES, National Health and Nutrition Examination Survey; OR, odd ratio.

Stratified analyses were conducted to determine if the relationship between eGDR, TyG, TG/HDL-C, or METS-IR and current or feature CVD was not influenced by some of the subgroups. The association between eGDR, TyG, TG/HDL-C, or METS-IR and CVD was consistent with the main results across most subgroups (**Figure 2**). According to the NHANES results, the relationship between eGDR and current CVD risk varied across different subgroups of alcohol consumption (P for interaction = 0.009). According to CHARLS results, the relationship between METS-IR and future CVD incidence varied across different subgroups of obesity (P for interaction = 0.036). Similar patterns were observed for heart disease and stroke outcomes (**Supplementary Figures S5**, **S6**). No significant interactions were observed between quartiles of eGDR, TyG, TG/HDL-C, or METS-IR and the current risk and future incidence of various endpoints in most subgroups (**Supplementary Figures S7**-**S9**). In the sensitivity analyses, excluding participants with diabetes, as defined by FBG and HbA1c measurements, did not substantially alter the results (**Supplementary Table S3**).

### Incremental predictive performance and receiver operating characteristic curve analysis of eGDR, TyG, TG/HDL-C or METS-IR in CVD

**Figure 3:**
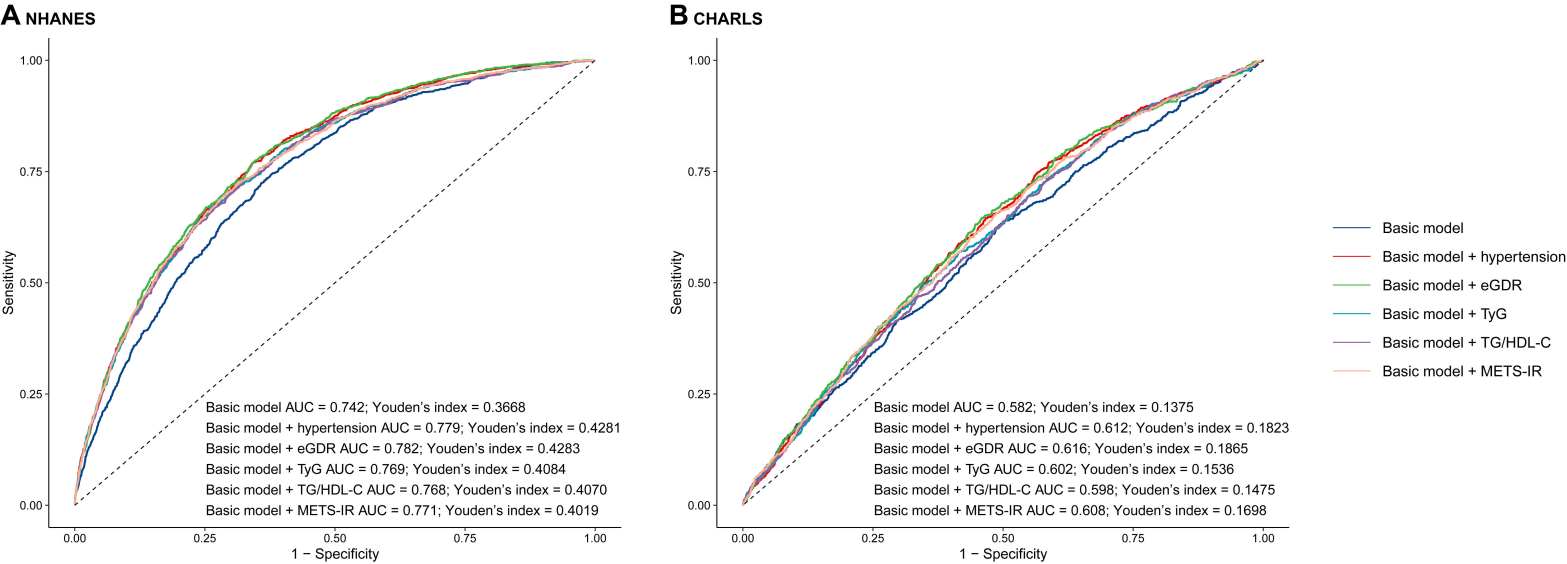
The receiver operating characteristic curves of eGDR, TyG, TG/HDL-C and METS-IR to predict cardiovascular disease. **(A)** NHANES, **(B)** CHARLS. The basic model adjusted age, sex, marital status, education, smoking, alcohol consumption status, total cholesterol, high density lipoprotein cholesterol, triglyceride, low density lipoprotein cholesterol, blood urea nitrogen, uric acid, hemoglobin, and obesity. AUC, area under curve; eGDR, estimated glucose disposal rate; TyG, triglyceride glucose; HDL-C, high-density lipoprotein cholesterol; METS-IR, metabolic score for insulin resistance.

**Figure 4:**
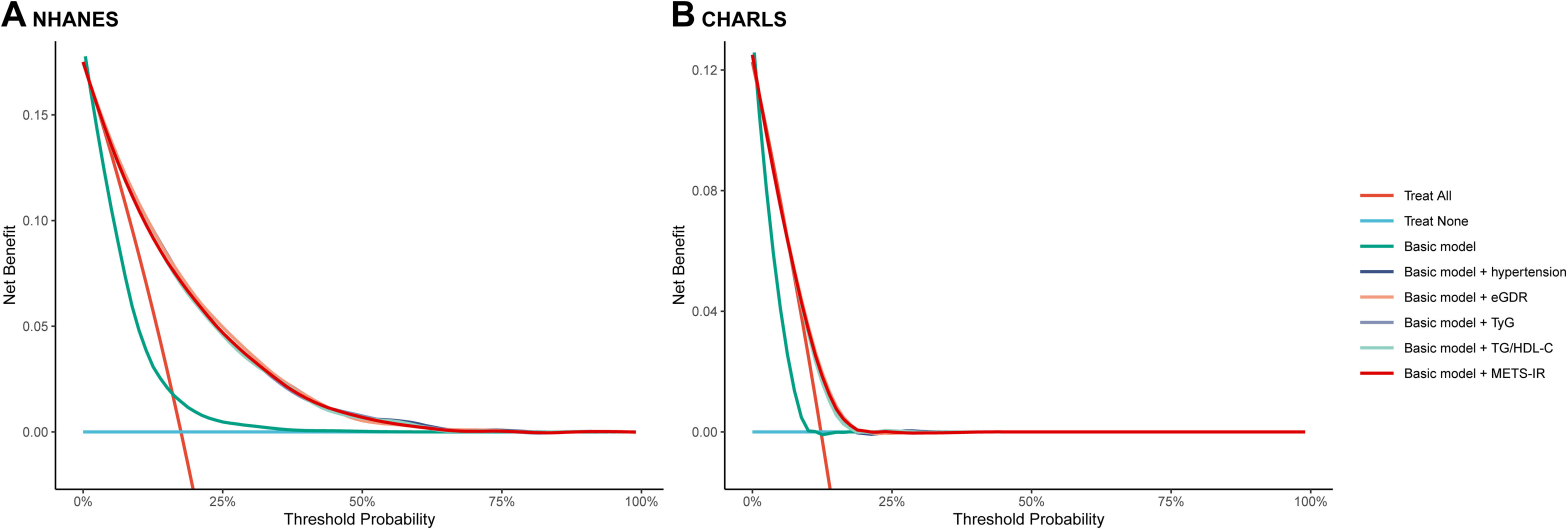
The decision curve analysis of eGDR, TyG, TG/HDL-C and METS-IR to compare the clinical utility for cardiovascular disease, the y-axis represents net benefits, calculated by subtracting the relative harm (false positives) from the benefits (true positives). The x-axis calculates the threshold probability. **(A)** NHANES, **(B)** CHARLS. The basic model adjusted age, sex, marital status, education, smoking, alcohol consumption status, total cholesterol, high density lipoprotein cholesterol, triglyceride, low density lipoprotein cholesterol, blood urea nitrogen, uric acid, hemoglobin, and obesity. eGDR, estimated glucose disposal rate; TyG, triglyceride glucose; HDL-C, high-density lipoprotein cholesterol; METS-IR, metabolic score for insulin resistance.

In this study, the basic models were established to include age, sex, rural residence, marital status, education, smoking status, alcohol consumption status, TC, HDL-C, LDL-C, TG, LDL, BUN, UA, hemoglobin, and obesity. Incorporating eGDR, TyG, TG/HDL-C, and METS-IR significantly improved the predictive performance of the basic model for CVD in both NHANES and CHARLS (**Table 3**; **Figures 3A, B**). The decision curve analysis further confirmed the clinical relevance of these additions (**Figures 4A, B**). According to the results, eGDR outperformed TyG, TG/HDL-C, and METS-IR in predicting both current and future CVD. Notably, the area under the curve (AUC) for predicting current CVD was higher than for predicting future CVD. Incorporating eGDR, TyG, TG/HDL-C, and METS-IR significantly improved the predictive ability of the basic model for heart disease in both NHANES and CHARLS (**Supplementary Table S4**; **Supplementary Figure S10**). The decision curve analysis further confirmed the clinical relevance of these additions (**Supplementary Figure S11**). For stroke, only the addition of eGDR significantly improved the predictive performance of the basic model for current stroke risk in NHANES (**Supplementary Table S5**; **Supplementary Figure S12**). The decision curve analysis confirmed its clinical relevance (**Supplementary Figure S13**). Similar findings indicated that the eGDR index outperformed other indices, with the AUC for predicting heart disease or stroke being higher than for predicting their incidence. Additionally, all net NRI and IDI metrics for CVD and heart disease were significant (P < 0.001) (**Table 3**; **Supplementary Table S4**), and most NRI and IDI metrics for stroke were also significant (**Supplementary Table S5**). Although combined hypertension and the basic model improved the predictive performance for all endpoints, it was still less effective compared to the inclusion of eGDR.

**Table 3:** Improvement in discrimination and risk reclassification for cardiovascular diseases after adding eGDR, TyG, TG/HDL-C or METS-IR.

Model	AUC (95% CI)	P value	Youden’s index	NRI (95% CI)	P value	IDI (95% CI)	P value
NAHNES							
Basic model	0.742 (0.727, 0.742)	Ref	0.3668	Ref	Ref	Ref	Ref
+ hypertension	0.779 (0.766, 0.779)	<0.001	0.4281	0.053 (0.039, 0.066)	<0.001	0.124 (0.115, 0.132)	<0.001
+ eGDR	0.782 (0.769, 0.782)	<0.001	0.4283	0.062 (0.048, 0.076)	<0.001	0.127 (0.118, 0.136)	<0.001
+ TyG	0.769 (0.755, 0.769)	<0.001	0.4084	0.012 (0.004, 0.020)	0.004	0.116 (0.108, 0.125)	<0.001
+ TG/HDL-C	0.768 (0.754, 0.768)	<0.001	0.4070	0.008 (-0.001, 0.016)	0.099	0.115 (0.106, 0.123)	<0.001
+ METS-IR	0.771 (0.757, 0.771)	<0.001	0.4109	0.016 (0.007, 0.024)	<0.001	0.117 (0.109, 0.126)	<0.001
CHARLS							
Basic model	0.582 (0.561, 0.582)	Ref	0.1375	Ref	Ref	Ref	Ref
+ hypertension	0.612 (0.591, 0.612)	<0.001	0.1823	0.028 (0.008, 0.047)	0.0064	0.010 (0.007, 0.012)	<0.001
+ eGDR	0.616 (0.595, 0.616)	<0.001	0.1865	0.038 (0.017, 0.059)	<0.001	0.011 (0.008, 0.013)	<0.001
+ TyG	0.602 (0.582, 0.602)	0.001	0.1536	0.032 (0.013, 0.050)	<0.001	0.008 (0.006, 0.010)	<0.001
+ TG/HDL-C	0.598 (0.578, 0.598)	0.004	0.1475	0.028 (0.010, 0.046)	0.002	0.007 (0.005, 0.009)	<0.001
+ METS-IR	0.608 (0.588, 0.608)	<0.001	0.1698	0.037 (0.018, 0.057)	<0.001	0.009 (0.007, 0.011)	<0.001

The basic model included age, sex, marital status, education, smoking, alcohol consumption status, total cholesterol, high density lipoprotein cholesterol, triglyceride, low density lipoprotein cholesterol, blood urea nitrogen, uric acid, hemoglobin, and obesity.

AUC, area under curve; CHARLS, China Health and Retirement Longitudinal Study; CI, confidence interval; eGDR, estimated glucose disposal rate; NRI, net reclassification improvement; Ref, reference; IDI, integrated discrimination improvement; TyG, triglyceride glucose; TG, triglyceride; HDL-C, high-density lipoprotein cholesterol; METS-IR, metabolic score for insulin resistance; NHANES, National Health and Nutrition Examination Survey.

## Discussion

Our study is the first to examine the predictive performance of eGDR, TyG, TG/HDL-C, and METS-IR for the current or future incidence of CVD among middle-aged and elderly participants. We utilized two nationwide prospective cohort studies: NHANES to assess the association between these indices and current CVD and CHARLS to evaluate their impact on the incidence of CVD. Our findings offer a nuanced understanding of how these factors interact with and influence CVD, and provide valuable insights for clinical practice and prevention. In this study, we have summarized several key findings. First, a lower eGDR was significantly associated with a higher incidence and risk of CVD both currently and in the future. Higher TyG and METS-IR levels were also significantly associated with an increased incidence or risk of CVD in both current and future contexts. In contrast, the TG/HDL-C ratio was not significantly associated to CVD, heart disease, or stroke. Second, subgroup analysis revealed no significant interactions between the quartiles of eGDR, TyG, TG/HDL-C, or METS-IR and the current or feature incidence of various endpoints in most subgroups. Third, the NHANES results revealed a nonlinear association between eGDR, TyG, and METS-IR and current CVD. In contrast, the CHARLS results showed a linear association between these indices and the feature incidence, which remained consistent even after full adjustment. Finally, the incorporation of eGDR, TyG, TG/HDL-C, and METS-IR significantly improved the predictive power of the basic model for current and future CVD. This study highlighted the superior efficacy of adding IR indices to a basic model. Furthermore, the eGDR index is the most promising indicator for preventing and assessing the risk and incidence of CVD among middle-aged and elderly participants, outperforming the basic model alone and the basic model plus hypertension, TyG, TG/HDL-C, and METS-IR.

This study is the first to examine the impact of eGDR, TyG level, TG/HDL-C ratio, and METS-IR on CVD and its incidence in middle-aged and elderly participants. IR is associated with diabetes, dysregulated lipid metabolism, and increased BP, which are all major risk factors for incident CVD (9, 37). The eGDR is a reliable surrogate marker of IR and an effective predictor of future CVD (18, 29), and is also associated with increased risks of all-cause and CVD mortality (38). Participants with lower eGDR had a higher risk of future CVD. Combining the eGDR with the basic model significantly improved its predictive value for current and feature CVD. This is the first study to evaluate the predictive value of eGDR for current and feature CVD. Our study found that participants with a lower eGDR had a higher current or feature incidence of CVD. Additionally, the eGDR emerged as the most promising index for predicting and preventing CVD risk and incidence among middle-aged and elderly individuals in both current and future assessments.

TyG levels show a strong relationship with HOMA-IR and are recognized as an effective method for evaluating IR in hypertensive participants (39). Previous studies have demonstrated that TyG is a reliable surrogate marker and predictor of IR (40–42). Some studies have proposed that TyG plays an important role in the exacerbation of IR through its underlying biological mechanisms. For example, pathological conditions such as hyperlipidemia and/or hyperglycemia can increase the formation of advanced glycation end products (AGEs) (43). The buildup of AGEs in metabolic organs can trigger oxidative stress and inflammation and exacerbate IR (44, 45). Adipose tissue is a major source of oxidative stress (46). Enlarged adipose tissue can release harmful lipids that disrupt insulin sensitivity and exacerbate IR (47).

In this study, the results indicated that the TG/HDL-C ratio was not significantly associated with CVD, heart disease, or stroke, either in terms of risk or incidence. However, these results contrast with those of some previously published studies (48, 49). In contrast, our study found similar results and revealed that the TG/HDL-C ratios was not associated with stroke (48). We cannot eliminate the possibility that the limited power may have affected our ability to identify such an association. Few studies have investigated the relationship between the TG/HDL-C ratio and CVD risk.

METS-IR, derived from traditional clinical examination indices such as FBG, TG, HDL-C, and BMI, is utilized to recognize the signs of IR and metabolic disorders (50). As serum insulin is not routinely evaluated in clinical examinations, METS-IR offers a more accessible alternative to insulin-based indices. Previous studies have explored its predictive role for the development of cardiovascular events in specific populations (51–53). A previously published study involving 6,489 Chinese participants found that METS-IR was a strong predictor of incident chronic heart disease, particularly in females (53). Similarly, a Korean community study involving 17,943 non-diabetic individuals showed that a higher METS-IR score is associated with a higher risk of ischemic heart disease (52). A previously published study involving 2,031 participants from the Urumqi research on sleep apnea and hypertension found that METS-IR is an important predictor of CVD in individuals with hypertension (54). Similarly, another study showed a significant association between METS-IR and stroke risk in 14,032 patients with hypertension (55). However, our study revealed a significant association between the METS-IR and the stroke risk. However, it is also associated with a future incidence of stroke in middle-aged and elderly individuals.

Previous studies have indicated that HOMA-IR is a reliable surrogate marker for IR and is associated with the risk of incident CVD (56, 57). These results revealed that IR indices could be novel and prospective biomarkers for predicting the risk of CVD. However, the use of HOMA-IR as a measure is limited because it relies on FBG and insulin levels, which are not general clinical examinations in nondiabetic patients without diabetes. Additionally, factors such as the use of insulin, insulin sensitizers, and insulin secretagogues can interfere with HOMA-IR measurements, potentially leading to misclassification (58, 59).

Interestingly, IR contributes to endothelial dysfunction, arterial stiffness, and an imbalanced sympathetic nervous system, all of which significantly influence CVD development. These issues can lead to frailty, a common condition in patients with CVD. Frail or pre-frail individuals should be the primary focus for preventing adverse CVD outcomes. Changes in frailty status are linked to varying risks of developing CVD; worsening frailty increases these risks, whereas improvement decreases them. Hence, the relationship between CVD and frailty should be carefully monitored (60–62).

As expected, our findings demonstrated that the eGDR had a markedly superior predictive performance for incident CVD, both currently and in the future, compared to TyG, TG/HDL-C, and METS-IR indices in middle-aged and elderly individuals. This superior performance may be attributed to the integration of clinical and laboratory data into eGDR, which provides a more comprehensive evaluation of IR. Unlike traditional methods, which can be invasive and costly, the eGDR is calculated using only WC, HbA1c, and the presence of hypertension, making it more suitable for large-scale clinical applications. Moreover, the eGDR has comparable accuracy to the HIEG clamp in evaluating IR (36, 63) and demonstrates a strong predictive ability for CVD incidence. The attributable risk of CVD explained by the eGDR may be at least partially due to each component in its formula. The importance of our results lies in the fact that the eGDR, which is readily calculated in everyday clinical practice, might help distinguish patients at high risk of subclinical alterations, which may in turn predict future complications. These insights could inform preventive measures and help reduce the risk of CVD in middle-aged and elderly individuals. Future research should delve into understanding its pathophysiological mechanisms and develop intervention strategies based on the eGDR index, provide new insights and strategies to enhance the outlook for CVD, and adopt alternative approaches such as time-varying covariate models to provide a more comprehensive understanding of the relationship between the eGDR index and CVD.

### Strengths and limitations

This study had several strengths First, this study was the first study to assess the predictive performance of eGDR, TyG, TG/HDL-C, or METS-IR for current or feature incidence of CVD among middle-aged and elderly participants. Second, our results revealed that the eGDR, TyG, and METS-IR significantly improved the predictive value of the basic models for current or future incidents of CVD, which is expected to inform future CVD predictors. Furthermore, eGDR significantly improved the predictive value of the basic models for incident stroke. Third, both AUCs for predicting CVD were higher than the participants’ CVD incidence. These results reveal the superior value of the combined IR indices compared to that of the basic model. Furthermore, eGDR can be considered the most effective index (vs. basic model, basic model plus hypertension, TyG, TG/HDL-C, and METS-IR) for the current and feature incidences of CVD in middle-aged and elderly individuals.

This study had several limitations should be noted. First, as with any observational study, causality cannot be establish and there is a possibility of reverse causality. However, this limitation appears to have a minimal impact as our analysis included individuals who experienced the endpoint both currently and in the future. Second, although the model included many covariates, residual confounding factors cannot be entirely ruled out. However, thishis is a common challenge in observational cohort studies. Third, CVD in this study was self-reported by the participants based on physician diagnoses, which may have led to recall bias and misclassification. However, this approach is commonly used in cohort studies, and evidence suggests that its impact is minimal (64). Fourth, due to the lack of fasting insulin data, we could not compare the risk of CVD using HOMA-IR with the four proposed IR indices. Finally, the IR indices are known to change dynamically over time. However, owing to cost and other limitations, we could only obtain baseline data for our study.

## Conclusion

Our findings indicate that IR, as evaluated using the eGDR, TyG, and METS-IR indices, is associated with the incidence of CVD, both currently and in the future among middle-aged and elderly individuals. Notably, incorporating eGDR, TyG, and METS-IR into the basic model significantly improved its predictive value for CVD, with eGDR emerging as the most promising index for the prevention and assessment of CVD risk and incidence in this population.

## Data Availability

The datasets presented in this study can be found in online repositories. The names of the repository/repositories and accession number(s) can be found below: The data supporting the findings of this study are available at NHANES (https://wwwn.cdc.gov/Nchs/Nhanes/AnalyticGuidelines.aspx) and CHARLS website (http://charls.pku.edu.cn/en).
