# Supplementary material for: Association between various insulin resistance indices and cardiovascular disease in middle-aged and elderly individuals: evidence from two prospectives nationwide cohort surveys
Source: Front Endocrinol (Lausanne). 2024 Nov 22;15:1483468. doi: 10.3389/fendo.2024.1483468 (PMC11620891; doi:10.3389/fendo.2024.1483468)
Supplement: Supplementary file 1 [file DataSheet1.pdf]

Supplemental materials

Supplemental Tables

Supplementary Table 1 Association of eGDR, TyG, TG/HDL-C, and METS-IR with heart disease.

Supplementary Table 2 Association of eGDR, TyG, TG/HDL-C, and METS-IR with stroke.

Supplementary Table 3 The association of estimated glucose disposal rate with cardiovascular disease, heart disease, and stroke among non-diabetes mellitus participants.

Supplementary Table 4 Improvement in discrimination and risk reclassification for heart disease after adding eGDR, TyG, TG/HDL-C or METS-IR.

Supplementary Table 5 Improvement in discrimination and risk reclassification for stroke after adding eGDR, TyG, TG/HDL-C or METS-IR.

Supplemental Figures

Supplementary Figure 1 Restricted cubic spline curves for cardiovascular diseases, heart disease and stroke according to the eGDR from NHANES and CHARLS cohorts.

Supplementary Figure 2 Restricted cubic spline curves for cardiovascular diseases, heart disease and stroke according to the TyG from NHANES and CHARLS cohorts.

Supplementary Figure 3 Restricted cubic spline curves for cardiovascular diseases, heart disease and stroke according to the TG/HDL-C from NHANES and CHARLS cohorts.

Supplementary Figure 4 Restricted cubic spline curves for cardiovascular diseases, heart disease and stroke according to the METS-IR from NHANES and CHARLS cohorts.

Supplementary Figure 5 Subgroup analysis of the association between (A) eGDR, (B) TyG, (C) TG-HDL, (D) METS-IR and heart disease.

Supplementary Figure 6 Subgroup analysis of the association between (A) eGDR, (B) TyG, (C) TG-HDL, (D) METS-IR and stroke.

Supplementary Figure 7 Subgroup and interaction analyses among the quartile 1−4 and cardiovascular diseases across various subgroups. (A) eGDR, (B) TyG, (C) TG-HDL, (D) METS-IR.

Supplementary Figure 8 Subgroup and interaction analyses among the quartile 1−4 and heart disease across various subgroups. (A) eGDR, (B) TyG, (C) TG-HDL, (D) METS-IR.

Supplementary Figure 9 Subgroup and interaction analyses among the quartile 1−4 and stroke across various subgroups. (A) eGDR, (B) TyG, (C) TG-HDL, (D) METS-IR.

Supplementary Figure 10 The receiver operating characteristic curves of eGDR, TyG, TG/HDL-C and

METS-IR to predict heart disease.

Supplementary Figure 11 The decision curve analysis of eGDR, TyG, TG/HDL-C and METS-IR to compare the clinical utility for heart disease, the y-axis represents net benefits, calculated by subtracting the relative harm (false positives) from the benefits (true positives). The x-axis calculates the threshold probability.

Supplementary Figure 12 The receiver operating characteristic curves of eGDR, TyG, TG/HDL-C and METS-IR to predict stroke.

Supplementary Figure 13 The decision curve analysis of eGDR, TyG, TG/HDL-C and METS-IR to compare the clinical utility for stroke, the y-axis represents net benefits, calculated by subtracting the relative harm (false positives) from the benefits (true positives). The x-axis calculates the threshold probability.

Supplementary Table 1 Association of eGDR, TyG, TG/HDL-C, and METS-IR with heart disease

Index	NHANES						CHARLS					
	Model 1		Model 2		Model 3		Model 1		Model 2		Model 3	
	OR (95% CI)	P value	OR (95% CI)	P value	OR (95% CI)	P value	OR (95% CI)	P value	OR (95% CI)	P value	OR (95% CI)	P value
**eGDR**												
Continues	0.78 (0.76, 0.80)	< 0.001	0.79 (0.77, 0.82)	< 0.001	0.84 (0.81, 0.87)	< 0.001	0.90 (0.87, 0.93)	< 0.001	0.91 (0.88, 0.95)	< 0.001	0.92 (0.88, 0.96)	< 0.001
Quartiles												
Q1	Ref		Ref		Ref		Ref		Ref		Ref	
Q2	0.70 (0.59, 0.82)	< 0.001	0.65 (0.54, 0.77)	< 0.001	0.80 (0.66, 0.98)	0.030	0.73 (0.58, 0.91)	0.007	0.74 (0.59, 0.94)	0.010	0.77 (0.60, 0.97)	0.030
Q3	0.38 (0.32, 0.46)	< 0.001	0.40 (0.33, 0.48)	< 0.001	0.53 (0.42, 0.66)	< 0.001	0.58 (0.45, 0.74)	< 0.001	0.63 (0.49, 0.81)	< 0.001	0.66 (0.51, 0.85)	0.002
Q4	0.16 (0.12, 0.20)	< 0.001	0.21 (0.16, 0.27)	< 0.001	0.33 (0.24, 0.44)	< 0.001	0.56 (0.44, 0.71)	< 0.001	0.60 (0.47, 0.77)	< 0.001	0.64 (0.49, 0.84)	0.001
P for trend		< 0.001		< 0.001		< 0.001		< 0.001		< 0.001		< 0.001
**TyG**												
Continues	1.53 (1.37, 1.71)	< 0.001	1.54 (1.37, 1.72)	< 0.001	1.46 (1.14, 1.87)	0.003	1.18 (1.03, 1.34)	0.013	1.17 (1.02, 1.33)	0.020	1.34 (1.00, 1.79)	0.052
Quartiles												
Q1	Ref		Ref		Ref		Ref		Ref		Ref	
Q2	1.01 (0.83, 1.25)	0.900	0.96 (0.77, 1.19)	0.700	0.95 (0.75, 1.20)	0.670	1.10 (0.85, 1.44)	0.457	1.08 (0.83, 1.41)	0.554	1.07 (0.81, 1.40)	0.646
Q3	1.28 (1.05, 1.56)	0.020	1.21 (0.98, 1.49)	0.070	1.13 (0.88, 1.46)	0.330	1.34 (1.04, 1.72)	0.024	1.28 (0.99, 1.65)	0.061	1.26 (0.94, 1.68)	0.118
Q4	1.85 (1.53, 2.23)	< 0.001	1.78 (1.46, 2.16)	< 0.001	1.44 (1.02, 2.02)	0.040	1.43 (1.12, 1.84)	0.005	1.40 (1.09, 1.80)	0.009	1.47 (1.02, 2.14)	0.042
P for trend		< 0.001		< 0.001		0.040		0.002		0.004		0.031
**TG/HDL-C**												
Continues	1.12 (1.09, 1.15)	< 0.001	1.12 (1.09, 1.16)	< 0.001	1.00 (0.92, 1.09)	0.918	1.00 (0.98, 1.02)	0.670	1.01 (0.98, 1.03)	0.536	0.98 (0.91, 1.04)	0.470
Quartiles												
Q1	Ref		Ref		Ref		Ref		Ref		Ref	
Q2	1.40 (1.14, 1.72)	0.002	1.33 (1.07, 1.65)	0.010	1.21 (0.94, 1.56)	0.150	0.82 (0.63, 1.06)	0.131	0.82 (0.63, 1.06)	0.130	0.78 (0.58, 1.05)	0.103
Q3	1.57 (1.29, 1.93)	< 0.001	1.48 (1.20, 1.83)	< 0.001	1.17 (0.86, 1.60)	0.310	1.22 (0.96, 1.56)	0.098	1.22 (0.96, 1.56)	0.104	1.13 (0.82, 1.56)	0.465
Q4	2.09 (1.72, 2.54)	< 0.001	2.08 (1.69, 2.56)	< 0.001	1.44 (0.92, 2.25)	0.110	1.12 (0.88, 1.44)	0.345	1.14 (0.89, 1.47)	0.283	1.06 (0.68, 1.66)	0.793
P for trend		< 0.001		< 0.001		0.210		0.059		0.045		0.346
**METS-IR**												
Continues	2.31 (1.98, 2.70)	< 0.001	2.72 (2.29, 3.23)	< 0.001	1.99 (1.45, 2.73)	< 0.001	1.39 (1.13, 1.70)	0.002	1.42 (1.16, 1.75)	< 0.001	2.06 (1.34, 3.12)	< 0.001
Quartiles												
Q1	Ref		Ref		Ref		Ref		Ref		Ref	
Q2	1.26 (1.02, 1.56)	0.030	1.28 (1.02, 1.60)	0.030	1.14 (0.89, 1.47)	0.310	1.02 (0.79, 1.34)	0.858	1.04 (0.80, 1.36)	0.775	1.06 (0.80, 1.40)	0.710
Q3	1.64 (1.34, 2.01)	< 0.001	1.66 (1.34, 2.06)	< 0.001	1.25 (0.93, 1.68)	0.140	1.41 (1.10, 1.81)	0.007	1.44 (1.12, 1.86)	0.005	1.50 (1.11, 2.01)	0.008
Q4	2.39 (1.97, 2.91)	< 0.001	2.74 (2.23, 3.38)	< 0.001	1.63 (1.14, 2.32)	0.007	1.44 (1.12, 1.85)	0.004	1.48 (1.15, 1.90)	0.003	1.63 (1.11, 2.38)	0.012
P for trend		< 0.001		< 0.001		0.006		< 0.001		< 0.001		0.0027

Model 1: unadjusted

Model 2: adjusted for age, sex, marital status, education, smoking, and alcohol consumption status

Model 3: model 2 + further adjusted for region, total cholesterol, high-density lipoprotein cholesterol, triglyceride, low-density lipoprotein cholesterol, blood urea nitrogen, uric acid, hemoglobin, and obesity

Abbreviations: CHARLS, China Health and Retirement Longitudinal Study; CI, confidence interval; eGDR, estimated glucose disposal rate; TyG, triglyceride glucose; TG, triglyceride; HDL-C, high-density lipoprotein cholesterol; METS-IR, metabolic score for insulin resistance; NHANES, National Health and Nutrition Examination Survey; OR, odd ratio.

Supplementary Table 2 Association of eGDR, TyG, TG/HDL-C, and METS-IR with stroke

Index	NHANES						CHARLS					
	Model 1		Model 2		Model 3		Model 1		Model 2		Model 3	
	OR (95% CI)	P value	OR (95% CI)	P value	OR (95% CI)	P value	OR (95% CI)	P value	OR (95% CI)	P value	OR (95% CI)	P value
**eGDR**												
Continues	0.83 (0.79, 0.86)	< 0.001	0.84 (0.81, 0.88)	< 0.001	0.85 (0.81, 0.90)	< 0.001	0.87 (0.82, 0.92)	< 0.001	0.88 (0.83, 0.93)	< 0.001	0.91 (0.86, 0.97)	0.003
Quartiles												
Q1	Ref		Ref		Ref		Ref		Ref		Ref	
Q2	0.77 (0.60, 0.98)	0.032	0.74 (0.58, 0.95)	0.020	0.77 (0.58, 1.02)	0.071	0.68 (0.49, 0.92)	0.014	0.69 (0.50, 0.94)	0.019	0.76 (0.55, 1.05)	0.100
Q3	0.54 (0.41, 0.70)	< 0.001	0.57 (0.43, 0.74)	< 0.001	0.59 (0.43, 0.80)	< 0.001	0.58 (0.42, 0.80)	0.001	0.62 (0.44, 0.86)	0.005	0.73 (0.51, 1.04)	0.081
Q4	0.19 (0.13, 0.27)	< 0.001	0.25 (0.17, 0.36)	< 0.001	0.27 (0.17, 0.42)	< 0.001	0.43 (0.30, 0.61)	< 0.001	0.45 (0.31, 0.64)	< 0.001	0.58 (0.39, 0.85)	0.006
P for trend		< 0.001		< 0.001		< 0.001		< 0.001		< 0.001		0.006
**TyG**												
Continues	1.06 (0.90, 1.25)	0.450	1.00 (0.85, 1.19)	0.960	1.15 (0.80, 1.64)	0.430	1.48 (1.24, 1.74)	< 0.001	1.50 (1.26, 1.78)	< 0.001	1.42 (0.98, 2.07)	0.067
Quartiles												
Q1	Ref		Ref		Ref		Ref		Ref		Ref	
Q2	0.85 (0.64, 1.13)	0.250	0.81 (0.61, 1.08)	0.150	0.85 (0.62, 1.16)	0.310	1.66 (1.13, 2.49)	0.012	1.66 (1.13, 2.49)	0.012	1.43 (0.96, 2.16)	0.085
Q3	0.87 (0.65, 1.15)	0.320	0.80 (0.60, 1.07)	0.140	0.88 (0.62, 1.27)	0.500	2.09 (1.43, 3.08)	< 0.001	2.08 (1.42, 3.07)	< 0.001	1.54 (1.02, 2.36)	0.043
Q4	1.07 (0.82, 1.40)	0.630	0.96 (0.73, 1.27)	0.780	1.09 (0.66, 1.76)	0.740	2.13 (1.47, 3.14)	< 0.001	2.19 (1.51, 3.24)	< 0.001	1.31 (0.79, 2.18)	0.303
P for trend		0.600		0.810		0.950		< 0.001		< 0.001		0.274
**TG-HDL**												
Continues	1.01 (0.96, 1.06)	0.650	1.00 (0.95, 1.05)	0.960	1.03 (0.90, 1.14)	0.580	1.03 (1.01, 1.05)	0.008	1.03 (1.01, 1.05)	0.004	0.96 (0.88, 1.03)	0.294
Quartiles												
Q1	Ref		Ref		Ref		Ref		Ref		Ref	
Q2	1.00 (0.75, 1.33)	0.990	0.93 (0.70, 1.25)	0.640	1.09 (0.77, 1.55)	0.620	1.81 (1.23, 2.71)	0.003	1.84 (1.25, 2.75)	0.003	1.55 (1.01, 2.41)	0.048
Q3	0.92 (0.69, 1.23)	0.560	0.82 (0.61, 1.11)	0.200	1.06 (0.68, 1.66)	0.790	1.97 (1.35, 2.93)	< 0.001	2.00 (1.36, 2.98)	< 0.001	1.46 (0.90, 2.39)	0.131
Q4	1.18 (0.90, 1.55)	0.230	1.09 (0.82, 1.45)	0.540	1.87 (0.99, 3.51)	0.050	2.27 (1.56, 3.36)	< 0.001	2.34 (1.61, 3.47)	< 0.001	1.40 (0.76, 2.61)	0.282
P for trend		0.330		0.700		0.190		< 0.001		< 0.001		0.455
**METS-IR**												
Continues	1.37 (1.08, 1.73)	0.008	1.40 (1.10, 1.78)	0.007	1.48 (0.94, 2.30)	0.090	1.95 (1.49, 2.54)	< 0.001	2.07 (1.58, 2.69)	< 0.001	2.08 (1.17, 3.62)	0.011
Quartiles												
Q1	Ref		Ref		Ref		Ref		Ref		Ref	
Q2	0.90 (0.67, 1.21)	0.500	0.91 (0.67, 1.23)	0.540	0.87 (0.62, 1.23)	0.430	1.89 (1.26, 2.88)	0.002	2.00 (1.33, 3.06)	0.001	1.80 (1.18, 2.79)	0.007
Q3	0.97 (0.72, 1.29)	0.830	0.94 (0.70, 1.26)	0.670	0.83 (0.55, 1.24)	0.360	2.40 (1.63, 3.61)	< 0.001	2.58 (1.74, 3.89)	< 0.001	2.09 (1.34, 3.32)	0.001
Q4	1.32 (1.00, 1.73)	0.05	1.33 (1.01, 1.77)	0.040	1.12 (0.69, 1.83)	0.650	2.58 (1.75, 3.86)	< 0.001	2.79 (1.89, 4.20)	< 0.001	1.91 (1.11, 3.31)	0.021
P for trend		0.04		0.040		0.590		< 0.001		< 0.001		0.021

Model 1: unadjusted

Model 2: adjusted for age, sex, marital status, education, smoking, and alcohol consumption status

Model 3: model 2 + further adjusted for region, total cholesterol, high-density lipoprotein cholesterol, triglyceride, low-density lipoprotein cholesterol, blood urea nitrogen, uric acid, hemoglobin, and obesity

Abbreviations: CHARLS, China Health and Retirement Longitudinal Study; CI, confidence interval; eGDR, estimated glucose disposal rate; TyG, triglyceride glucose; TG, triglyceride; HDL-C, high-density lipoprotein cholesterol; METS-IR, metabolic score for insulin resistance; NHANES, National Health and Nutrition Examination Survey; OR, odd ratio.

Supplementary Table 3 The association of estimated glucose disposal rate with cardiovascular diseases among non-diabetes mellitus participants

Index	NHANES						CHARLS					
	Cardiovascular diseases		Heart disease		Stroke		Cardiovascular diseases		Heart disease		Stroke	
	OR (95% CI)	P value	OR (95% CI)	P value	OR (95% CI)	P value	OR (95% CI)	P value	OR (95% CI)	P value	OR (95% CI)	P value
**eGDR**												
Continues	0.82 (0.78, 0.86)	< 0.001	0.83 (0.78, 0.87)	< 0.001	0.82 (0.76, 0.89)	< 0.001	0.91 (0.87, 0.95)	< 0.001	0.93 (0.89, 0.98)	0.004	0.91 (0.85, 0.97)	0.007
Quartiles												
Q1	Ref		Ref		Ref		Ref		Ref		Ref	
Q2	0.77 (0.59, 1.00)	0.054	0.77 (0.59, 1.00)	0.054	0.91 (0.63, 1.32)	0.62	0.69 (0.53, 0.89)	0.005	0.69 (0.53, 0.89)	0.005	0.84 (0.59, 1.20)	0.344
Q3	0.48 (0.36, 0.64)	< 0.001	0.48 (0.36, 0.64)	< 0.001	0.48 (0.32, 0.72)	< 0.001	0.70 (0.53, 0.93)	0.013	0.70 (0.53, 0.93)	0.013	0.84 (0.57, 1.24)	0.386
Q4	0.31 (0.21, 0.46)	< 0.001	0.31 (0.21, 0.46)	< 0.001	0.33 (0.19, 0.56)	< 0.001	0.65 (0.49, 0.87)	0.004	0.65 (0.49, 0.87)	0.004	0.57 (0.36, 0.88)	0.012
P for trend		< 0.001		< 0.001		< 0.001		0.005		0.005		0.02
**TyG**												
Continues	1.36 (0.86, 2.18)	0.1957	1.85 (1.10, 3.16)	0.0223	0.65 (0.33, 1.32)	0.2202	1.26 (0.86, 1.89)	0.2452	1.27 (0.81, 2.05)	0.303	1.30 (0.70, 2.51)	0.4208
Quartiles												
Q1	Ref		Ref		Ref		Ref		Ref		Ref	
Q2	0.99 (0.74, 1.34)	0.958	0.99 (0.74, 1.34)	0.958	0.87 (0.59, 1.29)	0.481	0.92 (0.69, 1.24)	0.602	0.92 (0.69, 1.24)	0.602	1.20 (0.78, 1.87)	0.403
Q3	1.36 (0.97, 1.91)	0.077	1.36 (0.97, 1.91)	0.077	0.94 (0.58, 1.54)	0.818	1.14 (0.83, 1.57)	0.424	1.14 (0.83, 1.57)	0.424	1.19 (0.76, 1.89)	0.454
Q4	1.35 (0.81, 2.25)	0.252	1.35 (0.81, 2.25)	0.252	1.22 (0.57, 2.60)	0.609	1.26 (0.81, 1.97)	0.303	1.26 (0.81, 1.97)	0.303	0.80 (0.44, 1.47)	0.472
P for trend		0.09		0.09		0.907		0.236		0.236		0.664
**TG/HDL-C**												
Continues	1.01 (0.89, 1.15)	0.847	0.98 (0.85, 1.12)	0.756	1.07 (0.87, 1.28)	0.48	0.96 (0.87, 1.05)	0.416	0.95 (0.84, 1.05)	0.4051	0.98 (0.85, 1.10)	0.772
Quartiles												
Q1	Ref		Ref		Ref		Ref		Ref		Ref	
Q2	1.29 (0.93, 1.79)	0.129	1.29 (0.93, 1.79)	0.129	0.97 (0.63, 1.49)	0.872	0.75 (0.54, 1.03)	0.073	0.75 (0.54, 1.03)	0.073	1.22 (0.76, 1.96)	0.412
Q3	1.26 (0.85, 1.88)	0.252	1.26 (0.85, 1.88)	0.252	0.84 (0.48, 1.46)	0.534	1.07 (0.75, 1.55)	0.703	1.07 (0.75, 1.55)	0.703	1.08 (0.64, 1.86)	0.778
Q4	1.57 (0.89, 2.76)	0.118	1.57 (0.89, 2.76)	0.118	1.50 (0.68, 3.30)	0.316	0.98 (0.59, 1.64)	0.931	0.98 (0.59, 1.64)	0.931	0.88 (0.44, 1.82)	0.734
P for trend		0.197		0.197		0.75		0.6		0.6		0.652
**METS-IR**												
Continues	2.15 (1.20, 3.85)	0.01	2.65 (1.38, 5.09)	0.003	1.03 (0.42, 2.49)	0.947	2.38 (1.35, 4.21)	0.003	2.11 (1.10, 4.09)	0.026	2.26 (0.90, 5.75)	0.085
Quartiles												
Q1	Ref		Ref		Ref		Ref		Ref		Ref	
Q2	1.39 (1.02, 1.91)	0.038	1.39 (1.02, 1.91)	0.038	1.02 (0.68, 1.54)	0.907	1.00 (0.74, 1.36)	0.999	1.00 (0.74, 1.36)	0.9995	2.35 (1.47, 3.84)	0.0005
Q3	1.29 (0.87, 1.92)	0.214	1.29 (0.87, 1.92)	0.214	0.89 (0.52, 1.53)	0.685	1.34 (0.97, 1.86)	0.08	1.34 (0.97, 1.86)	0.08	1.84 (1.09, 3.17)	0.0254
Q4	2.02 (1.21, 3.38)	0.007	2.02 (1.21, 3.38)	0.007	0.95 (0.46, 1.93)	0.881	1.40 (0.90, 2.18)	0.136	1.40 (0.90, 2.18)	0.1355	2.52 (1.33, 4.86)	0.0051
P for trend		0.02		0.02		0.792		0.0598		0.0598		0.029

Sensitivity analyses were conducted in model 3, adjusted for age, sex, marital status, education, smoking, alcohol consumption status, total cholesterol, high-density lipoprotein

cholesterol, triglyceride, low-density lipoprotein cholesterol, blood urea nitrogen, uric acid, hemoglobin, and obesity.

Abbreviations: CHARLS, China Health and Retirement Longitudinal Study; CI, confidence interval; eGDR, estimated glucose disposal rate; TyG, triglyceride glucose; TG, triglyceride; HDL-C, high-density lipoprotein cholesterol; METS-IR, metabolic score for insulin resistance; NHANES, National Health and Nutrition Examination Survey; OR, odd ratio.

Supplementary Table 4 Improvement in discrimination and risk reclassification for heart disease after adding eGDR, TyG, TG/HDL-C or METS-IR

Model	AUC (95% CI)	P value	NRI (95% CI)	P value	IDI (95% CI)	P value
**NAHNES**						
Basic model	0.736 (0.720, 0.736)	Ref	Ref	Ref	Ref	Ref
+ hypertension	0.787 (0.773, 0.787)	<0.001	0.107 (0.051, 0.162)	<0.001	0.064 (0.052, 0.077)	<0.001
+ eGDR	0.791 (0.776, 0.791)	<0.001	0.130 (0.074, 0.185)	<0.001	0.065 (0.052, 0.077)	<0.001
+ TyG	0.779 (0.764, 0.779)	<0.001	-0.008 (-0.064, 0.048)	0.77	0.055 (0.043, 0.068)	<0.001
+ TG-HDL	0.778 (0.763, 0.778)	<0.001	-1E-04 (-0.053, 0.053)	0.996	0.055 (0.043, 0.067)	<0.001
+ METS-IR	0.781 (0.766, 0.781)	<0.001	-0.014 (-0.072, 0.044)	0.628	0.056 (0.044, 0.069)	<0.001
**CHARLS**						
Basic model	0.556 (0.531, 0.556)	Ref	Ref	Ref	Ref	Ref
+ hypertension	0.609 (0.586, 0.609)	<0.001	0.002 (-0.005, 0.010)	0.507	0.005 (0.003, 0.007)	<0.001
+ eGDR	0.612 (0.588, 0.612)	<0.001	-0.003 (-0.008, 0.003)	0.303	0.005 (0.003, 0.008)	<0.001
+ TyG	0.598 (0.573, 0.598)	<0.001	-9E-04 (-0.007, 0.005)	0.773	0.004 (0.002, 0.006)	<0.001
+ TG-HDL	0.594 (0.571, 0.594)	0.001	0.002 (-0.005, 0.009)	0.538	0.004 (0.002, 0.005)	<0.001
+ METS-IR	0.603 (0.579, 0.603)	<0.001	0.005 (-0.004, 0.013)	0.297	0.005 (0.003, 0.008)	<0.001

The basic model included age, sex, marital status, education, smoking, alcohol consumption status, total cholesterol, high-density lipoprotein cholesterol, triglyceride, low-density lipoprotein cholesterol, blood urea nitrogen, uric acid, hemoglobin, and obesity.

Abbreviations: AUC, area under curve; CHARLS, China Health and Retirement Longitudinal Study; CI, confidence interval; eGDR, estimated glucose disposal rate; NRI, net reclassification improvement; Ref, reference; IDI, integrated discrimination improvement; TyG, triglyceride glucose; TG, triglyceride; HDL-C, high-density lipoprotein cholesterol; METS-IR, metabolic score for insulin resistance; NHANES, National Health and Nutrition Examination Survey.

Supplementary Table 5 Improvement in discrimination and risk reclassification for stroke after adding eGDR, TyG, TG/HDL-C or METS-IR

Model	AUC (95% CI)	P value	NRI (95% CI)	P value	IDI (95% CI)	P value
**NAHNES**						
Basic model	0.730 (0.707, 0.730)	Ref	Ref	Ref	Ref	Ref
+ hypertension	0.739 (0.717, 0.739)	0.202	1.967 (1.960, 1.975)	<0.001	0.031 (0.031, 0.032)	<0.001
+ eGDR	0.746 (0.724, 0.746)	0.001	1.534 (1.507, 1.562)	<0.001	0.022 (0.022, 0.023)	<0.001
+ TyG	0.731 (0.708, 0.731)	0.317	0.200 (0.154, 0.247)	<0.001	3E-04 (2E-04, 4E-04)	<0.001
+ TG-HDL	0.730 (0.707, 0.730)	0.930	-0.041 (-0.087, 0.005)	0.08	1E-04 (0, 1E-04)	0.12
+ METS-IR	0.732 (0.709, 0.732)	0.254	0.235 (0.190, 0.281)	<0.001	0.001 (8E-04, 0.001)	<0.001
**CHARLS**						
Basic model	0.627 (0.595, 0.627)	Ref	Ref	Ref	Ref	Ref
+ hypertension	0.622 (0.589, 0.622)	0.624	0.254 (0.133, 0.374)	<0.001	0.002 (5E-04, 0.002)	0.003
+ eGDR	0.637 (0.604, 0.637)	0.142	0.219 (0.098, 0.339)	<0.001	0.002 (7E-04, 0.003)	0.002
+ TyG	0.632 (0.600, 0.632)	0.291	0.029 (-0.091, 0.150)	0.633	6E-04 (-2E-04, 0.001)	0.160
+ TG-HDL	0.629 (0.597, 0.629)	0.335	0.007 (-0.113, 0.126)	0.914	1E-04 (-3E-04, 6E-04)	0.532
+ METS-IR	0.636 (0.604, 0.636)	0.107	0.077 (-0.044, 0.197)	0.213	0.001 (1E-04, 0.002)	0.039

The basic model included age, sex, marital status, education, smoking, alcohol consumption status, total cholesterol, high-density lipoprotein cholesterol, triglyceride, low-density lipoprotein cholesterol, blood urea nitrogen, uric acid, hemoglobin, and obesity.

Abbreviations: AUC, area under curve; CHARLS, China Health and Retirement Longitudinal Study; CI, confidence interval; eGDR, estimated glucose disposal rate; NRI, net reclassification improvement; Ref, reference; IDI, integrated discrimination improvement; TyG, triglyceride glucose; TG, triglyceride; HDL-C, high-density lipoprotein cholesterol; METS-IR, metabolic score for insulin resistance; NHANES, National Health and Nutrition Examination Survey.

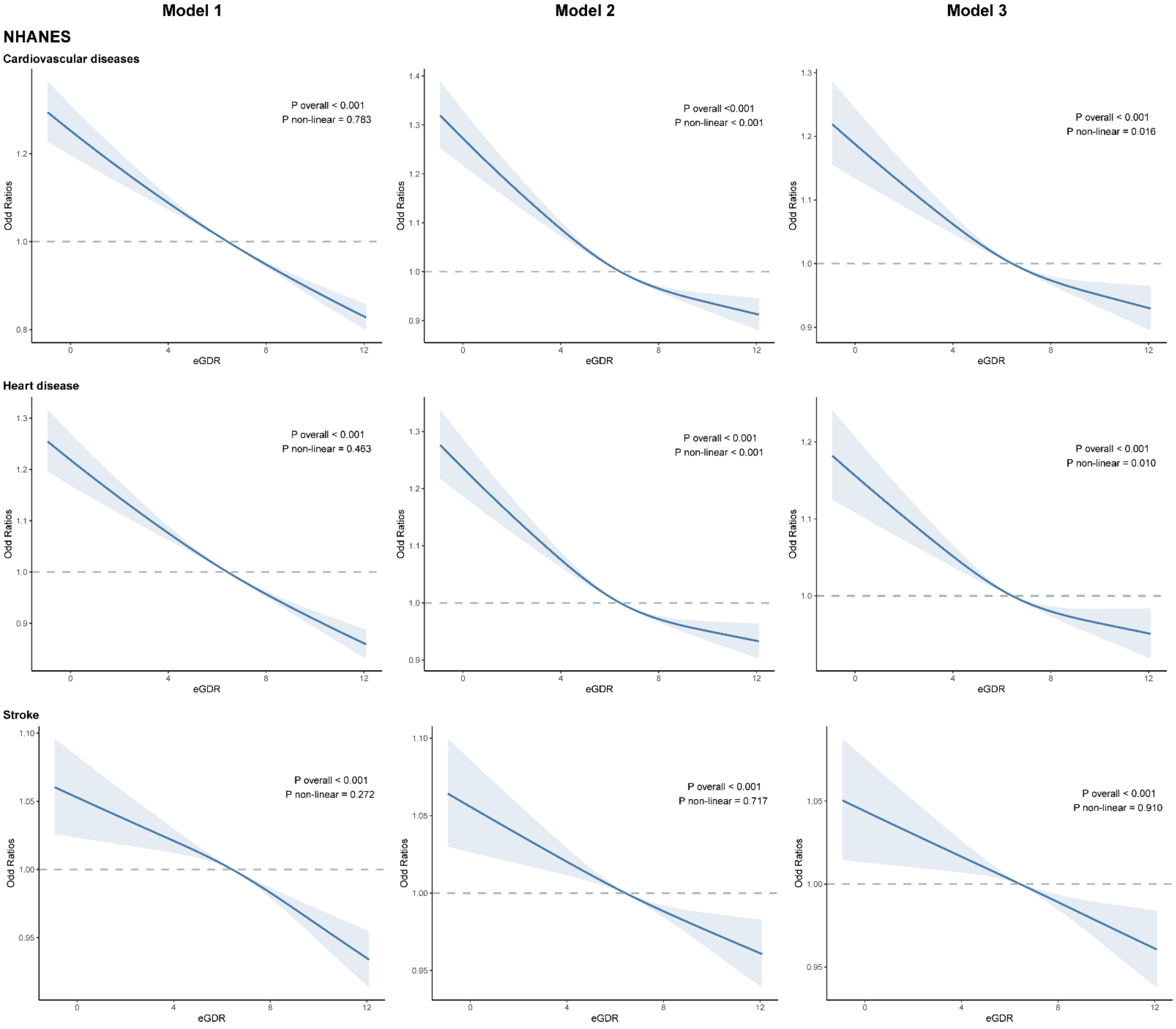
Model 1
Model 2
Model 3
NHANES
Cardiovascular diseases
P overall < 0.001
P non-linear = 0.783
P overall <0.001
P non-linear < 0.001
P overall < 0.001
P non-linear = 0.016
Heart disease
P overall < 0.001
P non-linear = 0.463
P overall < 0.001
P non-linear < 0.001
P overall < 0.001
P non-linear = 0.010
Stroke
P overall < 0.001
P non-linear = 0.272
P overall < 0.001
P non-linear = 0.717
P overall < 0.001
P non-linear = 0.910
Odd Ratios
eGDR

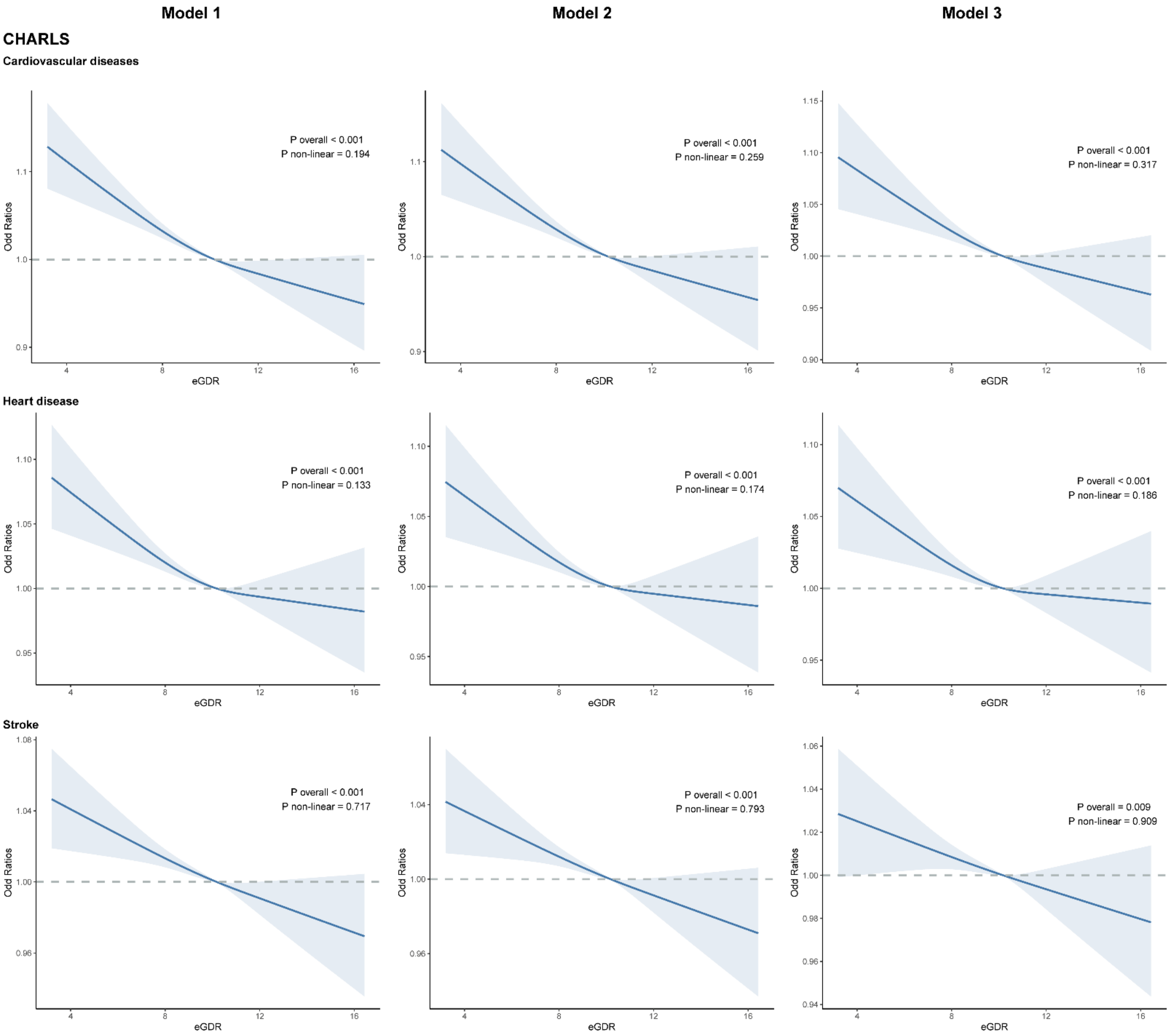

Supplementary Figure 1 Restricted cubic spline curves for cardiovascular diseases, heart disease and stroke according to the eGDR from NHANES and CHARLS cohorts. Odd ratios are indicated by solid lines and 95% CIs by shaded areas. The horizontal dotted line represents the odd ratio of 1.0. Model 1 was an unadjusted model; model 2 adjusted for age, sex, marital status, education, smoking, and alcohol consumption status; model 3 was model 2 + further adjusted region, total cholesterol, high-density lipoprotein cholesterol, triglyceride, low-density lipoprotein cholesterol, blood urea nitrogen, uric acid, hemoglobin, and obesity.

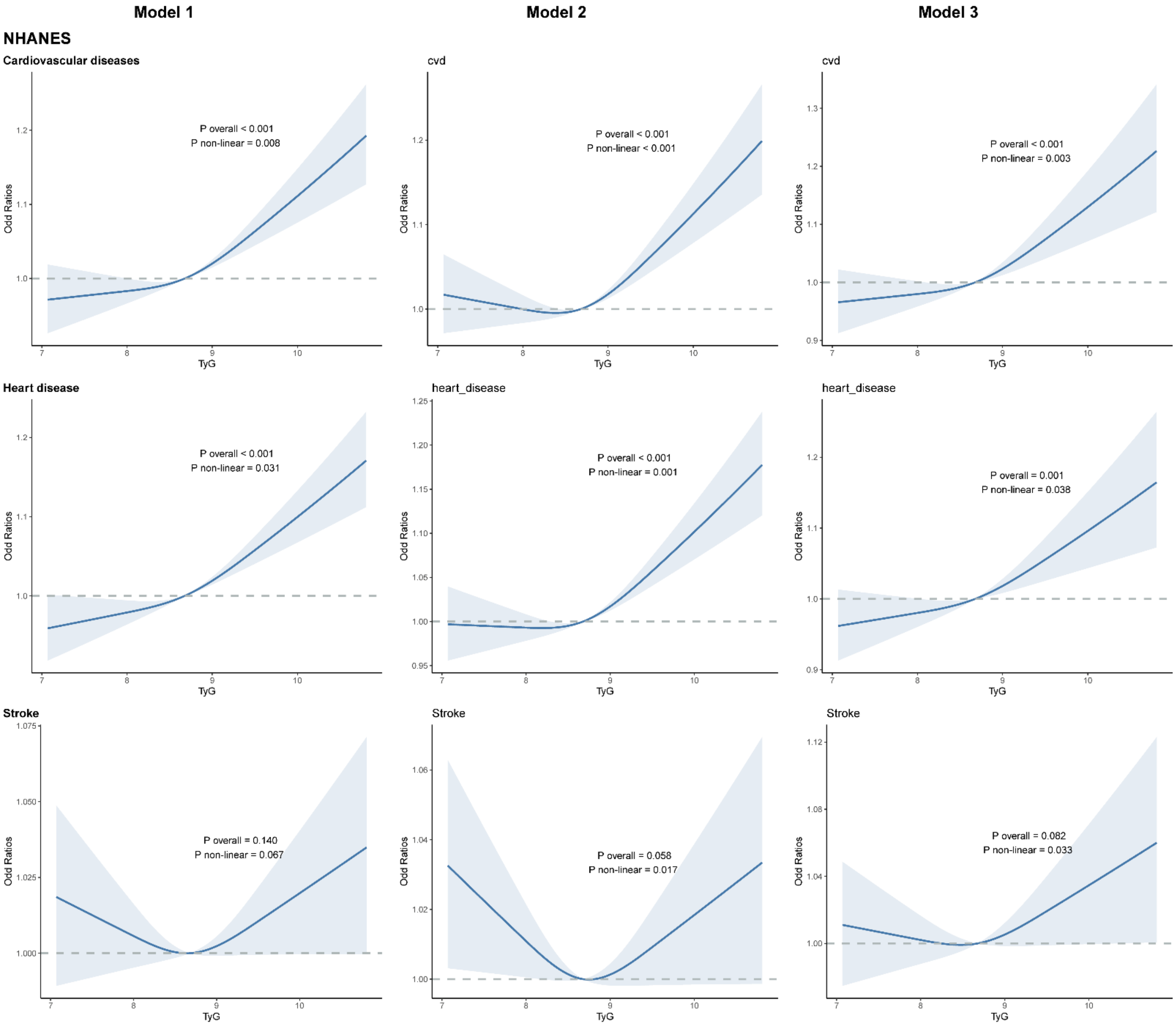
Model 1
Model 2
Model 3
NHANES
Cardiovascular diseases
cvd
cvd
P overall < 0.001
P non-linear = 0.008
P overall < 0.001
P non-linear < 0.001
P overall < 0.001
P non-linear = 0.003
Heart disease
heart_disease
heart_disease
P overall < 0.001
P non-linear = 0.031
P overall < 0.001
P non-linear = 0.001
P overall = 0.001
P non-linear = 0.038
Stroke
Stroke
Stroke
P overall = 0.140
P non-linear = 0.067
P overall = 0.058
P non-linear = 0.017
P overall = 0.082
P non-linear = 0.033
Odd Ratios
TyG

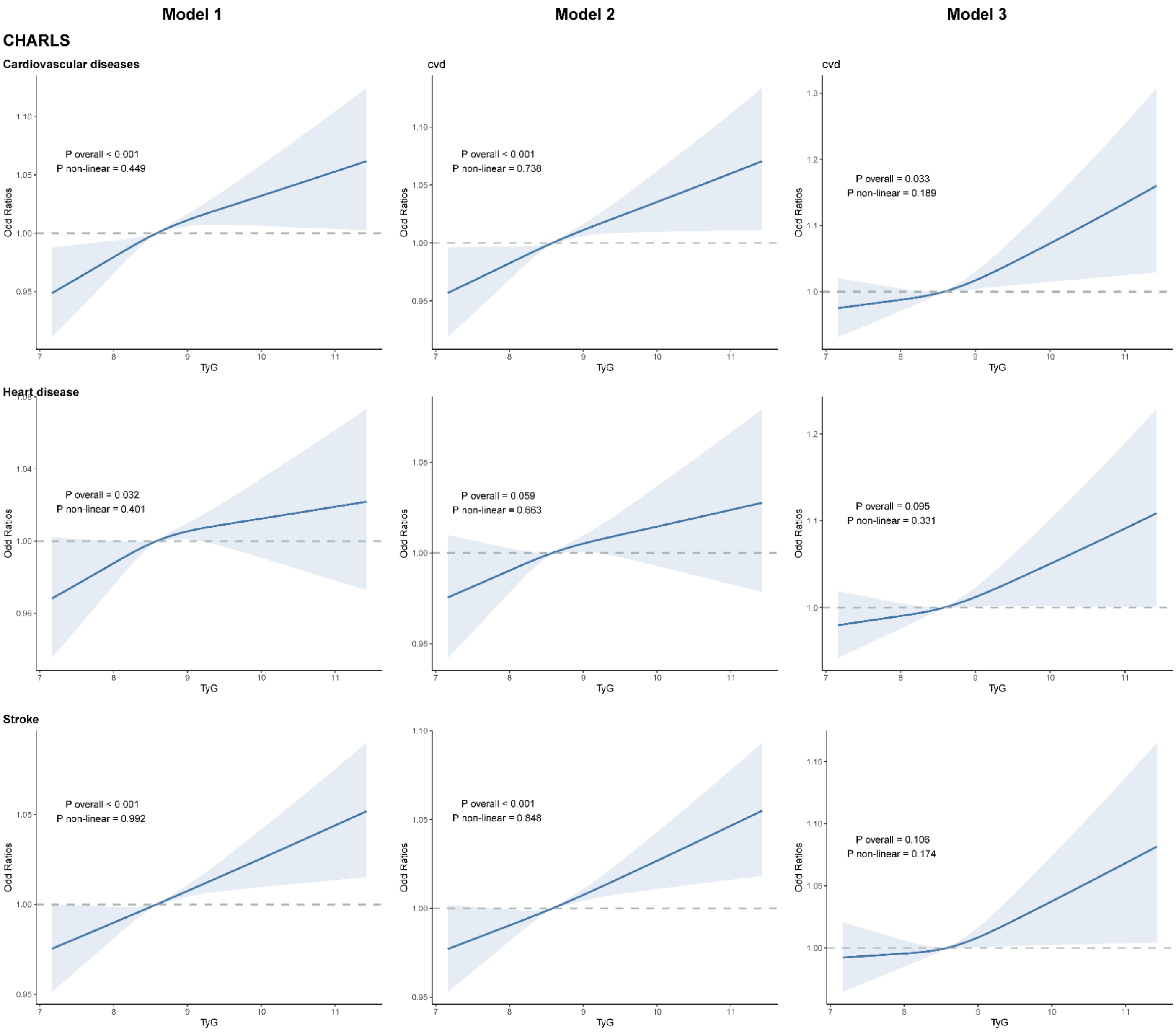

Supplementary Figure 2 Restricted cubic spline curves for cardiovascular diseases, heart disease and stroke according to the TyG from NHANES and CHARLS cohorts. Odd ratios are indicated by solid lines and 95% CIs by shaded areas. The horizontal dotted line represents the odd ratio of 1.0. Model 1 was an unadjusted model; model 2 adjusted for age, sex, marital status, education, smoking, and alcohol consumption status; model 3 was model 2 + further adjusted region, total cholesterol, high-density lipoprotein cholesterol, triglyceride, low-density lipoprotein cholesterol, blood urea nitrogen, uric acid, hemoglobin, and obesity.

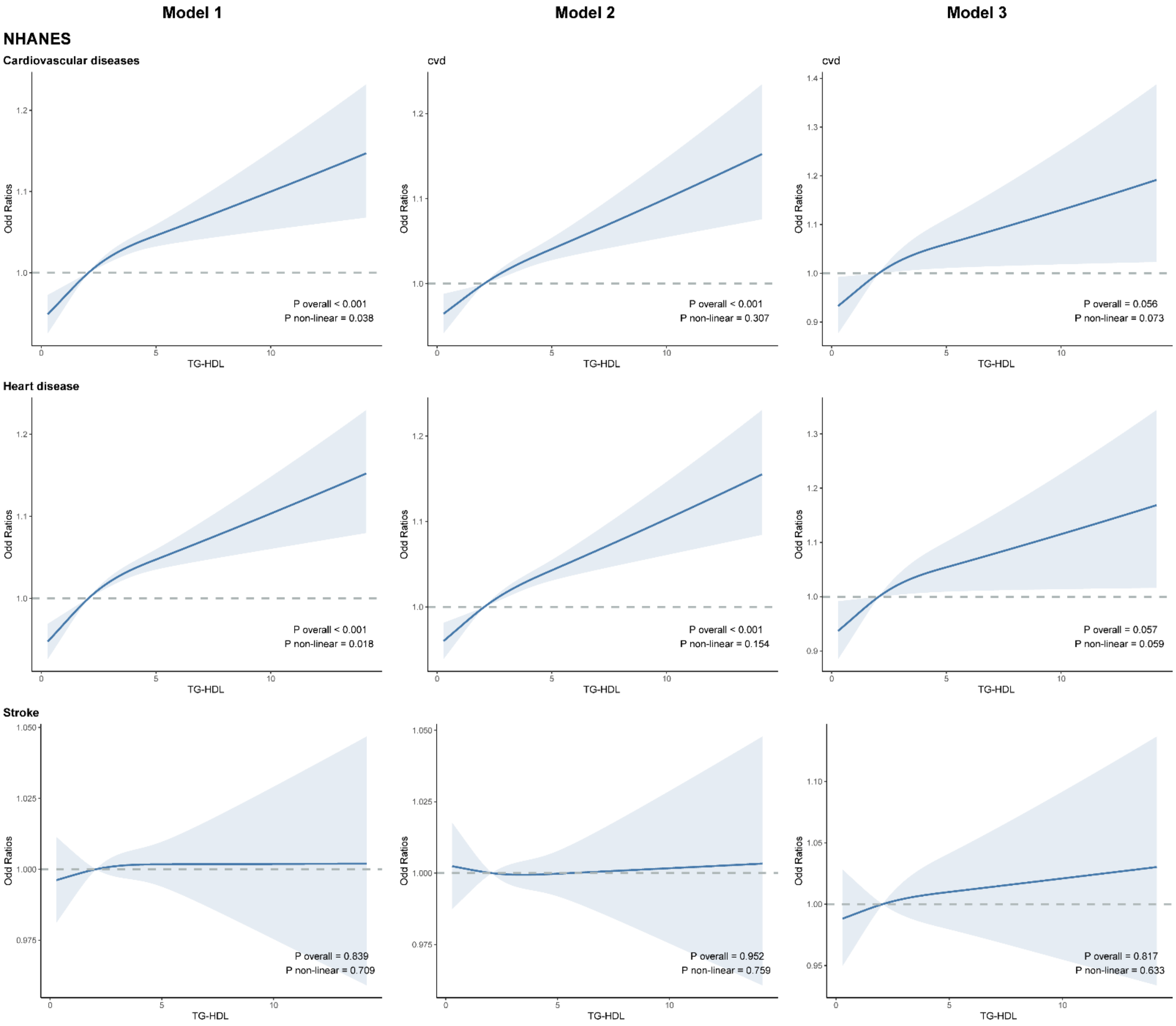
Model 1
Model 2
Model 3
NHANES
Cardiovascular diseases
cvd
cvd
Odd Ratios
TG-HDL
P overall < 0.001
P non-linear = 0.038
P overall < 0.001
P non-linear = 0.307
P overall = 0.056
P non-linear = 0.073
Heart disease
P overall < 0.001
P non-linear = 0.018
P overall < 0.001
P non-linear = 0.154
P overall = 0.057
P non-linear = 0.059
Stroke
P overall = 0.839
P non-linear = 0.709
P overall = 0.952
P non-linear = 0.759
P overall = 0.817
P non-linear = 0.633

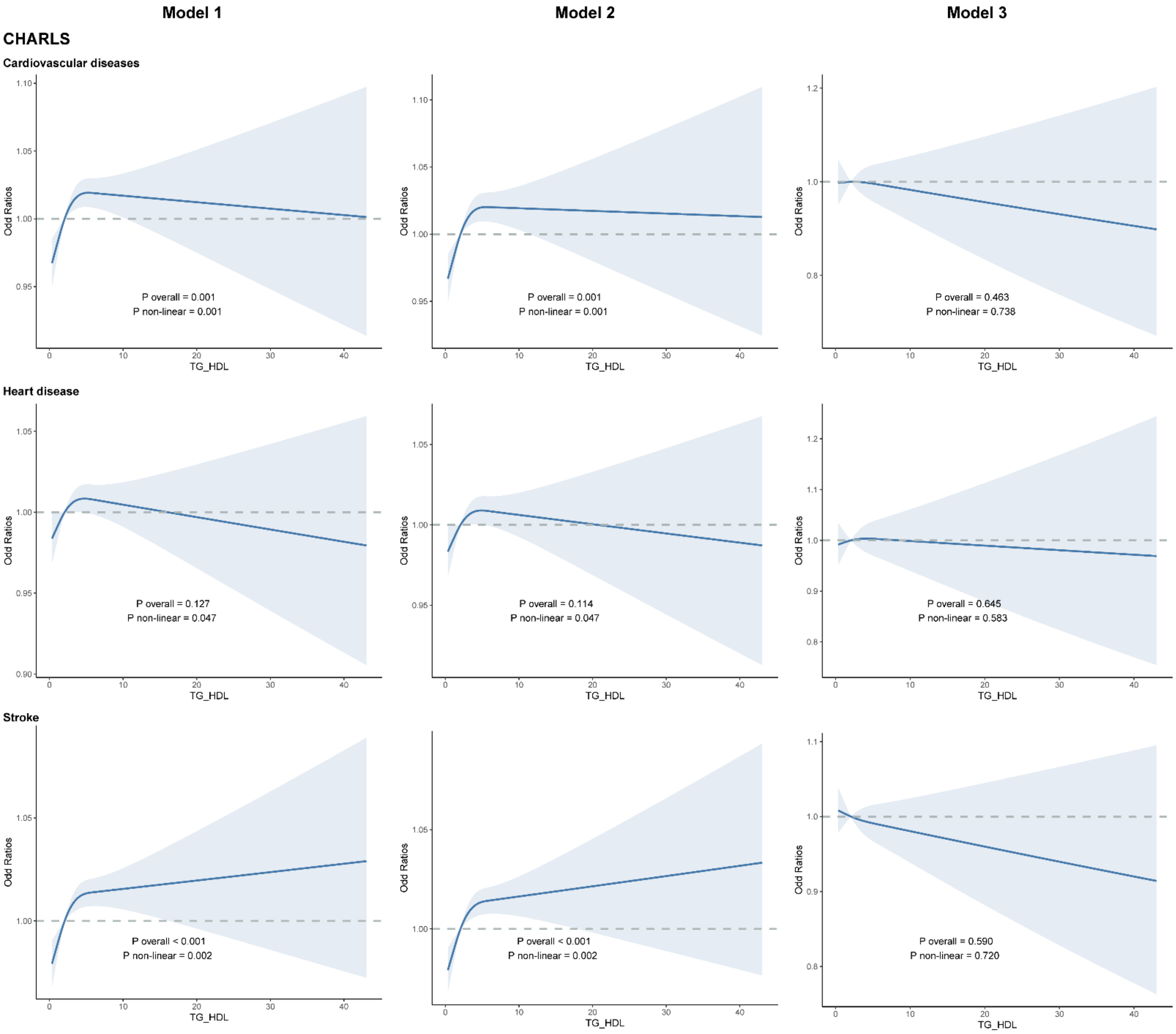

Supplementary Figure 3 Restricted cubic spline curves for cardiovascular diseases, heart disease and stroke according to the TG/HDL-C from NHANES and CHARLS cohorts. Odd ratios are indicated by solid lines and 95% CIs by shaded areas. The horizontal dotted line represents the odd ratio of 1.0. Model 1 was an unadjusted model; model 2 adjusted for age, sex, marital status, education, smoking, and alcohol consumption status; model 3 was model 2 + further adjusted region, total cholesterol, high-density lipoprotein cholesterol, triglyceride, low-density lipoprotein cholesterol, blood urea nitrogen, uric acid, hemoglobin, and obesity.

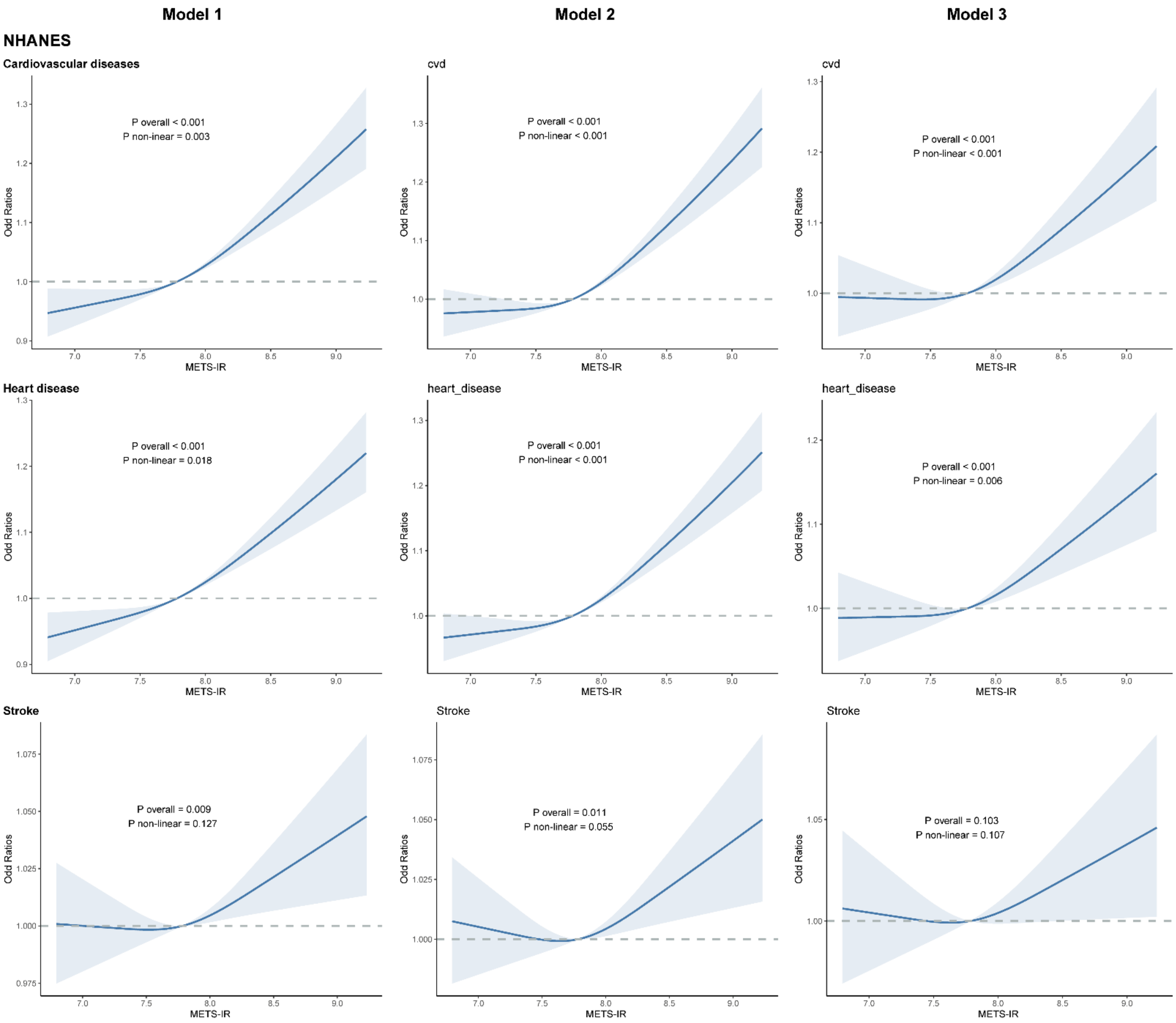

Model 1
Model 2
Model 3
NHANES
Cardiovascular diseases
P overall < 0.001
P non-inear = 0.003
cvd
P overall < 0.001
P non-linear < 0.001
cvd
P overall < 0.001
P non-linear < 0.001
Heart disease
P overall < 0.001
P non-linear = 0.018
heart_disease
P overall < 0.001
P non-linear < 0.001
heart_disease
P overall < 0.001
P non-linear = 0.006
Stroke
P overall = 0.009
P non-linear = 0.127
Stroke
P overall = 0.011
P non-linear = 0.055
Stroke
P overall = 0.103
P non-linear = 0.107
Odd Ratios
METS-IR

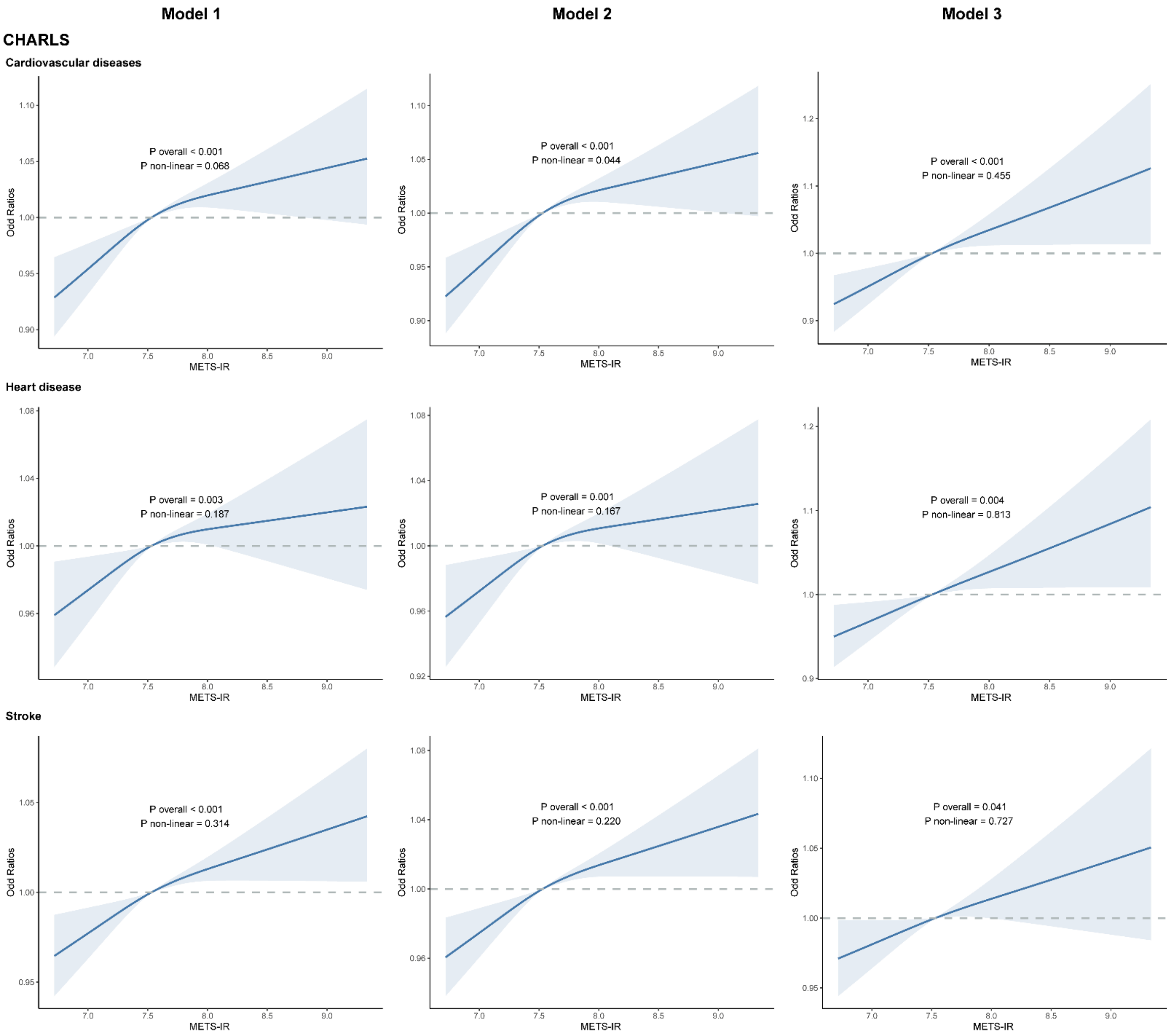

Supplementary Figure 4 Restricted cubic spline curves for cardiovascular diseases, heart disease and stroke according to the METS-IR from NHANES and CHARLS cohorts. Odd ratios are indicated by solid lines and 95% CIs by shaded areas. The horizontal dotted line represents the odd ratio of 1.0. Model 1 was an unadjusted model; model 2 adjusted for age, sex, marital status, education, smoking, and alcohol consumption status; model 3 was model 2 + further adjusted region, total cholesterol, high-density lipoprotein cholesterol, triglyceride, low-density lipoprotein cholesterol, blood urea nitrogen, uric acid, hemoglobin, and obesity.

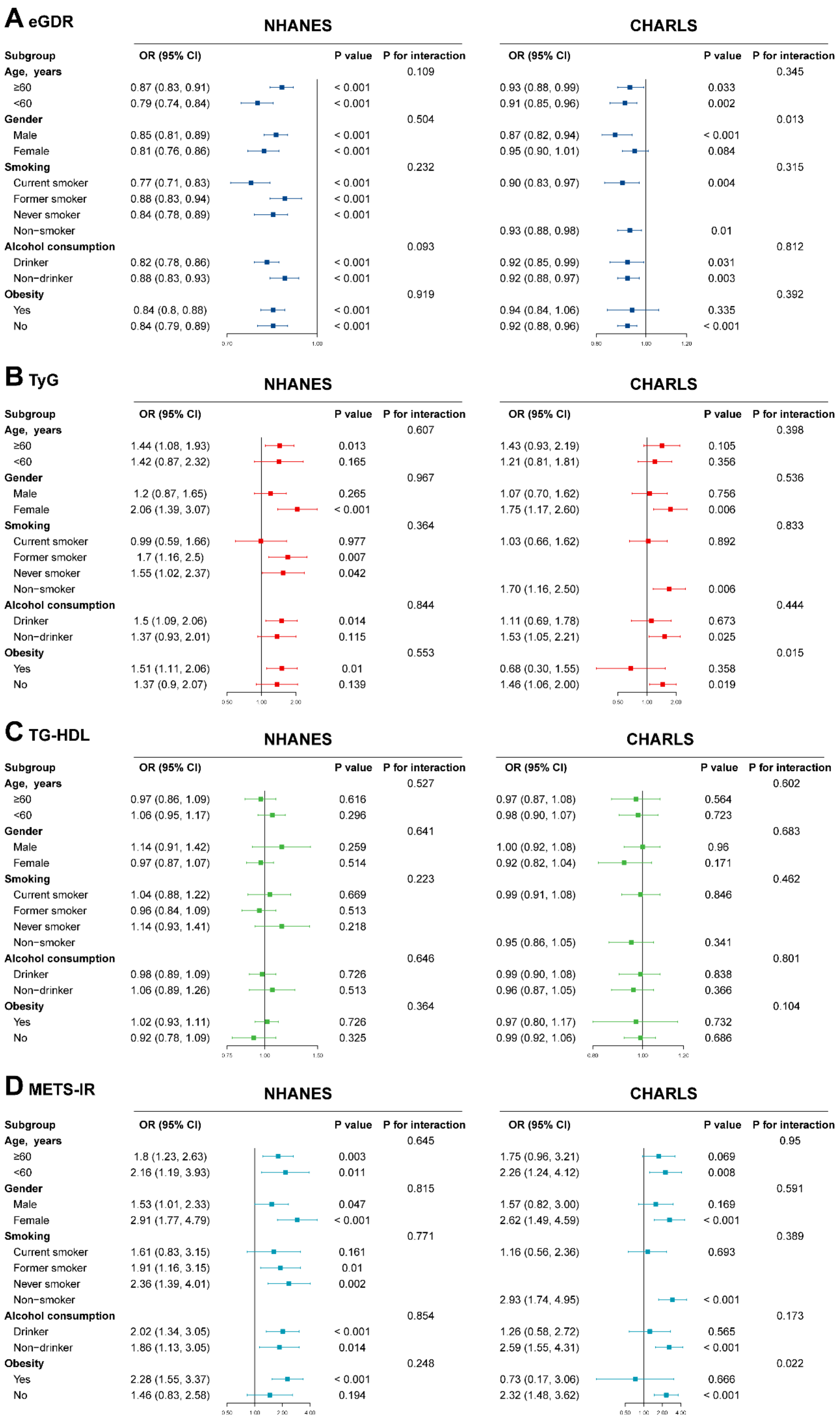

Supplementary Figure 5 Subgroup analysis of the association between (A) eGDR, (B) TyG, (C) TG-HDL, (D) METS-IR and heart disease. CHARLS, China Health and Retirement Longitudinal Study; CI, confidence interval; eGDR, estimated glucose disposal rate; TyG, triglyceride glucose; TG, triglyceride; HDL-C, high-density lipoprotein cholesterol; METS-IR, metabolic score for insulin resistance; NHANES, National Health and Nutrition Examination Survey; OR, odd ratio.

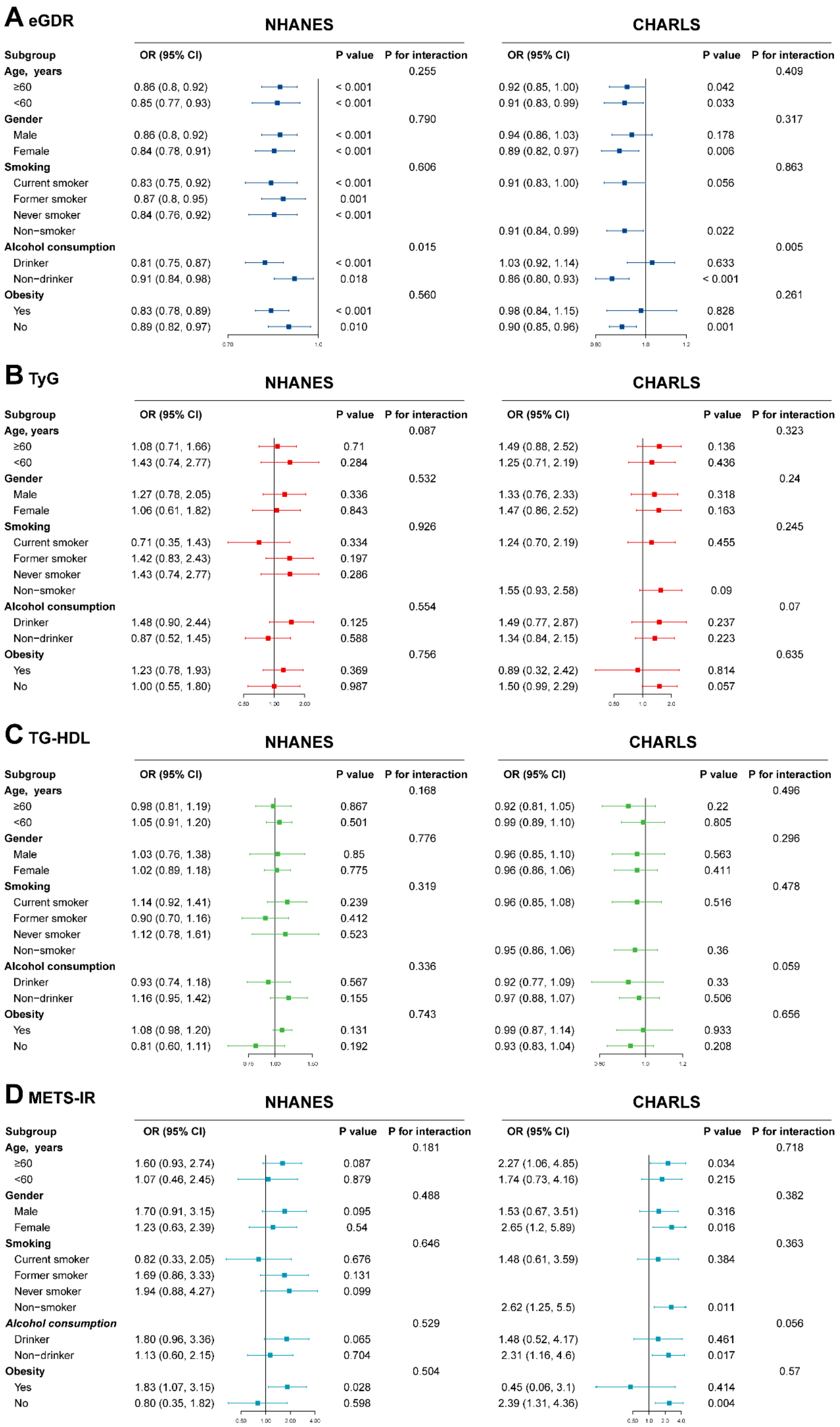

Supplementary Figure 6 Subgroup analysis of the association between (A) eGDR, (B) TyG, (C) TG-HDL, (D) METS-IR and stroke. CHARLS, China Health and Retirement Longitudinal Study; CI, confidence interval; eGDR, estimated glucose disposal rate; TyG, triglyceride glucose; TG, triglyceride; HDL-C, high-density lipoprotein cholesterol; METS-IR, metabolic score for insulin resistance; NHANES, National Health and Nutrition Examination Survey; OR, odd ratio.

A eGDR

Subgroup	NHANES OR (95% CI)	NHANES P value	NHANES P for interaction	CHARLS OR (95% CI)	CHARLS P value	CHARLS P for interaction
**Age, years**			0.063			0.454
≥60						
Q1	Ref	Ref		Ref	Ref	
Q2	0.80 (0.64, 0.98)	0.034		0.74 (0.56, 0.99)	0.044	
Q3	0.59 (0.46, 0.74)	< 0.001		0.74 (0.53, 1.02)	0.065	
Q4	0.39 (0.28, 0.53)	< 0.001		0.63 (0.45, 0.89)	0.008	
<60						
Q1	Ref	Ref		Ref	Ref	
Q2	0.71 (0.49, 1.01)	0.059		0.75 (0.56, 0.99)	0.043	
Q3	0.36 (0.24, 0.53)	< 0.001		0.57 (0.42, 0.78)	< 0.001	
Q4	0.18 (0.11, 0.30)	< 0.001		0.55 (0.40, 0.76)	< 0.001	
**Gender**			0.818			0.196
Male						
Q1	Ref	Ref		Ref	Ref	
Q2	0.80 (0.63, 1.01)	0.065		0.65 (0.48, 0.89)	0.007	
Q3	0.52 (0.40, 0.67)	< 0.001		0.53 (0.38, 0.74)	< 0.001	
Q4	0.35 (0.25, 0.50)	< 0.001		0.49 (0.35, 0.70)	< 0.001	
Female						
Q1	Ref	Ref		Ref	Ref	
Q2	0.71 (0.54, 0.95)	0.019		0.81 (0.62, 1.06)	0.123	
Q3	0.49 (0.35, 0.68)	< 0.001		0.74 (0.55, 1.00)	0.05	
Q4	0.24 (0.15, 0.37)	< 0.001		0.66 (0.49, 0.91)	0.01	
**Smoking**			0.363			0.433
Current smoker						
Q1	Ref	Ref		Ref	Ref	
Q2	0.67 (0.45, 1.01)	0.058		0.65 (0.46, 0.91)	0.014	
Q3	0.45 (0.30, 0.69)	< 0.001		0.53 (0.36, 0.77)	< 0.001	
Q4	0.17 (0.10, 0.30)	< 0.001		0.56 (0.39, 0.81)	0.002	
Former smoker						
Q1	Ref	Ref				
Q2	0.84 (0.63, 1.11)	0.211				
Q3	0.63 (0.47, 0.85)	0.003				
Q4	0.41 (0.26, 0.63)	< 0.001				
Never smoker						
Q1	Ref	Ref				
Q2	0.70 (0.51, 0.94)	0.019				
Q3	0.39 (0.27, 0.56)	< 0.001				
Q4	0.32 (0.20, 0.50)	< 0.001				
Non−smoker						
Q1				Ref	Ref	
Q2				0.80 (0.62, 1.02)	0.076	
Q3				0.71 (0.54, 0.94)	0.015	
Q4				0.59 (0.44, 0.80)	< 0.001	
**Alcohol consumption**			0.037			0.352
Drinker						
Q1	Ref	Ref		Ref	Ref	
Q2	0.64 (0.51, 0.81)	< 0.001		0.96 (0.66, 1.38)	0.82	
Q3	0.42 (0.32, 0.54)	< 0.001		0.75 (0.50, 1.13)	0.168	
Q4	0.24 (0.17, 0.34)	< 0.001		0.74 (0.49, 1.12)	0.15	
Non−drinker						
Q1	Ref	Ref		Ref	Ref	
Q2	0.98 (0.73, 1.32)	0.902		0.67 (0.52, 0.85)	0.001	
Q3	0.70 (0.50, 0.97)	0.030		0.60 (0.46, 0.78)	< 0.001	
Q4	0.44 (0.28, 0.69)	< 0.001		0.54 (0.40, 0.71)	< 0.001	
**Obesity**			0.005			0.996
Yes						
Q1	Ref	Ref		Ref	Ref	
Q2	0.83 (0.67, 1.03)	0.091		0.71 (0.44, 1.15)	0.162	
Q3	0.39 (0.30, 0.50)	< 0.001		0.71 (0.24, 2.15)	0.55	
Q4	0.22 (0.11, 0.42)	< 0.001		0.52 (0.12, 2.36)	0.399	
No						
Q1	Ref	Ref		Ref	Ref	
Q2	0.98 (0.61, 1.56)	0.927		0.75 (0.60, 0.93)	0.01	
Q3	0.85 (0.52, 1.37)	0.495		0.65 (0.51, 0.81)	< 0.001	
Q4	0.45 (0.28, 0.74)	0.002		0.59 (0.47, 0.76)	< 0.001	

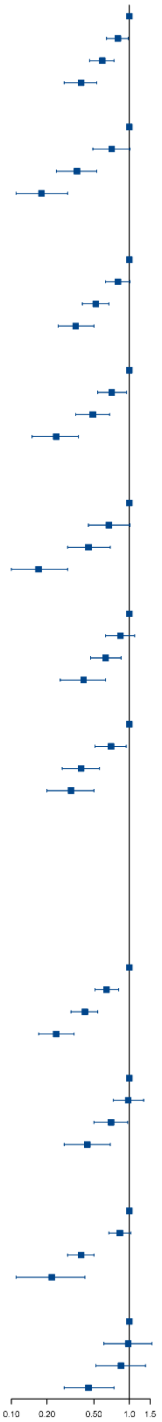

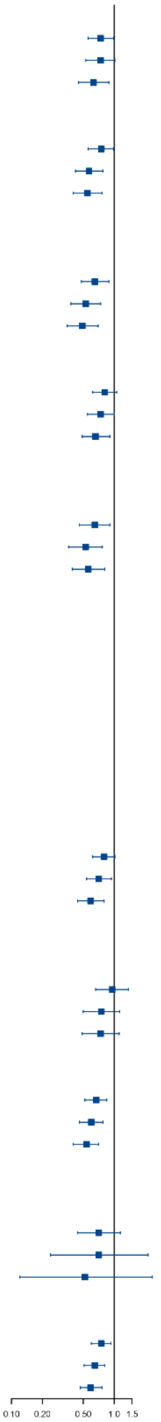

B **TyG**

Subgroup	NHANES OR (95% CI)	NHANES P value	NHANES P for interaction	CHARLS OR (95% CI)	CHARLS P value	CHARLS P for interaction
**Age, years**			0.506			0.318
≥60						
Q1	Ref	Ref		Ref	Ref	
Q2	0.92 (0.72, 1.17)	0.481		1.25 (0.89, 1.77)	0.202	
Q3	0.99 (0.76, 1.30)	0.967		1.43 (0.99, 2.06)	0.054	
Q4	1.27 (0.88, 1.83)	0.196		1.65 (1.04, 2.62)	0.034	
<60						
Q1	Ref	Ref		Ref	Ref	
Q2	0.98 (0.63, 1.53)	0.938		1.06 (0.77, 1.46)	0.714	
Q3	1.60 (1.00, 2.55)	0.049		1.23 (0.88, 1.72)	0.229	
Q4	1.96 (1.03, 3.72)	0.040		1.10 (0.71, 1.70)	0.671	
**Gender**			0.324			0.724
Male						
Q1	Ref	Ref		Ref	Ref	
Q2	0.91 (0.69, 1.19)	0.480		1.19 (0.85, 1.67)	0.319	
Q3	0.95 (0.71, 1.29)	0.762		1.53 (1.06, 2.19)	0.022	
Q4	1.28 (0.85, 1.93)	0.231		1.36 (0.86, 2.17)	0.192	
Female			0.324			
Q1	Ref	Ref		Ref	Ref	
Q2	1.01 (0.72, 1.41)	0.950		1.16 (0.84, 1.61)	0.359	
Q3	1.48 (1.02, 2.16)	0.039		1.26 (0.89, 1.77)	0.191	
Q4	1.75 (1.06, 2.91)	0.030		1.41 (0.91, 2.18)	0.12	
**Smoking**			0.422			0.511
Current smoker						
Q1	Ref	Ref		Ref	Ref	
Q2	0.87 (0.57, 1.34)	0.533		1.30 (0.89, 1.89)	0.171	
Q3	1.14 (0.71, 1.84)	0.582		1.59 (1.07, 2.37)	0.021	
Q4	1.27 (0.65, 2.50)	0.481		1.23 (0.74, 2.05)	0.418	
Former smoker						
Q1	Ref	Ref				
Q2	0.78 (0.56, 1.09)	0.149				
Q3	1.06 (0.74, 1.52)	0.749				
Q4	1.37 (0.85, 2.21)	0.202				
Never smoker						
Q1	Ref	Ref				
Q2	1.17 (0.82, 1.67)	0.382				
Q3	1.14 (0.76, 1.70)	0.531				
Q4	1.61 (0.94, 2.77)	0.086				
Non−smoker						
Q1				Ref	Ref	
Q2				1.10 (0.82, 1.49)	0.522	
Q3				1.24 (0.90, 1.70)	0.186	
Q4				1.49 (0.99, 2.24)	0.053	
**Alcohol consumption**			0.984			0.617
Drinker						
Q1	Ref	Ref		Ref	Ref	
Q2	0.92 (0.71, 1.20)	0.550		1.02 (0.69, 1.51)	0.923	
Q3	1.11 (0.83, 1.50)	0.479		1.34 (0.88, 2.03)	0.174	
Q4	1.40 (0.93, 2.11)	0.107		1.39 (0.82, 2.34)	0.223	
Non−drinker						
Q1	Ref	Ref		Ref	Ref	
Q2	0.97 (0.69, 1.38)	0.881		1.26 (0.94, 1.70)	0.122	
Q3	1.12 (0.77, 1.63)	0.553		1.36 (0.99, 1.85)	0.055	
Q4	1.42 (0.86, 2.33)	0.169		1.37 (0.92, 2.05)	0.122	
**Obesity**			0.108			0.06
Yes						
Q1	Ref	Ref		Ref	Ref	
Q2	0.81 (0.60, 1.09)	0.169		0.89 (0.40, 2.00)	0.784	
Q3	0.88 (0.64, 1.20)	0.420		0.68 (0.30, 1.54)	0.355	
Q4	1.29 (0.87, 1.92)	0.206		0.56 (0.23, 1.37)	0.203	
No						
Q1	Ref	Ref		Ref	Ref	
Q2	1.04 (0.77, 1.41)	0.788		1.17 (0.91, 1.50)	0.219	
Q3	1.40 (0.98, 2.00)	0.066		1.43 (1.10, 1.86)	0.007	
Q4	1.32 (0.76, 2.29)	0.321		1.57 (1.11, 2.22)	0.011	

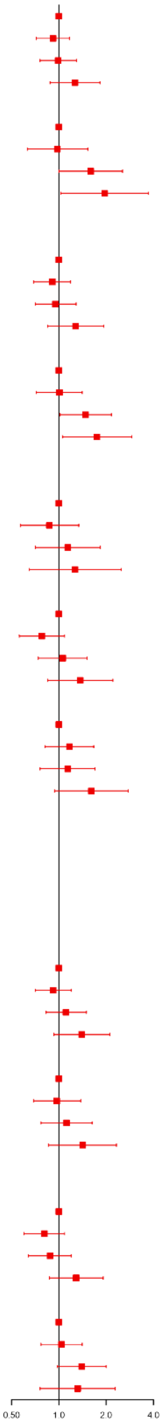

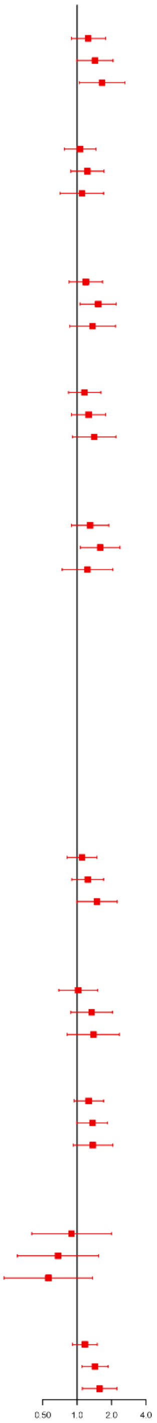

C TG-HDL

Subgroup	NHANES OR (95% CI)	NHANES P value	NHANES P for interaction	CHARLS OR (95% CI)	CHARLS P value	CHARLS P for interaction
**Age, years**			0.76			0.454
≥60						
Q1	Ref	Ref		Ref	Ref	
Q2	1.16 (0.89, 1.52)	0.275		0.80 (0.55, 1.15)	0.227	
Q3	1.10 (0.78, 1.53)	0.589		1.18 (0.79, 1.77)	0.427	
Q4	1.41 (0.86, 2.30)	0.168		1.03 (0.59, 1.80)	0.907	
<60						
Q1	Ref	Ref		Ref	Ref	
Q2	1.30 (0.82, 2.07)	0.264		1.03 (0.73, 1.46)	0.857	
Q3	1.08 (0.62, 1.89)	0.783		1.17 (0.79, 1.74)	0.443	
Q4	1.93 (0.90, 4.13)	0.090		1.09 (0.64, 1.85)	0.759	
**Gender**			0.867			0.715
Male						
Q1	Ref	Ref		Ref	Ref	
Q2	1.22 (0.89, 1.66)	0.214		1.06 (0.72, 1.56)	0.779	
Q3	1.16 (0.80, 1.67)	0.442		1.59 (1.04, 2.44)	0.032	
Q4	1.57 (0.94, 2.63)	0.082		1.40 (0.79, 2.46)	0.245	
Female						
Q1	Ref	Ref		Ref	Ref	
Q2	1.21 (0.85, 1.73)	0.284		0.84 (0.60, 1.17)	0.298	
Q3	1.10 (0.69, 1.75)	0.702		0.98 (0.67, 1.43)	0.909	
Q4	1.53 (0.76, 3.09)	0.237		0.89 (0.53, 1.50)	0.671	
**Smoking**			0.233			0.341
Current smoker						
Q1	Ref	Ref		Ref	Ref	
Q2	1.64 (1.01, 2.69)	0.047		1.35 (0.89, 2.04)	0.164	
Q3	1.50 (0.84, 2.67)	0.168		1.63 (1.02, 2.61)	0.039	
Q4	3.07 (1.38, 6.85)	0.006		1.39 (0.75, 2.56)	0.294	
Former smoker						
Q1	Ref	Ref				
Q2	1.09 (0.75, 1.57)	0.648				
Q3	1.27 (0.81, 1.99)	0.289				
Q4	1.41 (0.74, 2.68)	0.296				
Never smoker						
Q1	Ref	Ref				
Q2	1.02 (0.70, 1.49)	0.919				
Q3	0.70 (0.42, 1.14)	0.153				
Q4	0.96 (0.47, 1.98)	0.920				
Non−smoker						
Q1				Ref	Ref	
Q2				0.75 (0.55, 1.03)	0.077	
Q3				1.03 (0.72, 1.47)	0.887	
Q4				0.99 (0.60, 1.60)	0.952	
**Alcohol consumption**			0.55			0.46
Drinker						
Q1	Ref	Ref		Ref	Ref	
Q2	1.18 (0.89, 1.58)	0.251		1.01 (0.65, 1.58)	0.949	
Q3	1.08 (0.75, 1.54)	0.689		1.77 (1.10, 2.85)	0.019	
Q4	1.28 (0.76, 2.15)	0.357		1.47 (0.77, 2.80)	0.244	
Non−drinker						
Q1	Ref	Ref		Ref	Ref	
Q2	1.25 (0.85, 1.85)	0.259		0.87 (0.63, 1.18)	0.361	
Q3	1.21 (0.75, 1.94)	0.440		0.96 (0.68, 1.37)	0.83	
Q4	2.20 (1.12, 4.32)	0.022		0.88 (0.55, 1.42)	0.598	
**Obesity**			0.766			0.378
Yes						
Q1	Ref	Ref		Ref	Ref	
Q2	1.05 (0.76, 1.46)	0.748		0.85 (0.33, 2.25)	0.75	
Q3	0.87 (0.59, 1.27)	0.467		0.61 (0.23, 1.68)	0.341	
Q4	1.18 (0.70, 1.99)	0.536		0.49 (0.15, 1.57)	0.23	
No						
Q1	Ref	Ref		Ref	Ref	
Q2	1.38 (0.99, 1.93)	0.060		0.92 (0.70, 1.19)	0.515	
Q3	1.51 (0.96, 2.36)	0.073		1.26 (0.94, 1.70)	0.127	
Q4	2.11 (1.07, 4.18)	0.031		1.17 (0.77, 1.78)	0.452	

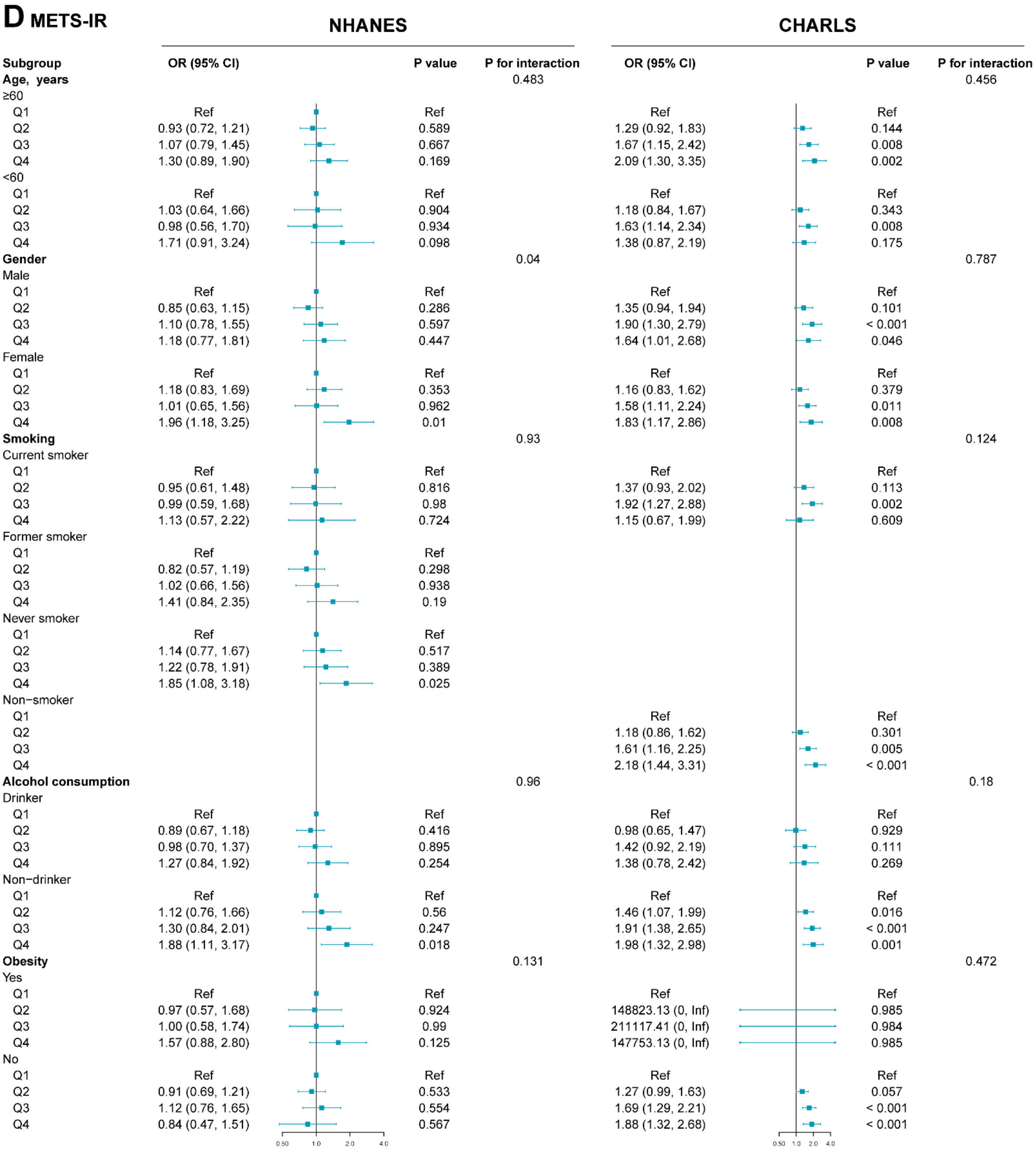

D METS-IR

Subgroup	NHANES OR (95% CI)	NHANES P value	NHANES P for interaction	CHARLS OR (95% CI)	CHARLS P value	CHARLS P for interaction
**Age, years**			0.483			0.456
≥60						
Q1	Ref	Ref		Ref	Ref	
Q2	0.93 (0.72, 1.21)	0.589		1.29 (0.92, 1.83)	0.144	
Q3	1.07 (0.79, 1.45)	0.667		1.67 (1.15, 2.42)	0.008	
Q4	1.30 (0.89, 1.90)	0.169		2.09 (1.30, 3.35)	0.002	
<60						
Q1	Ref	Ref		Ref	Ref	
Q2	1.03 (0.64, 1.66)	0.904		1.18 (0.84, 1.67)	0.343	
Q3	0.98 (0.56, 1.70)	0.934		1.63 (1.14, 2.34)	0.008	
Q4	1.71 (0.91, 3.24)	0.098		1.38 (0.87, 2.19)	0.175	
**Gender**			0.04			0.787
Male						
Q1	Ref	Ref		Ref	Ref	
Q2	0.85 (0.63, 1.15)	0.286		1.35 (0.94, 1.94)	0.101	
Q3	1.10 (0.78, 1.55)	0.597		1.90 (1.30, 2.79)	< 0.001	
Q4	1.18 (0.77, 1.81)	0.447		1.64 (1.01, 2.68)	0.046	
Female						
Q1	Ref	Ref		Ref	Ref	
Q2	1.18 (0.83, 1.69)	0.353		1.16 (0.83, 1.62)	0.379	
Q3	1.01 (0.65, 1.56)	0.962		1.58 (1.11, 2.24)	0.011	
Q4	1.96 (1.18, 3.25)	0.01		1.83 (1.17, 2.86)	0.008	
**Smoking**			0.93			0.124
Current smoker						
Q1	Ref	Ref		Ref	Ref	
Q2	0.95 (0.61, 1.48)	0.816		1.37 (0.93, 2.02)	0.113	
Q3	0.99 (0.59, 1.68)	0.98		1.92 (1.27, 2.88)	0.002	
Q4	1.13 (0.57, 2.22)	0.724		1.15 (0.67, 1.99)	0.609	
Former smoker						
Q1	Ref	Ref				
Q2	0.82 (0.57, 1.19)	0.298				
Q3	1.02 (0.66, 1.56)	0.938				
Q4	1.41 (0.84, 2.35)	0.19				
Never smoker						
Q1	Ref	Ref				
Q2	1.14 (0.77, 1.67)	0.517				
Q3	1.22 (0.78, 1.91)	0.389				
Q4	1.85 (1.08, 3.18)	0.025				
Non−smoker						
Q1				Ref	Ref	
Q2				1.18 (0.86, 1.62)	0.301	
Q3				1.61 (1.16, 2.25)	0.005	
Q4				2.18 (1.44, 3.31)	< 0.001	
**Alcohol consumption**			0.96			0.18
Drinker						
Q1	Ref	Ref		Ref	Ref	
Q2	0.89 (0.67, 1.18)	0.416		0.98 (0.65, 1.47)	0.929	
Q3	0.98 (0.70, 1.37)	0.895		1.42 (0.92, 2.19)	0.111	
Q4	1.27 (0.84, 1.92)	0.254		1.38 (0.78, 2.42)	0.269	
Non−drinker						
Q1	Ref	Ref		Ref	Ref	
Q2	1.12 (0.76, 1.66)	0.56		1.46 (1.07, 1.99)	0.016	
Q3	1.30 (0.84, 2.01)	0.247		1.91 (1.38, 2.65)	< 0.001	
Q4	1.88 (1.11, 3.17)	0.018		1.98 (1.32, 2.98)	0.001	
**Obesity**			0.131			0.472
Yes						
Q1	Ref	Ref		Ref	Ref	
Q2	0.97 (0.57, 1.68)	0.924		148823.13 (0, Inf)	0.985	
Q3	1.00 (0.58, 1.74)	0.99		211117.41 (0, Inf)	0.984	
Q4	1.57 (0.88, 2.80)	0.125		147753.13 (0, Inf)	0.985	
No						
Q1	Ref	Ref		Ref	Ref	
Q2	0.91 (0.69, 1.21)	0.533		1.27 (0.99, 1.63)	0.057	
Q3	1.12 (0.76, 1.65)	0.554		1.69 (1.29, 2.21)	< 0.001	
Q4	0.84 (0.47, 1.51)	0.567		1.88 (1.32, 2.68)	< 0.001	

Supplementary Figure 7 Subgroup and interaction analyses among the quartile 1−4 and cardiovascular diseases across various subgroups. (A) eGDR, (B) TyG, (C) TG-HDL, (D) METS-IR. eGDR, estimated glucose disposal rate. CHARLS, China Health and Retirement Longitudinal Study; CI, confidence interval; eGDR, estimated glucose disposal rate; TyG, triglyceride glucose; TG, triglyceride; HDL-C, high-density lipoprotein cholesterol; METS-IR, metabolic score for insulin resistance; NHANES, National Health and Nutrition Examination Survey; OR, odd ratio.

A eGDR

Subgroup	NHANES OR (95% CI)	NHANES P value	NHANES P for interaction	CHARLS OR (95% CI)	CHARLS P value	CHARLS P for interaction
**Age, years**			0.067			0.346
≥60						
Q1	Ref	Ref		Ref	Ref	
Q2	0.88 (0.70, 1.10)	0.249		0.91 (0.65, 1.27)	0.576	
Q3	0.64 (0.50, 0.83)	< 0.001		0.78 (0.53, 1.14)	0.202	
Q4	0.44 (0.31, 0.63)	< 0.001		0.65 (0.43, 0.97)	0.035	
<60						
Q1	Ref	Ref		Ref	Ref	
Q2	0.67 (0.45, 1.01)	0.058		0.64 (0.46, 0.90)	0.009	
Q3	0.32 (0.20, 0.50)	< 0.001		0.57 (0.40, 0.81)	0.002	
Q4	0.16 (0.09, 0.29)	< 0.001		0.61 (0.42, 0.87)	0.007	
**Gender**			0.912			0.003
Male						
Q1	Ref	Ref		Ref	Ref	
Q2	0.84 (0.65, 1.08)	0.173		0.59 (0.41, 0.86)	0.005	
Q3	0.54 (0.41, 0.71)	< 0.001		0.38 (0.25, 0.59)	< 0.001	
Q4	0.37 (0.26, 0.55)	< 0.001		0.47 (0.31, 0.71)	< 0.001	
Female						
Q1	Ref	Ref		Ref	Ref	
Q2	0.75 (0.54, 1.03)	0.075		0.89 (0.66, 1.21)	0.455	
Q3	0.50 (0.34, 0.73)	< 0.001		0.92 (0.66, 1.28)	0.617	
Q4	0.26 (0.16, 0.44)	< 0.001		0.77 (0.54, 1.10)	0.149	
**Smoking**			0.364			0.072
Current smoker						
Q1	Ref	Ref		Ref	Ref	
Q2	0.76 (0.49, 1.18)	0.216		0.64 (0.42, 0.97)	0.037	
Q3	0.42 (0.26, 0.68)	< 0.001		0.43 (0.27, 0.70)	< 0.001	
Q4	0.19 (0.10, 0.36)	< 0.001		0.64 (0.42, 1.00)	0.048	
Former smoker						
Q1	Ref	Ref				
Q2	0.81 (0.60, 1.10)	0.173				
Q3	0.70 (0.50, 0.96)	0.028				
Q4	0.41 (0.25, 0.68)	< 0.001				
Never smoker						
Q1	Ref	Ref				
Q2	0.80 (0.57, 1.11)	0.182				
Q3	0.42 (0.28, 0.64)	< 0.001				
Q4	0.37 (0.22, 0.60)	< 0.001				
Non−smoker						
Q1				Ref	Ref	
Q2				0.82 (0.62, 1.10)	0.184	
Q3				0.79 (0.58, 1.08)	0.134	
Q4				0.61 (0.44, 0.87)	0.006	
**Alcohol consumption**			0.246			0.341
Drinker						
Q1	Ref	Ref		Ref	Ref	
Q2	0.69 (0.54, 0.89)	0.004		0.76 (0.5, 1.16)	0.207	
Q3	0.46 (0.35, 0.61)	< 0.001		0.49 (0.3, 0.80)	0.004	
Q4	0.26 (0.18, 0.38)	< 0.001		0.64 (0.4, 1.01)	0.055	
Non−drinker						
Q1	Ref	Ref		Ref	Ref	
Q2	1.01 (0.74, 1.39)	0.936		0.76 (0.57, 1.01)	0.058	
Q3	0.69 (0.48, 0.99)	0.044		0.75 (0.55, 1.02)	0.063	
Q4	0.49 (0.30, 0.82)	0.006		0.64 (0.46, 0.90)	0.009	
**Obesity**			0.007			0.808
Yes						
Q1	Ref	Ref		Ref	Ref	
Q2	0.92 (0.73, 1.16)	0.487		0.70 (0.39, 1.23)	0.214	
Q3	0.40 (0.30, 0.54)	< 0.001		1.04 (0.34, 3.18)	0.949	
Q4	0.27 (0.13, 0.56)	< 0.001		0.83 (0.18, 3.79)	0.81	
No						
Q1	Ref	Ref		Ref	Ref	
Q2	0.85 (0.53, 1.38)	0.521		0.76 (0.59, 0.99)	0.042	
Q3	0.75 (0.45, 1.24)	0.263		0.66 (0.5, 0.86)	0.002	
Q4	0.40 (0.24, 0.67)	< 0.001		0.64 (0.48, 0.85)	0.002	

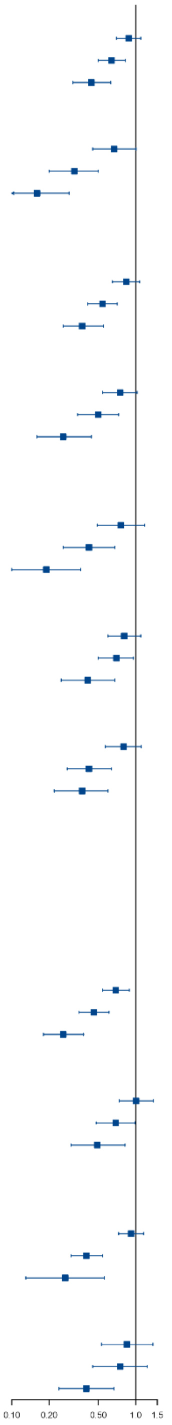

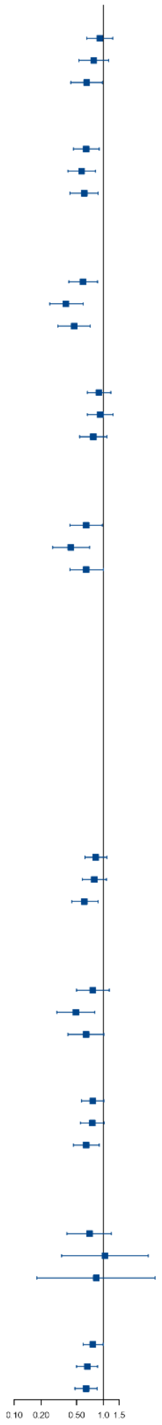

B TyG

Subgroup	NHANES OR (95% CI)	NHANES P value	NHANES P for interaction	CHARLS OR (95% CI)	CHARLS P value	CHARLS P for interaction
**Age, years**			0.937			0.71
≥60						
Q1	Ref	Ref		Ref	Ref	
Q2	0.93 (0.71, 1.21)	0.587		1.13 (0.75, 1.69)	0.557	
Q3	1.05 (0.78, 1.40)	0.756		1.45 (0.95, 2.22)	0.089	
Q4	1.30 (0.88, 1.91)	0.188		1.73 (0.99, 3.01)	0.052	
<60						
Q1	Ref	Ref		Ref	Ref	
Q2	1.00 (0.60, 1.65)	0.996		1.00 (0.69, 1.44)	0.99	
Q3	1.47 (0.86, 2.52)	0.158		1.10 (0.74, 1.62)	0.648	
Q4	2.02 (0.98, 4.15)	0.056		1.23 (0.74, 2.05)	0.422	
**Gender**			0.317			0.622
Male						
Q1	Ref	Ref		Ref	Ref	
Q2	1.02 (0.76, 1.37)	0.901		1.05 (0.69, 1.58)	0.826	
Q3	1.02 (0.74, 1.40)	0.926		1.41 (0.92, 2.16)	0.118	
Q4	1.34 (0.87, 2.06)	0.185		1.23 (0.71, 2.13)	0.468	
Female						
Q1	Ref	Ref		Ref	Ref	
Q2	0.88 (0.60, 1.30)	0.518		1.14 (0.79, 1.64)	0.487	
Q3	1.46 (0.96, 2.22)	0.080		1.27 (0.86, 1.88)	0.223	
Q4	1.81 (1.03, 3.17)	0.038		1.81 (1.09, 3.00)	0.021	
**Smoking**			0.332			0.561
Current smoker						
Q1	Ref	Ref		Ref	Ref	
Q2	0.84 (0.52, 1.36)	0.484		1.11 (0.71, 1.74)	0.655	
Q3	1.12 (0.66, 1.88)	0.674		1.43 (0.89, 2.28)	0.14	
Q4	1.39 (0.68, 2.86)	0.371		1.08 (0.59, 1.99)	0.806	
Former smoker						
Q1	Ref	Ref				
Q2	0.81 (0.56, 1.16)	0.245				
Q3	1.09 (0.74, 1.60)	0.665				
Q4	1.25 (0.75, 2.09)	0.397				
Never smoker						
Q1	Ref	Ref				
Q2	1.25 (0.84, 1.86)	0.270				
Q3	1.19 (0.76, 1.86)	0.448				
Q4	1.87 (1.04, 3.37)	0.036				
Non−smoker						
Q1				Ref	Ref	
Q2				1.08 (0.77, 1.52)	0.658	
Q3				1.25 (0.87, 1.81)	0.227	
Q4				1.86 (1.15, 3.00)	0.011	
**Alcohol consumption**			0.924			0.235
Drinker						
Q1	Ref	Ref		Ref	Ref	
Q2	0.92 (0.69, 1.24)	0.592		0.80 (0.50, 1.27)	0.344	
Q3	1.11 (0.80, 1.53)	0.532		1.22 (0.76, 1.96)	0.41	
Q4	1.42 (0.91, 2.20)	0.120		1.05 (0.57, 1.95)	0.866	
Non−drinker						
Q1	Ref	Ref		Ref	Ref	
Q2	1.03 (0.70, 1.53)	0.873		1.28 (0.91, 1.80)	0.162	
Q3	1.18 (0.78, 1.80)	0.426		1.36 (0.94, 1.96)	0.102	
Q4	1.47 (0.86, 2.53)	0.161		1.82 (1.13, 2.93)	0.013	
**Obesity**			0.162			0.028
Yes						
Q1	Ref	Ref		Ref	Ref	
Q2	0.88 (0.63, 1.23)	0.467		0.69 (0.29, 1.67)	0.415	
Q3	0.90 (0.64, 1.27)	0.550		0.48 (0.19, 1.19)	0.112	
Q4	1.29 (0.84, 1.99)	0.238		0.57 (0.21, 1.59)	0.284	
No						
Q1	Ref	Ref		Ref	Ref	
Q2	1.00 (0.72, 1.40)	0.982		1.08 (0.81, 1.44)	0.58	
Q3	1.49 (1.01, 2.20)	0.043		1.38 (1.02, 1.88)	0.037	
Q4	1.53 (0.85, 2.75)	0.160		1.66 (1.11, 2.49)	0.015	

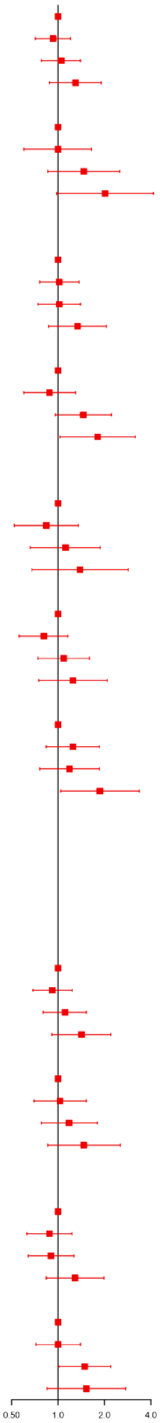

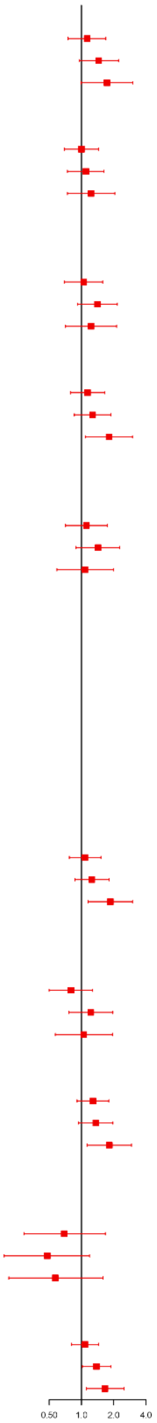

C TG-HDL

Subgroup	NHANES OR (95% CI)	NHANES P value	NHANES P for interaction	CHARLS OR (95% CI)	CHARLS P value	CHARLS P for interaction
**Age, years**			0.505			0.271
≥60						
Q1	Ref	Ref		Ref	Ref	
Q2	1.21 (0.90, 1.63)	0.203		0.60 (0.39, 0.94)	0.024	
Q3	1.20 (0.83, 1.72)	0.328		1.07 (0.67, 1.71)	0.785	
Q4	1.44 (0.86, 2.44)	0.169		1.01 (0.52, 1.94)	0.979	
<60						
Q1	Ref	Ref		Ref	Ref	
Q2	1.13 (0.67, 1.92)	0.651		0.96 (0.65, 1.43)	0.858	
Q3	0.98 (0.52, 1.84)	0.953		1.16 (0.73, 1.82)	0.531	
Q4	1.30 (0.55, 3.07)	0.554		1.05 (0.57, 1.95)	0.873	
**Gender**			0.835			0.694
Male						
Q1	Ref	Ref		Ref	Ref	
Q2	1.29 (0.92, 1.80)	0.141		0.86 (0.54, 1.38)	0.536	
Q3	1.20 (0.81, 1.78)	0.361		1.48 (0.90, 2.42)	0.125	
Q4	1.49 (0.87, 2.58)	0.149		1.14 (0.59, 2.23)	0.694	
Female						
Q1	Ref	Ref		Ref	Ref	
Q2	1.14 (0.76, 1.72)	0.525		0.76 (0.52, 1.12)	0.164	
Q3	1.20 (0.71, 2.03)	0.508		0.99 (0.64, 1.53)	0.967	
Q4	1.40 (0.63, 3.11)	0.404		1.03 (0.57, 1.88)	0.919	
**Smoking**						0.954
Current smoker			0.197			
Q1	Ref	Ref		Ref	Ref	
Q2	1.49 (0.86, 2.58)	0.153		0.92 (0.56, 1.52)	0.757	
Q3	1.50 (0.80, 2.81)	0.210		1.26 (0.73, 2.17)	0.409	
Q4	2.53 (1.05, 6.05)	0.038		1.07 (0.52, 2.20)	0.847	
Former smoker						
Q1	Ref	Ref				
Q2	1.18 (0.78, 1.77)	0.436				
Q3	1.32 (0.81, 2.15)	0.264				
Q4	1.19 (0.59, 2.40)	0.622				
Never smoker						
Q1	Ref	Ref				
Q2	1.02 (0.67, 1.56)	0.919				
Q3	0.79 (0.46, 1.36)	0.392				
Q4	1.14 (0.52, 2.49)	0.740				
Non−smoker						
Q1				Ref	Ref	
Q2				0.74 (0.52, 1.07)	0.107	
Q3				1.11 (0.74, 1.67)	0.614	
Q4				1.12 (0.63, 1.99)	0.71	
**Alcohol consumption**						0.649
Drinker			0.826			
Q1	Ref	Ref		Ref	Ref	
Q2	1.17 (0.85, 1.61)	0.330		0.84 (0.51, 1.39)	0.498	
Q3	1.16 (0.78, 1.71)	0.468		1.41 (0.82, 2.43)	0.216	
Q4	1.29 (0.73, 2.26)	0.375		1.00 (0.47, 2.12)	1	
Non−drinker						
Q1	Ref	Ref		Ref	Ref	
Q2	1.32 (0.85, 2.04)	0.217		0.76 (0.53, 1.09)	0.133	
Q3	1.27 (0.75, 2.15)	0.367		1.01 (0.67, 1.52)	0.951	
Q4	1.85 (0.88, 3.85)	0.103		1.04 (0.59, 1.82)	0.901	
**Obesity**			0.622			0.095
Yes						
Q1	Ref	Ref		Ref	Ref	
Q2	1.00 (0.69, 1.44)	0.993		0.88 (0.31, 2.51)	0.818	
Q3	0.90 (0.60, 1.37)	0.631		0.61 (0.20, 1.84)	0.376	
Q4	1.08 (0.61, 1.91)	0.783		0.53 (0.14, 2.04)	0.36	
No						
Q1	Ref	Ref		Ref	Ref	
Q2	1.50 (1.03, 2.18)	0.034		0.75 (0.55, 1.03)	0.073	
Q3	1.67 (1.02, 2.74)	0.041		1.18 (0.83, 1.66)	0.356	
Q4	2.10 (0.99, 4.45)	0.053		1.11 (0.69, 1.81)	0.659	

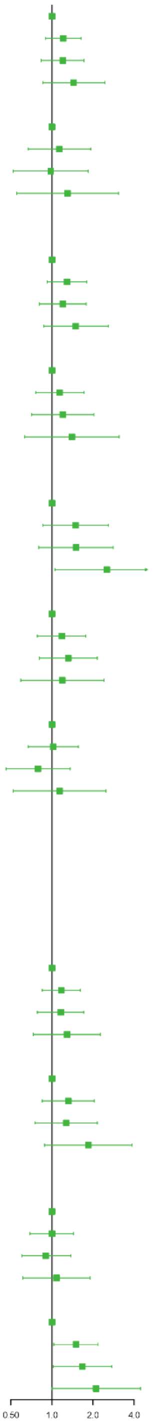

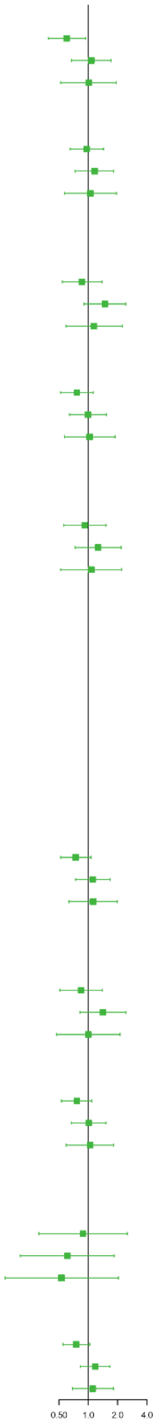

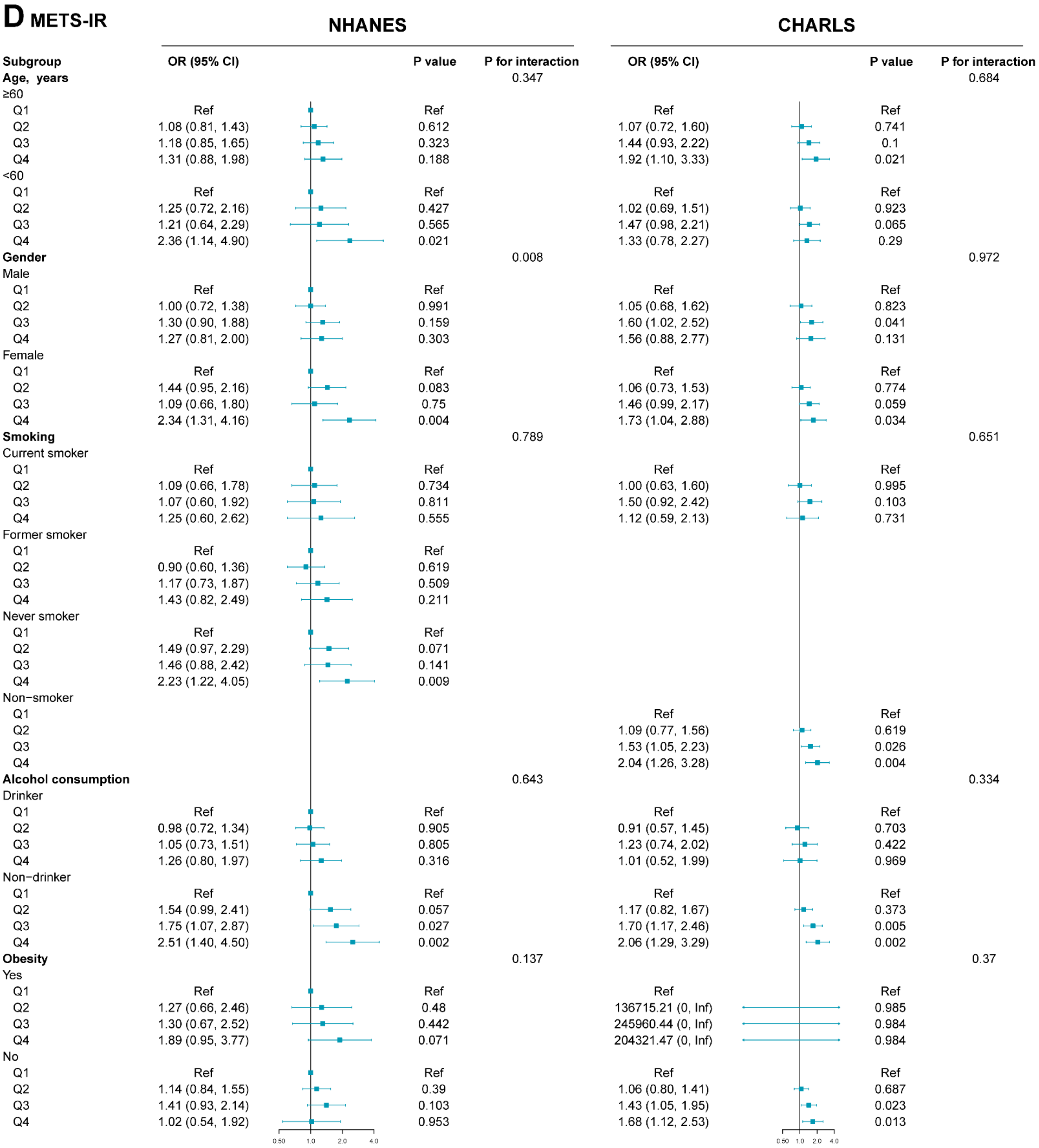

Supplementary Figure 8 Subgroup and interaction analyses among the quartile 1−4 and heart disease across various subgroups. (A) eGDR, (B) TyG, (C) TG-HDL, (D) METS-IR. eGDR, estimated glucose disposal rate. CHARLS, China Health and Retirement Longitudinal Study; CI, confidence interval; eGDR, estimated glucose disposal rate; TyG, triglyceride glucose; TG, triglyceride; HDL-C, high-density lipoprotein cholesterol; METS-IR, metabolic score for insulin resistance; NHANES, National Health and Nutrition Examination Survey; OR, odd ratio.

A eGDR

Subgroup	NHANES OR (95% CI)	NHANES P value	NHANES P for interaction	CHARLS OR (95% CI)	CHARLS P value	CHARLS P for interaction
**Age, years**						0.056
≥60			0.29			
Q1	Ref	Ref				
Q2	0.81 (0.59, 1.13)	0.216		0.54 (0.34, 0.87)	0.011	
Q3	0.64 (0.44, 0.93)	0.019		0.76 (0.46, 1.23)	0.262	
Q4	0.34 (0.20, 0.59)	< 0.001		0.66 (0.40, 1.10)	0.114	
<60						
Q1	Ref	Ref				
Q2	0.70 (0.40, 1.21)	0.201		1.05 (0.67, 1.65)	0.841	
Q3	0.47 (0.26, 0.84)	0.01		0.74 (0.45, 1.23)	0.249	
Q4	0.18 (0.08, 0.41)	< 0.001		0.52 (0.29, 0.95)	0.032	
**Gender**			0.861			0.372
Male						
Q1	Ref	Ref				
Q2	0.71 (0.48, 1.05)	0.089		0.92 (0.57, 1.49)	0.731	
Q3	0.57 (0.38, 0.86)	0.008		0.99 (0.60, 1.65)	0.983	
Q4	0.28 (0.15, 0.51)	< 0.001		0.63 (0.35, 1.12)	0.116	
Female						
Q1	Ref	Ref				
Q2	0.87 (0.58, 1.31)	0.511		0.67 (0.43, 1.03)	0.067	
Q3	0.62 (0.39, 1.01)	0.053		0.55 (0.33, 0.93)	0.024	
Q4	0.25 (0.13, 0.49)	< 0.001		0.56 (0.33, 0.96)	0.035	
**Smoking**			0.335			0.782
Current smoker						
Q1	Ref	Ref				
Q2	0.58 (0.32, 1.04)	0.067		0.79 (0.47, 1.32)	0.363	
Q3	0.65 (0.37, 1.15)	0.141		0.82 (0.48, 1.41)	0.472	
Q4	0.16 (0.07, 0.40)	< 0.001		0.50 (0.27, 0.93)	0.027	
Former smoker						
Q1	Ref	Ref				
Q2	1.03 (0.68, 1.58)	0.877				
Q3	0.61 (0.37, 0.99)	0.046				
Q4	0.37 (0.17, 0.77)	0.008				
Never smoker						
Q1	Ref	Ref				
Q2	0.61 (0.37, 1.00)	0.052				
Q3	0.44 (0.24, 0.81)	0.008				
Q4	0.28 (0.13, 0.59)	< 0.001				
Non−smoker						
Q1						
Q2				0.74 (0.49, 1.12)	0.152	
Q3				0.67 (0.42, 1.07)	0.095	
Q4				0.65 (0.39, 1.08)	0.096	
**Alcohol consumption**			0.191			0.002
Drinker						
Q1	Ref	Ref				
Q2	0.64 (0.43, 0.94)	0.024		1.75 (0.91, 3.35)	0.091	
Q3	0.45 (0.29, 0.70)	< 0.001		2.00 (1.00, 3.97)	0.049	
Q4	0.19 (0.10, 0.36)	< 0.001		1.12 (0.52, 2.42)	0.764	
Non−drinker						
Q1	Ref	Ref				
Q2	0.96 (0.64, 1.43)	0.835		0.57 (0.38, 0.83)	0.004	
Q3	0.79 (0.50, 1.23)	0.295		0.48 (0.31, 0.75)	0.001	
Q4	0.41 (0.21, 0.80)	0.009		0.47 (0.30, 0.75)	0.002	
**Obesity**			0.022			0.289
Yes						
Q1	Ref	Ref				
Q2	0.83 (0.60, 1.15)	0.264		0.98 (0.48, 2.01)	0.951	
Q3	0.40 (0.26, 0.61)	< 0.001		1.00 (0, Inf)	0.983	
Q4	0.11 (0.03, 0.45)	0.002		0.85 (0.10, 6.94)	0.88	
No						
Q1	Ref	Ref				
Q2	1.38 (0.63, 3.06)	0.421		0.71 (0.50, 1.02)	0.063	
Q3	1.44 (0.65, 3.23)	0.372		0.73 (0.51, 1.05)	0.093	
Q4	0.65 (0.27, 1.52)	0.315		0.56 (0.38, 0.84)	0.005	

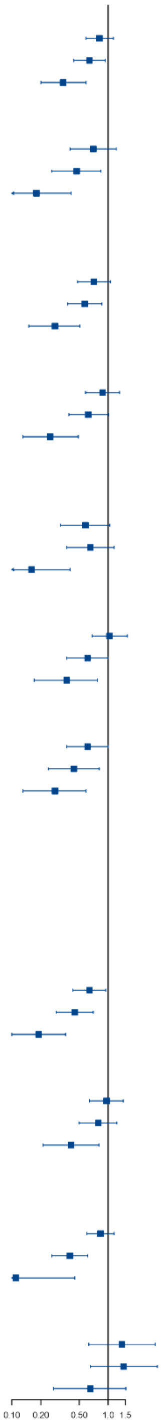

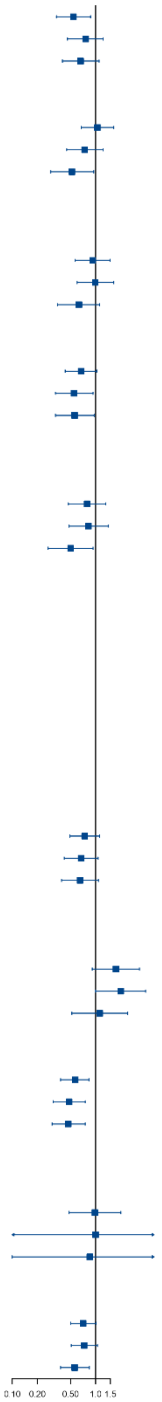

B TyG

Subgroup	NHANES OR (95% CI)	NHANES P value	NHANES P for interaction	CHARLS OR (95% CI)	CHARLS P value	CHARLS P for interaction
**Age, years**			0.221			0.305
≥60						
Q1	Ref	Ref				
Q2	0.86 (0.60, 1.23)	0.402		1.48 (0.84, 2.61)	0.174	
Q3	0.80 (0.53, 1.22)	0.304		1.41 (0.78, 2.56)	0.26	
Q4	1.08 (0.62, 1.91)	0.781		1.55 (0.76, 3.16)	0.229	
<60						
Q1	Ref	Ref				
Q2	0.86 (0.44, 1.65)	0.644		1.29 (0.72, 2.31)	0.396	
Q3	1.26 (0.62, 2.54)	0.528		1.57 (0.87, 2.83)	0.132	
Q4	1.28 (0.48, 3.43)	0.62		0.95 (0.45, 2.01)	0.899	
**Gender**			0.279			0.843
Male						
Q1	Ref	Ref				
Q2	0.72 (0.47, 1.11)	0.142		1.56 (0.89, 2.72)	0.12	
Q3	0.88 (0.54, 1.41)	0.584		1.97 (1.10, 3.53)	0.023	
Q4	1.30 (0.69, 2.48)	0.416		1.71 (0.81, 3.63)	0.162	
Female						
Q1	Ref	Ref				
Q2	1.02 (0.65, 1.61)	0.922		1.28 (0.70, 2.32)	0.421	
Q3	0.89 (0.51, 1.56)	0.69		1.18 (0.64, 2.17)	0.592	
Q4	0.87 (0.40, 1.87)	0.713		0.95 (0.45, 1.99)	0.891	
**Smoking**			0.218			0.883
Current smoker						
Q1	Ref	Ref				
Q2	0.76 (0.41, 1.42)	0.393		1.56 (0.86, 2.83)	0.143	
Q3	0.91 (0.46, 1.81)	0.799		1.92 (1.03, 3.57)	0.04	
Q4	0.85 (0.32, 2.27)	0.752		1.54 (0.70, 3.36)	0.284	
Former smoker						
Q1	Ref	Ref				
Q2	0.85 (0.52, 1.38)	0.51				
Q3	0.68 (0.38, 1.21)	0.186				
Q4	1.44 (0.71, 2.90)	0.314				
Never smoker						
Q1	Ref	Ref				
Q2	0.97 (0.56, 1.69)	0.907				
Q3	1.15 (0.60, 2.23)	0.675				
Q4	1.01 (0.38, 2.65)	0.99				
Non−smoker						
Q1						
Q2				1.33 (0.76, 2.33)	0.31	
Q3				1.29 (0.73, 2.27)	0.386	
Q4				1.11 (0.56, 2.20)	0.758	
**Alcohol consumption**						0.785
Drinker			0.647			
Q1	Ref	Ref				
Q2	1.00 (0.66, 1.53)	0.988		1.64 (0.85, 3.15)	0.141	
Q3	0.94 (0.57, 1.57)	0.826		1.64 (0.80, 3.39)	0.179	
Q4	1.23 (0.61, 2.48)	0.56		2.16 (0.89, 5.21)	0.088	
Non−drinker						
Q1	Ref	Ref				
Q2	0.66 (0.41, 1.07)	0.09		1.34 (0.80, 2.25)	0.269	
Q3	0.80 (0.48, 1.34)	0.4		1.45 (0.86, 2.45)	0.165	
Q4	0.91 (0.46, 1.80)	0.782		0.99 (0.52, 1.89)	0.968	
**Obesity**			0.273			0.59
Yes						
Q1	Ref	Ref				
Q2	0.65 (0.42, 1.00)	0.05		2.17 (0.43, 10.91)	0.346	
Q3	0.66 (0.41, 1.07)	0.09		1.70 (0.33, 8.65)	0.522	
Q4	0.93 (0.51, 1.69)	0.812		0.93 (0.16, 5.21)	0.93	
No						
Q1	Ref	Ref				
Q2	1.09 (0.69, 1.72)	0.718		1.36 (0.89, 2.08)	0.152	
Q3	1.15 (0.65, 2.01)	0.634		1.55 (1.00, 2.41)	0.051	
Q4	1.14 (0.48, 2.71)	0.759		1.42 (0.81, 2.48)	0.222	

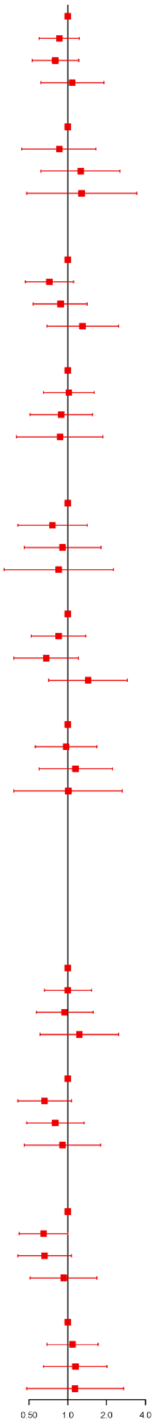

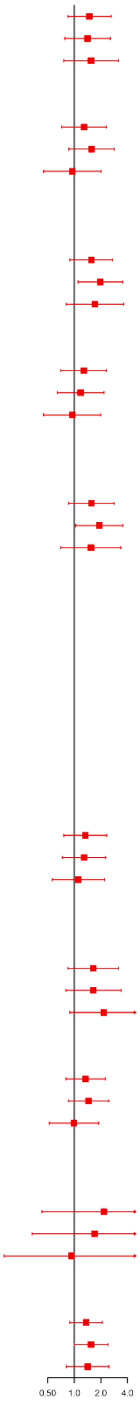

C TG-HDL

Subgroup	NHANES OR (95% CI)	NHANES P value	NHANES P for interaction	CHARLS OR (95% CI)	CHARLS P value	CHARLS P for interaction
Age, years			0.204			0.98
>=60						
Q1	Ref	Ref		Ref	Ref	
Q2	1.02 (0.68, 1.53)	0.930		1.55 (0.86, 2.79)	0.149	
Q3	1.10 (0.65, 1.84)	0.727		1.44 (0.74, 2.82)	0.285	
Q4	1.63 (0.76, 3.48)	0.209		1.31 (0.55, 3.10)	0.546	
<60						
Q1	Ref	Ref		Ref	Ref	
Q2	1.49 (0.73, 3.02)	0.273		1.53 (0.80, 2.90)	0.199	
Q3	1.05 (0.43, 2.56)	0.914		1.36 (0.66, 2.79)	0.407	
Q4	3.04 (0.94, 9.81)	0.063		1.29 (0.52, 3.18)	0.58	
Gender			0.136			0.834
Male						
Q1	Ref	Ref		Ref	Ref	
Q2	0.96 (0.58, 1.59)	0.882		2.05 (1.08, 3.89)	0.028	
Q3	1.28 (0.71, 2.30)	0.413		2.10 (1.01, 4.38)	0.046	
Q4	1.93 (0.84, 4.42)	0.120		2.46 (0.96, 6.27)	0.06	
Female						
Q1	Ref	Ref		Ref	Ref	
Q2	1.23 (0.75, 2.02)	0.417		1.15 (0.63, 2.09)	0.642	
Q3	0.78 (0.39, 1.56)	0.482		1.00 (0.51, 1.97)	0.99	
Q4	1.65 (0.60, 4.50)	0.329		0.76 (0.32, 1.82)	0.542	
Smoking			0.211			0.192
Current smoker						
Q1	Ref	Ref		Ref	Ref	
Q2	1.40 (0.70, 2.80)	0.347		3.04 (1.50, 6.16)	0.002	
Q3	0.96 (0.41, 2.23)	0.923		2.70 (1.21, 6.03)	0.016	
Q4	2.42 (0.77, 7.61)	0.132		2.64 (0.97, 7.21)	0.058	
Former smoker						
Q1	Ref	Ref				
Q2	0.94 (0.54, 1.63)	0.817				
Q3	1.34 (0.68, 2.64)	0.393				
Q4	2.32 (0.90, 6.02)	0.082				
Never smoker						
Q1	Ref	Ref				
Q2	1.10 (0.59, 2.02)	0.768				
Q3	0.89 (0.38, 2.07)	0.781				
Q4	1.02 (0.28, 3.74)	0.971				
Non−smoker						
Q1				Ref	Ref	
Q2				0.95 (0.54, 1.67)	0.855	
Q3				0.95 (0.51, 1.78)	0.875	
Q4				0.87 (0.39, 1.93)	0.734	
Alcohol consumption			0.236			0.661
Drinker						
Q1	Ref	Ref		Ref	Ref	
Q2	1.17 (0.73, 1.87)	0.506		2.11 (1.00, 4.45)	0.051	
Q3	1.10 (0.60, 2.01)	0.769		2.76 (1.17, 6.48)	0.02	
Q4	1.32 (0.54, 3.23)	0.538		3.08 (1.00, 9.55)	0.051	
Non−drinker						
Q1	Ref	Ref		Ref	Ref	
Q2	0.96 (0.56, 1.62)	0.865		1.33 (0.77, 2.27)	0.307	
Q3	0.90 (0.47, 1.75)	0.763		1.04 (0.56, 1.90)	0.911	
Q4	2.36 (0.94, 5.93)	0.068		0.88 (0.41, 1.90)	0.747	
Obesity			0.504			0.966
Yes						
Q1	Ref	Ref		Ref	Ref	
Q2	1.01 (0.63, 1.63)	0.974		2.10 (0.23, 19.34)	0.511	
Q3	0.80 (0.45, 1.45)	0.467		1.56 (0.17, 14.79)	0.696	
Q4	1.56 (0.70, 3.51)	0.277		1.30 (0.12, 14.60)	0.832	
No						
Q1	Ref	Ref		Ref	Ref	
Q2	1.15 (0.68, 1.93)	0.606		1.54 (0.99, 2.42)	0.058	
Q3	1.48 (0.74, 2.95)	0.268		1.47 (0.88, 2.47)	0.143	
Q4	2.15 (0.75, 6.13)	0.152		1.38 (0.70, 2.71)	0.353	

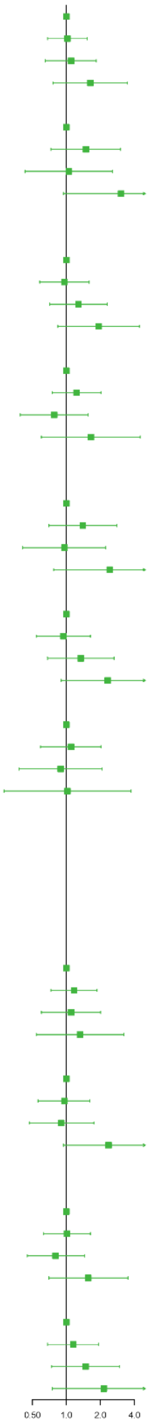

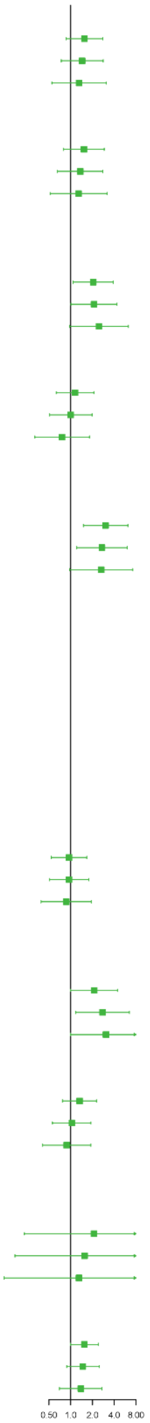

D METS-IR

Subgroup	NHANES OR (95% CI)	NHANES P value	NHANES P for interaction	CHARLS OR (95% CI)	CHARLS P value	CHARLS P for interaction
**Age, years**			0.527			0.911
≥60						
Q1	Ref	Ref		Ref	Ref	
Q2	0.96 (0.65, 1.43)	0.856		1.89 (1.06, 3.38)	0.032	
Q3	0.97 (0.60, 1.55)	0.899		2.13 (1.14, 3.97)	0.017	
Q4	1.29 (0.73, 2.29)	0.382		2.27 (1.06, 4.86)	0.034	
<60						
Q1	Ref	Ref		Ref	Ref	
Q2	0.67 (0.33, 1.36)	0.265		1.73 (0.91, 3.30)	0.093	
Q3	0.51 (0.22, 1.18)	0.118		1.96 (1.01, 3.82)	0.048	
Q4	0.70 (0.27, 1.83)	0.464		1.47 (0.65, 3.31)	0.349	
**Gender**			0.66			0.538
Male						
Q1	Ref	Ref		Ref	Ref	
Q2	0.85 (0.53, 1.37)	0.508		2.31 (1.27, 4.22)	0.006	
Q3	0.97 (0.56, 1.67)	0.903		2.54 (1.33, 4.84)	0.005	
Q4	1.36 (0.70, 2.66)	0.36		2.08 (0.93, 4.63)	0.073	
Female						
Q1	Ref	Ref		Ref	Ref	
Q2	0.88 (0.53, 1.47)	0.636		1.37 (0.74, 2.54)	0.311	
Q3	0.65 (0.35, 1.22)	0.179		1.73 (0.91, 3.26)	0.092	
Q4	0.85 (0.41, 1.77)	0.66		1.69 (0.78, 3.66)	0.182	
**Smoking**			0.697			0.235
Current smoker						
Q1	Ref	Ref		Ref	Ref	
Q2	0.65 (0.33, 1.26)	0.202		2.56 (1.35, 4.84)	0.004	
Q3	0.77 (0.36, 1.65)	0.502		2.77 (1.40, 5.47)	0.003	
Q4	0.66 (0.25, 1.76)	0.409		1.68 (0.70, 4.02)	0.242	
Former smoker						
Q1	Ref	Ref				
Q2	1.06 (0.62, 1.84)	0.822				
Q3	0.91 (0.47, 1.77)	0.79				
Q4	1.48 (0.69, 3.19)	0.312				
Never smoker						
Q1	Ref	Ref				
Q2	0.80 (0.44, 1.46)	0.473				
Q3	0.75 (0.36, 1.57)	0.448				
Q4	1.21 (0.51, 2.87)	0.659				
Non−smoker						
Q1				Ref	Ref	
Q2				1.35 (0.75, 2.41)	0.316	
Q3				1.71 (0.94, 3.11)	0.08	
Q4				1.94 (0.95, 3.96)	0.07	
**Alcohol consumption**			0.907			0.188
Drinker						
Q1	Ref	Ref		Ref	Ref	
Q2	0.93 (0.58, 1.47)	0.753		1.26 (0.64, 2.50)	0.5	
Q3	0.95 (0.54, 1.68)	0.87		1.57 (0.76, 3.27)	0.227	
Q4	1.28 (0.65, 2.52)	0.479		2.18 (0.88, 5.43)	0.093	
Non−drinker						
Q1				Ref	Ref	
Q2	0.79 (0.47, 1.32)	0.37		2.40 (1.34, 4.28)	0.003	
Q3	0.67 (0.36, 1.21)	0.184		2.62 (1.43, 4.79)	0.002	
Q4	0.90 (0.44, 1.85)	0.78		1.97 (0.96, 4.03)	0.065	
**Obesity**			0.516			0.947
Yes						
Q1	Ref	Ref		Ref	Ref	
Q2	1.07 (0.51, 2.27)	0.858		89877.01 (0, Inf)	0.991	
Q3	0.85 (0.39, 1.85)	0.684		109307.91 (0, Inf)	0.991	
Q4	1.32 (0.58, 3.00)	0.503		63866.52 (0, Inf)	0.991	
No						
Q1	Ref	Ref		Ref	Ref	
Q2	0.67 (0.43, 1.04)	0.072		1.86 (1.20, 2.87)	0.005	
Q3	0.75 (0.41, 1.39)	0.363		2.16 (1.35, 3.44)	0.001	
Q4	0.75 (0.30, 1.85)	0.528		2.13 (1.18, 3.84)	0.012	

Supplementary Figure 9 Subgroup and interaction analyses among the quartile 1−4 and stroke across various subgroups. (A) eGDR, (B) TyG, (C) TG-HDL, (D) METS-IR. eGDR, estimated glucose disposal rate. CHARLS, China Health and Retirement Longitudinal Study; CI, confidence interval; eGDR, estimated glucose disposal rate; TyG, triglyceride glucose; TG, triglyceride; HDL-C, high-density lipoprotein cholesterol; METS-IR, metabolic score for insulin resistance; NHANES, National Health and Nutrition Examination Survey; OR, odd ratio.

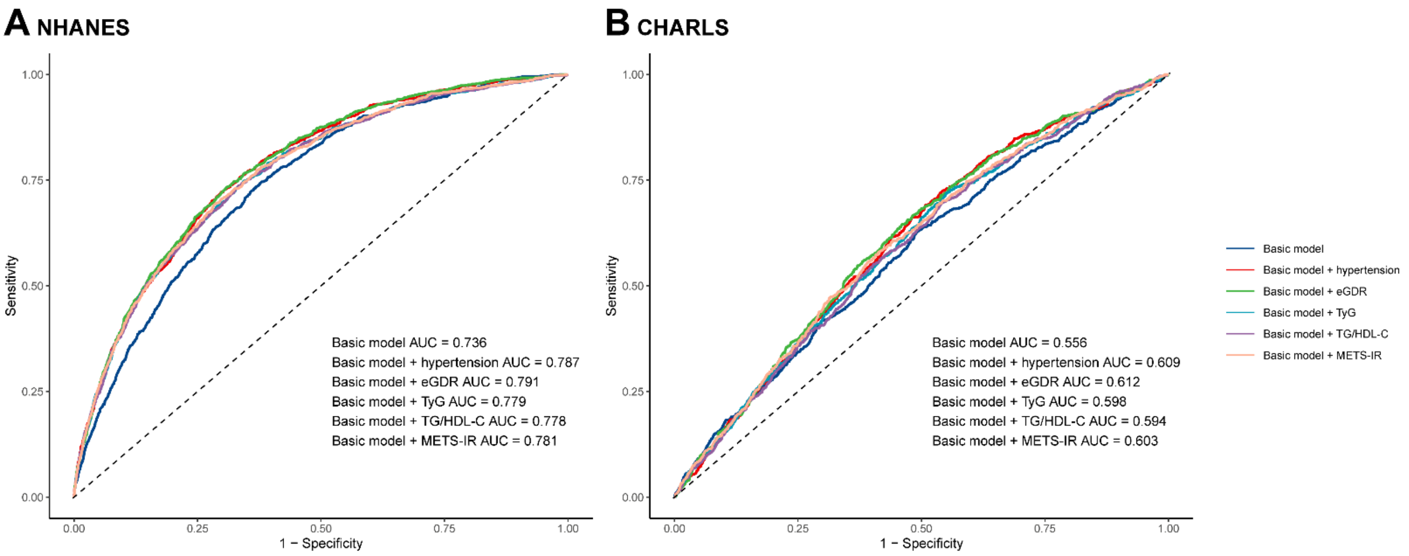

Supplementary Figure 10 The receiver operating characteristic curves of the eGDR, TyG, TG/HDL-C and METS-IR to predict heart disease. The basic model adjusted age, sex, marital status, education, smoking, alcohol consumption status, total cholesterol, high-density lipoprotein cholesterol, triglyceride, low-density lipoprotein cholesterol, blood urea nitrogen, uric acid, hemoglobin, and obesity. eGDR, estimated glucose disposal rate; TyG, triglyceride glucose; HDL-C, high-density lipoprotein cholesterol; METS-IR, metabolic score for insulin resistance.

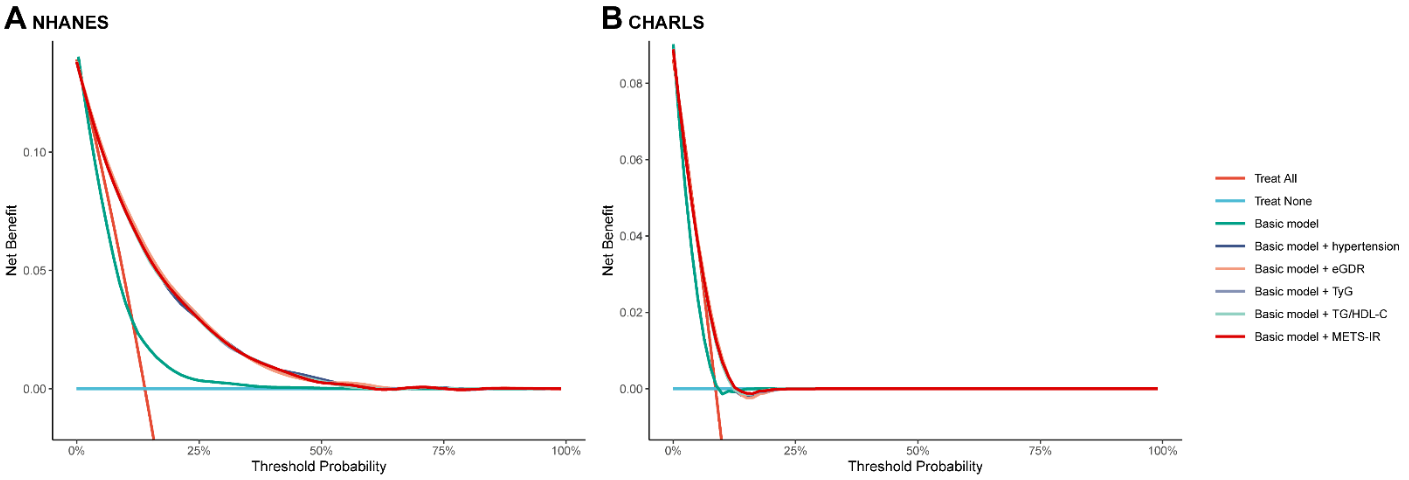

Supplementary Figure 11 The decision curve analysis of eGDR, TyG, TG/HDL-C and METS-IR to compare the clinical utility for heart disease, the y-axis represents net benefits, calculated by subtracting the relative harm (false positives) from the benefits (true positives). The x-axis calculates the threshold probability. The basic model adjusted age, sex, marital status, education, smoking, alcohol consumption status, total cholesterol, high-density lipoprotein cholesterol, triglyceride, low-density lipoprotein cholesterol, blood urea nitrogen, uric acid, hemoglobin, and obesity. eGDR, estimated glucose disposal rate; TyG, triglyceride glucose; HDL-C, high-density lipoprotein cholesterol; METS-IR, metabolic score for insulin resistance.

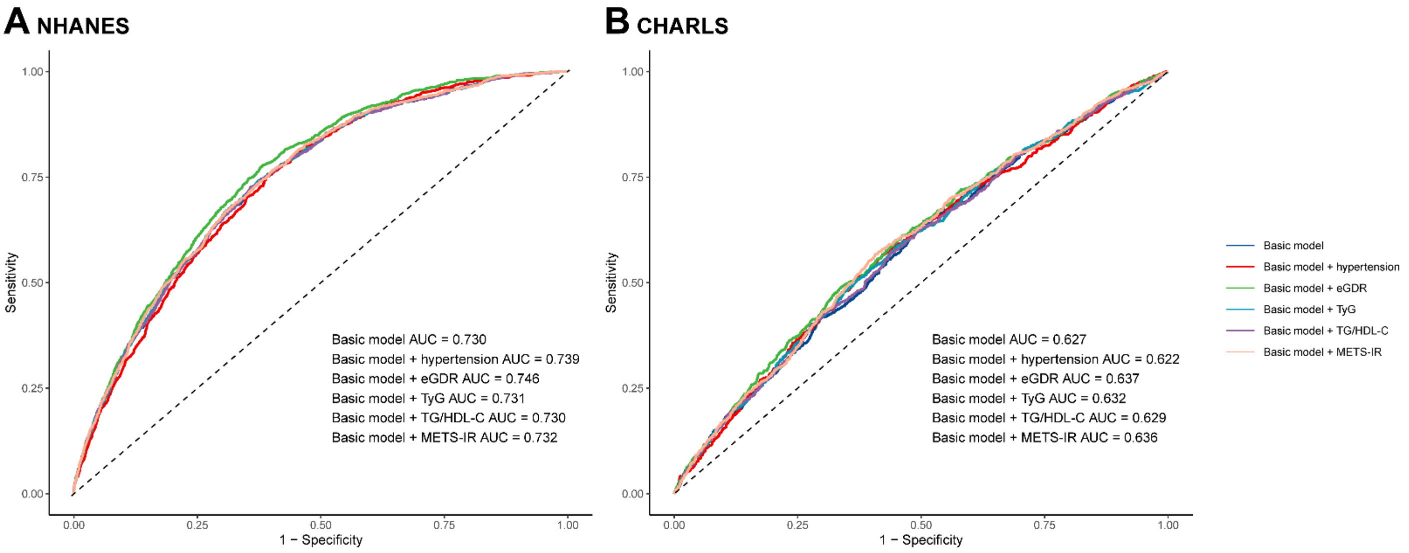

Supplementary Figure 12 The receiver operating characteristic curves of the eGDR, TyG, TG/HDL-C and METS-IR to predict stroke. The basic model adjusted age, sex, marital status, education, smoking, alcohol consumption status, total cholesterol, high-density lipoprotein cholesterol, triglyceride, low-density lipoprotein cholesterol, blood urea nitrogen, uric acid, hemoglobin, and obesity. eGDR, estimated glucose disposal rate; TyG, triglyceride glucose; HDL-C, high-density lipoprotein cholesterol; METS-IR, metabolic score for insulin resistance.

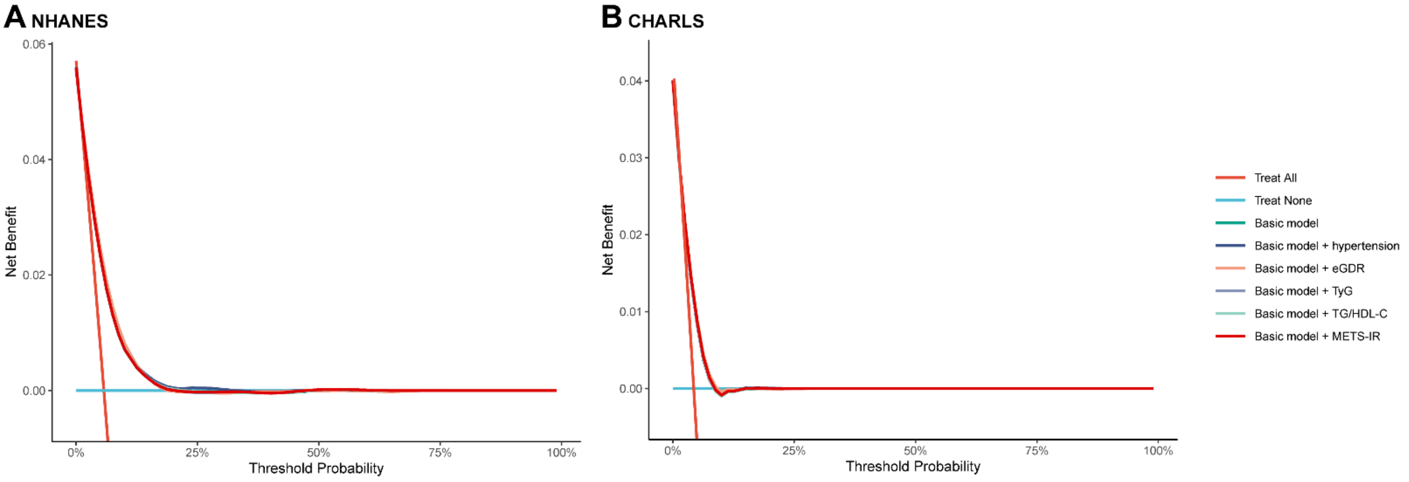

Supplementary Figure 13 The decision curve analysis of eGDR, TyG, TG/HDL-C and METS-IR to compare the clinical utility for stroke, the y-axis represents net benefits, calculated by subtracting the relative harm (false positives) from the benefits (true positives). The x-axis calculates the threshold probability. The basic model adjusted age, sex, marital status, education, smoking, alcohol consumption status, total cholesterol, high-density lipoprotein cholesterol, triglyceride, low-density lipoprotein cholesterol, blood urea nitrogen, uric acid, hemoglobin, and obesity. eGDR, estimated glucose disposal rate; TyG, triglyceride glucose; HDL-C, high-density lipoprotein cholesterol; METS-IR, metabolic score for insulin resistance.
